# Inhibition of cytoplasmic EZH2 induces antitumor activity through stabilization of the DLC1 tumor suppressor protein

**DOI:** 10.1038/s41467-021-26993-3

**Published:** 2021-12-03

**Authors:** Brajendra K. Tripathi, Meghan F. Anderman, Disha Bhargava, Luciarita Boccuzzi, Xiaolan Qian, Dunrui Wang, Marian E. Durkin, Alex G. Papageorge, Fernando J. de Miguel, Katerina Politi, Kylie J. Walters, James H. Doroshow, Douglas R. Lowy

**Affiliations:** 1 Laboratory of Cellular Oncology, Center for Cancer Research, National Cancer Institute, Bethesda, MD USA; 2 Yale Cancer Center, Yale School of Medicine, New Haven, CT USA; 3 Departments of Pathology and Internal Medicine (Section of Medical Oncology), Yale School of Medicine, New Haven, CT USA; 4 Structural Biophysics Laboratory, Center for Cancer Research, National Cancer Institute, Frederick, MD USA; 5 Developmental Therapeutics Branch, Center for Cancer Research, National Cancer Institute, Bethesda, MD USA

**Keywords:** Non-small-cell lung cancer, Methylation

## Abstract

mRNA expression of the *DLC1* tumor suppressor gene is downregulated in many lung cancers and their derived cell lines, with DLC1 protein levels being low or absent. Although the role of increased EZH2 methyltransferase in cancer is usually attributed to its histone methylation, we unexpectedly observed that post-translational destabilization of DLC1 protein is common and attributable to its methylation by cytoplasmic EZH2, leading to CUL-4A ubiquitin-dependent proteasomal degradation of DLC1. Furthermore, siRNA knockdown of *KRAS* in several lines increases DLC1 protein, associated with a drastic reduction in cytoplasmic EZH2. Pharmacologic inhibition of EZH2, CUL-4A, or the proteasome can increase the steady-state level of DLC1 protein, whose tumor suppressor activity is further increased by AKT and/or SRC kinase inhibitors, which reverse the direct phosphorylation of DLC1 by these kinases. These rational drug combinations induce potent tumor growth inhibition, with markers of apoptosis and senescence, that is highly dependent on DLC1 protein.

## Abstract (web summary)

The tumor suppressor gene DLC1 can be downregulated in cancers through genetic and non-genetic mechanisms. Here, the authors show cytoplasmic EZH2 can directly methylate and destabilize the DLC1 protein, and EZH2 inhibition can increase the DLC1 protein half-life at least 20-fold.

## Introduction

Identification of useful drug combinations for cancer treatment represents an important challenge for molecular targeted agents^1^. The focus of most molecular targeted agents has been to inhibit oncoproteins, especially mutationally activated ones^2^, with less consideration given to their effects on tumor suppressor proteins, although tumors usually arise from combined effects of increased oncoprotein activity and decreased tumor suppresssor activity^3^. However, there is considerable theoretical clinical potential for reactivating tumor suppressors when their activity has been reduced non-genetically. To date, the main clinically useful example of such reactivation has been decreased phosphorylation of the pRB tumor suppressor by CDK4/6 kinase inhibitors in breast cancer treatment^4^.

We have been studying the *DLC1* tumor suppressor gene, which encodes a cytoplasmic Rho-GAP (GTPase-activating protein) that catalyzes the hydrolysis of active Rho-GTP to inactive Rho-GDP^5,6^, and have identified several oncoprotein kinases—namely AKT, SRC (and SRC family kinases), and ERK—that directly phosphorylate and attenuate the Rho-GAP and tumor suppressor activities of the DLC1^7,8^. These observations raised the possibility that DLC1 protein reactivation by inhibitors of these kinases might have translational implications for cancer treatment. As the main direct DLC1-related function of ERK was to stimulate the binding of SRC to DLC1, leading to increased DLC1 phosphorylation by SRC, we focused on AKT and SRC inhibitors. In our preclinical studies, inhibitors of these two kinases had a strong antitumor activity that depended on DLC1 protein expression, although both AKT and SRC have many biochemical targets.

One limitation of this therapeutic approach is that it is only likely to benefit tumors with moderate to high levels of DLC1 protein, and DLC1 expression is frequently downregulated in a variety of cancers through genetic and non-genetic mechanisms^6,9–13^. The latter category includes primary lung adenocarcinomas (LUAD) and lung cancer cell lines that express wild-type *DLC1* mRNA but lack detectable DLC1 protein. This phenotype is present both in clinical biospecimens and several mutant *KRAS* lung cancer lines whose DLC1 protein levels are regulated by activation of the Cullin 4A (CUL-4A) ubiquitin E3 ligase and subsequent proteasome-dependent degradation of DLC1 protein^14^. One possible way to increase the proportion of tumors for which the therapeutic targeting of DLC1 protein could be clinically beneficial might be to use a suitable inhibitor to reverse this post-translational regulation, which has not been explored previously.

We initiated the current study by screening for drugs in addition to proteasome inhibitors^15^ that might increase DLC1 protein levels, as they could lead to identification of additional vulnerabilities that might increase our understanding of the pathways regulating DLC1 expression and might have therapeutic application. This screen unexpectedly determined that the DLC1 protein was stabilized by inhibitors of EZH2, the catalytic component of the polycomb repressor complex 2 (PRC2) that is a predominantly nuclear lysine methyltransferase frequently overexpressed or mutated in cancer^16,17^. This finding has led us to determine that DLC1 protein is a direct substrate of cytoplasmic EZH2, whose monomethylation of DLC1 leads to its destabilization. The post-translational regulation of DLC1 by cytoplasmic EZH2 differs from its canonical nuclear epigenetic regulation of gene expression by trimethylation of histone H3 on Lysine 27 (H3K27).

The relationship between DLC1 and EZH2 led us to ask whether there might be a previously unknown link between DLC1 protein and *KRAS*, as mutant *KRAS* has been reported to upregulate *EZH2* expression in experimental pancreatic cancer^18^ and LUAD cell lines^19^. This question has potential translational relevance, given that there is no clinically proven treatment for most tumors with mutant *KRAS*, although it has been known for many years that it is commonly mutated in pancreatic cancer, LUAD, and colorectal cancer^20^. We determined that decreasing the expression of wild-type or mutant *KRAS* increased the level of DLC1 protein.

In addition to reporting that DLC1 protein is a new critical substrate for cytoplasmic EZH2, this study provides a rational approach for combination molecular targeted agent cancer treatment by non-genetically reactivating a tumor suppressor protein, leading to potent antitumor activity. It is relevant to tumors that express wild-type *DLC1* mRNA, whether their steady-state level of DLC1 protein, prior to molecular targeted agent treatment, is detectable or undetectable. Our observations suggest EZH2 inhibitors should be considered for their potential impact on post-translational protein regulation in addition to their regulation of transcription. Moreover, the combination therapy identified here has preclinical efficacy against tumor lines with mutant *KRAS*.

## Results

### Inhibitors of EZH2 and the proteasome increase steady-state DLC1 protein

**Fig. 1:**
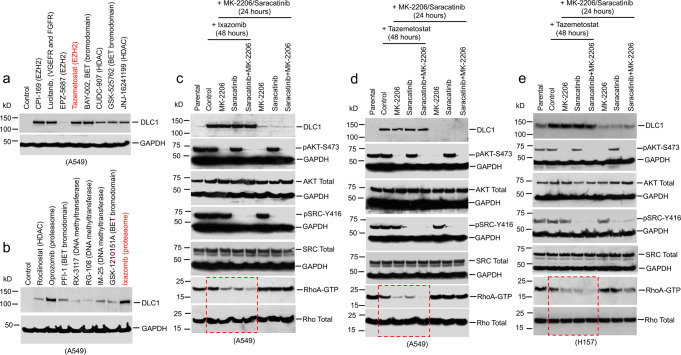
EZH2 and proteasome inhibitors increase DLC1 protein in A549 and H157 NSCLC lines, whose Rho-GAP activity is increased by AKT and SRC inhibitors. **a**, **b** Steady-state DLC1 protein levels in A549 LUAD cells after 48 h treatment with the indicated inhibitors. GAPDH was used as a loading control. **c**, **d** Treatment with Ixazomib (**c**) or Tazemetostat (**d**) increases DLC1 protein in A549 cells. MK-2206 and/or Saracatinib treatment decreases RhoA-GTP in Ixazomib or Tazemetostat treated cells, but not in parental DLC1 protein-negative cells, although MK-2206 and Saracatinib inhibit AKT activity (measured by pAKT-S473) and SRC activity (measured by pSRC-Y416) in all treated samples. Combined treatment of MK-2206 and Saracatinib with Ixazomib or Tazemetostat further reduces RhoA-GTP. Cells were treated with Ixazomib or Tazemetostat for 48 h, with MK-2206 and Saracatinib added during the last 24 h period. **e** Steady-state DLC1 protein levels in H157 LUAD cells after 48 h treatment with Tazemetostat. MK-2206 and/or Saracatinib treatment decreases RhoA-GTP in parental DLC1 protein-positive cells. Tazemetostat treatment by itself increased the level of DLC1 protein, and the AKT and/or SRC kinase inhibitors in the cells containing Tazemetostat reduced RhoA-GTP to an even greater degree than in the absence of Tazemetostat. Two independent experiments were performed for **a**–**e** with similar results. Source data are provided as a Source Data file.

To screen for pharmacologic inhibitors of transcriptional and post-transcriptional targets that might increase the steady-state level of DLC1 protein, we used the A549 LUAD line, which expresses *DLC1* mRNA but does not have readily detectable DLC1 protein. Relatively high DLC1 protein levels were seen following treatment with two out of three EZH2 inhibitors, several proteasome inhibitors, one bromodomain inhibitor, and lucitanib, which inhibits both VEGFR and FGFR (Fig. 1a, b). Lower DLC1 protein levels were induced by DNA methyltransferase inhibitors and histone deacetylase inhibitors. DLC1 mRNA expression was increased less than two-fold by the inhibitors (Supplementary Fig. 1A), implying post-transcriptional mechanisms accounted for most of the increases in DLC1 protein.

To be able to analyze other cell lines in subsequent experiments, we screened a panel of non-small cell lung cancer (NSCLC) lines for their expression of *DLC1* mRNA and protein under regular growth conditions. *DLC1* mRNA was readily detected in each line, except for H358, which, as reported previously^21^, does not express *DLC1* mRNA (Supplementary Fig. 1B). In addition to A549, three other NSCLC lines did not contain readily detectable DLC1 protein, while it was present in two others (Supplementary Fig. 1C).

### AKT and SRC inhibitors reactivate Rho-GAP activity of DLC1 protein stabilized by proteasome and EZH2 inhibitors

We decided to focus on two classes of inhibitors found to induce high DLC1 protein levels, EZH2 and proteasome inhibitors, with greater emphasis given to the former because its effect on DLC1 was less expected. We evaluated the EZH2 inhibitor Tazemetostat, which was FDA-approved in 2020 for other indications^22,23^, and the proteasome inhibitor Ixazomib, which is FDA-approved with combination treatment for relapsed or refractory multiple myeloma^24^. Our previous findings that AKT and SRC kinase inhibitors could cooperate with each other to reactivate the Rho-GAP and tumor suppressor activities of DLC1 in lung cancer lines expressing DLC1 protein^8^ implied it might be beneficial to use these two inhibitors singly or together in conjunction with Ixazomib or Tazemetostat for treatment.

To evaluate the effect of proteasome inhibition on intracellular RhoA-GTP, which can serve as a surrogate for DLC1 tumor suppressor activity, A549 cells were treated for 24 h with Ixazomib alone followed by another 24 h of Ixazomib treatment together with the AKT inhibitor MK-2206 and/or the SRC inhibitor Saracatinib, and the RhoA-GTP level was determined (Fig. 1c). Ixazomib treatment alone increased DLC1 protein, but had a modest effect on RhoA-GTP, as both AKT and SRC kinases, which attenuate DLC1 RhoA-GAP activity, are active in this cell line^7,8^. However, Ixazomib treatment with either MK-2206 or Saracatinib induced a substantial decrease in RhoA-GTP, and treatment with all three drugs induced an even greater reduction. By contrast, treatment with MK-2206 and/or Saracatinib alone did not result in readily detectable DLC1 protein or a reduction in RhoA-GTP, although the inhibitors reduced their respective kinase activities, as monitored by decreased phosphorylation of pAKT-S473 and pSRC-Y416.

Qualitatively similar results were seen when Tazemetostat was used in place of Ixazomib (Fig. 1d). Similar reductions of RhoA-GTP by Tazemetostat and the kinase inhibitors were observed in three other lung cancer lines (NCI-H23, NCI-H460, and SW900) that express *DLC1* mRNA but lack detectable DLC1 protein (Supplementary Fig. 1B–F). Other AKT and tyrosine kinase inhibitors, Perifosine and Bosutinib, respectively, when used in combination with Tazemetostat in A549 cells, also reduced RhoA-GTP similarly to MK-2206 and Saracatinib (Supplementary Fig. 1G).

The ability of Tazemetostat to increase DLC1 protein levels in a line that contains endogenous DLC1 protein was evaluated with H157 cells. As shown previously^8^, in the absence of Tazemetostat, AKT and/or SRC kinase inhibition reduced RhoA-GTP (Fig. 1e, right lanes). However, Tazemetostat treatment by itself increased the level of DLC1 protein, and the AKT and/or SRC kinase inhibitors in the cells containing Tazemetostat reduced RhoA-GTP to an even greater degree than in the absence of Tazemetostat (Fig. 1e, left lanes). Thus, EZH2 inhibition can also increase the DLC1 protein level in a line with detectable DLC1 protein prior to treatment, and the upregulated DLC1 protein can further increase negative RhoA-GTP regulation by the kinase inhibitors.

### AKT and SRC inhibitors cooperate with proteasome or EZH2 inhibition in reducing anchorage-independent and tumor xenograft growth

**Fig. 2:**
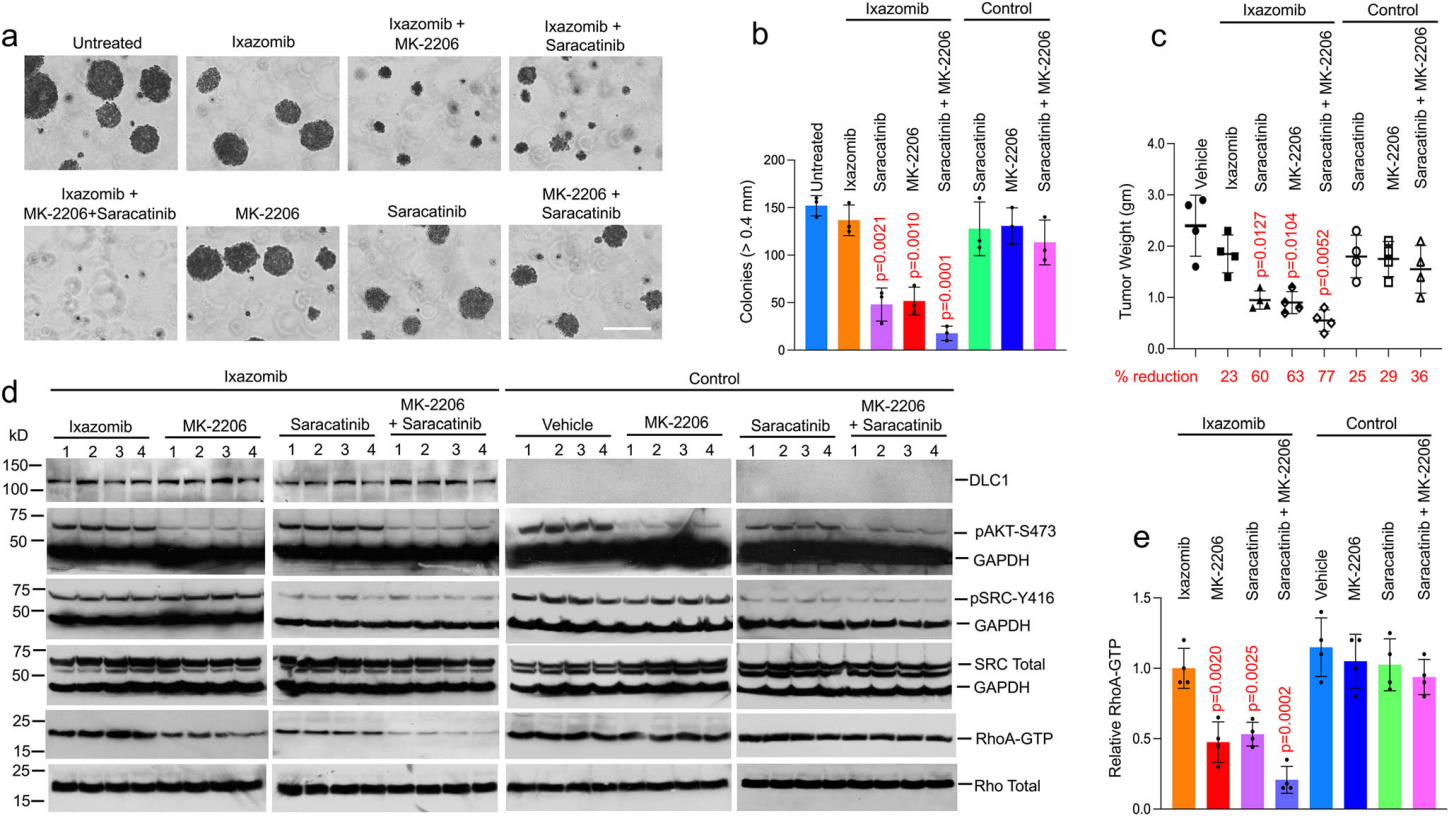
Saracatinib and MK-2206 cooperate with Ixazomib in reducing anchorage-independent and tumor xenograft growth. **a**, **b** Anchorage-independent growth of A549 cells treated with Ixazomib, MK-2206, and/or Saracatinib for 3 weeks. **a** In the presence of Ixazomib, MK-2206, or Saracatinib treatment inhibited colony formation in soft agar. Bar, 2 mm. **b** Quantification of agar colonies in **a** (>0.4 mm). For each treatment group, mean and standard deviation (SD) are shown. *N* = 3 independent experiments. Error bars represent SD. The combined treatment of MK-2206 and/or Saracatinib together with Ixazomib induced a significant decrease in colony number. However, the decrease was not significant with MK-2206 and/or Saracatinib treatment alone or Ixazomib alone. For the statistical analysis, a parametric unpaired *t*-test with Welch’s correction was performed for **b**, **c**, and **e** using Prism software. The statistical test was two-sided, and no adjustments were made for multiple comparisons. *p* = 0.0021 for untreated versus Ixazomib + Saracatinib, *p* = 0.0010 for untreated versus Ixazomib + MK-2206, and *p* = 0.0001 for untreated versus Ixazomib + Saracatinib + MK-2206 treatment, respectively. (**c**–**e**) Response of mouse A549 xenograft tumors to treatment with Ixazomib (3 weeks) and MK-2206 and/or Saracatinib (2 weeks). **c** For each treatment group, individual tumor weights, mean, and SD are shown. Error bars represent SD. The numbers at the bottom represent the percent reduction for each treatment group compared with the untreated vehicle control. *N* = 4 mice per group. *p* = 0.0127 for vehicle versus Ixazomib + Saracatinib, *p* = 0.0104 for vehicle versus Ixazomib + MK-2206, and *p* = 0.0052 for vehicle versus Ixazomib + Saracatinib + MK-2206 treatment, respectively. **d** MK-2206 or Saracatinib treatment of tumors efficiently inhibited AKT (pAKT-S473) and SRC (pSRC-Y416) activities, but only reduced RhoA-GTP when treated with Ixazomib (Ixazomib treatment group, first and second panels), and not in DLC1-negative (control, third and fourth panels). Treatment with all three drugs reduced RhoA-GTP to a greater degree than two drugs. **e** Graph shows mean RhoA-GTP ± SD within each group. Error bars represent SD. *N* = 4 mice tumors. *p* = 0.0020 for vehicle versus Ixazomib + MK-2206, *p* = 0.0025 for vehicle versus Ixazomib + Saracatinib, and *p* = 0.0002 for vehicle versus Ixazomib + MK-2206 + Saracatinib treatment, respectively. Source data are provided as a Source Data file.

**Fig. 3:**
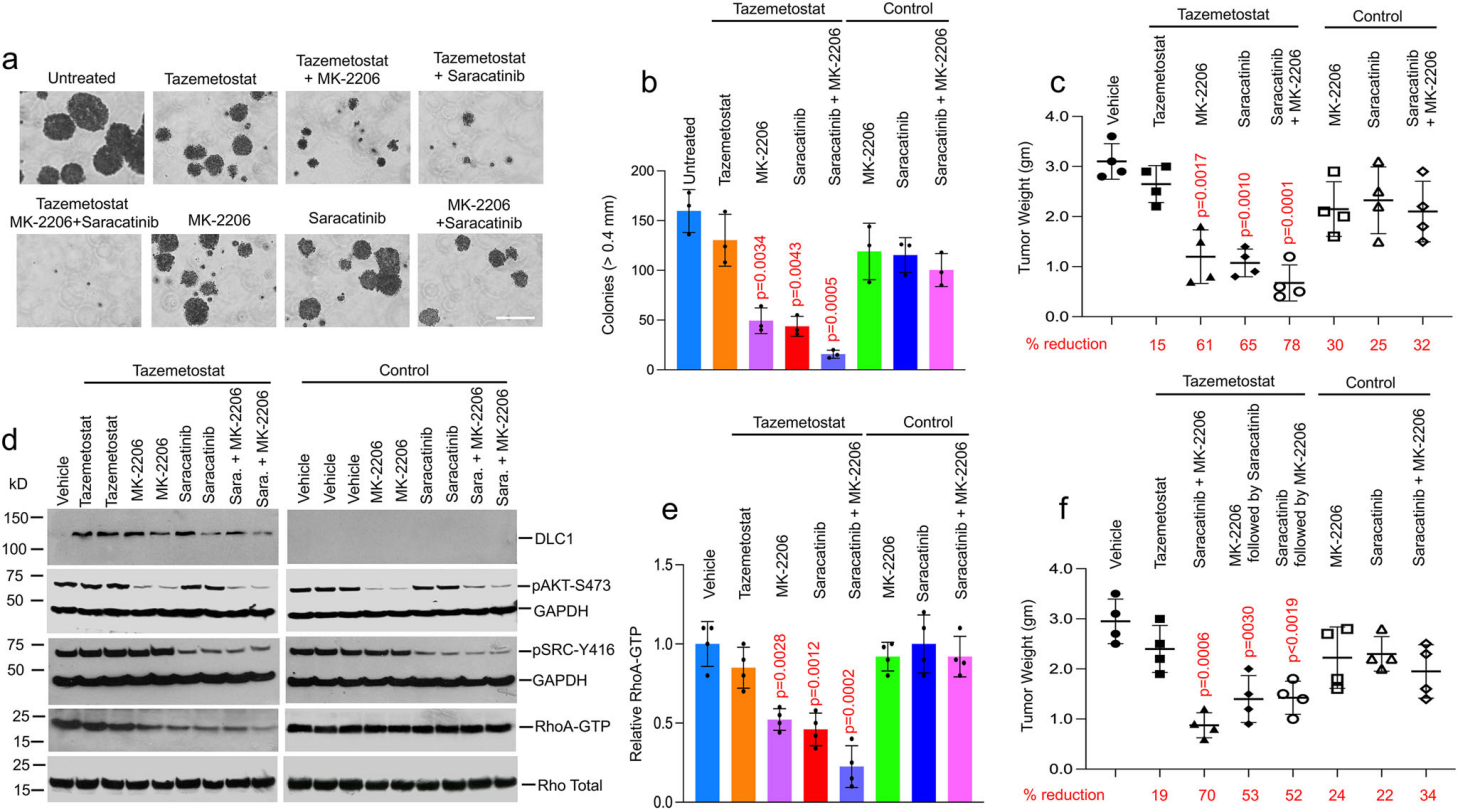
Saracatinib and MK-2206 cooperate with Tazemetostat in reducing anchorage-independent and tumor xenograft growth. **a**, **b** Anchorage-independent growth of A549 cells after 3 weeks treatment with Tazemetostat and MK-2206 and/or Saracatinib. **a** In the presence of Tazemetostat, MK-2206 and/or Saracatinib treatment inhibited growth and colony formation in soft agar. Bar, 2 mm. **b** Quantification of agar colonies in **a** (>0.4 mm). For each group, mean and SD are shown. *N* = 3 independent experiments. Error bars represent SD. **c**–**e** Response of mouse A549 xenograft tumors to treatment with Tazemetostat (3 weeks) and MK-2206 and/or Saracatinib (2 weeks). For the statistical analysis, a parametric unpaired *t*-test with Welch’s correction was performed for **b**, **c**, **e**, and **f** using Prism software. The statistical test was two-sided, and no adjustments were made for multiple comparisons. *p* = 0.0034 for untreated versus Tazemetostat + MK-2206, *p* = 0.0043 for untreated versus Tazemetostat + Saracatinib, and *p* = 0.0005 for untreated versus Tazemetostat + MK-2206 + Saracatinib treatment, respectively. **c** For each treatment group, individual tumor weights, mean, and SD are shown. Error bars represent SD. The numbers below the graph represent the percent reduction in tumor weight for each treatment group compared with the untreated vehicle control. *N* = 4 mice per group. *p* = 0.0017 for vehicle versus Tazemetostat + MK-2206, *p* = 0.0010 for vehicle versus Tazemetostat + Saracatinib, and *p* = 0.0001 for vehicle versus Tazemetostat + MK-2206 + Saracatinib treatment, respectively. **d** MK-2206 or Saracatinib treatment of tumors efficiently inhibited AKT and SRC activities but only reduced RhoA-GTP in DLC1-positive tumors (Tazemetostat treatment group, first panel), and not in DLC1-negative tumors (control, second panel). Treatment with all three drugs reduced RhoA-GTP to a greater degree than two drugs. **e** Graph shows mean RhoA-GTP ± SD within each group. Error bars represent SD. *N* = 4 mice tumors. *p* = 0.0028 for vehicle versus Tazemetostat + MK-2206, *p* = 0.0012 for vehicle versus Tazemetostat + Saracatinib, and *p* = 0.0002 for vehicle versus Tazemetostat + MK-2206 + Saracatinib treatment, respectively. **f** Tumor xenograft treatment with two inhibitors per week. Mice with tumors were treated with Tazemetostat for a total of 3 weeks. In the first week, they were treated only with Tazemetostat, and in the second week, they were treated with either MK-2206 or Saracatinib with Tazemetostat. In the third week, they continued to be treated with Tazemetostat but were switched to Saracatinib if they had received MK-2206 in the second week or to MK-2206 if they had received Saracatinib in the second week. For each treatment group, individual tumor weights, mean, and SD are shown. Error bars represent SD. The positive control was treated with all three inhibitors during the second and third weeks. The numbers below the graph represent the percent reduction in tumor weight for each treatment group compared with the untreated vehicle control. *N* = 4 mice per group. *p* = 0.0006 for vehicle versus Tazemetostat + MK-2206 + Saracatinib, *p* = 0.0030 for vehicle versus Tazemetostat + Saracatinib treatment for second week, and *p* = 0.0019 for vehicle versus Tazemetostat + MK-2206 treatment for second week, respectively. Source data are provided as a Source Data file.

We next examined the impact of inhibitor treatment on two biological parameters, anchorage-independent growth and xenograft tumor growth in immunosuppressed mice. For anchorage-independent growth, A549 cells were grown in agar for three weeks in the absence or presence of the inhibitors. For the A549 xenografts, when tumor diameters were ~0.5 cm, mice were treated for a first week with either Ixazomib or Tazemetostat followed by an additional two weeks with the same inhibitor used during the first week together with MK-2206 and/or Saracatinib. In parallel with the observed changes in RhoA-GTP, MK-2206 and Saracatinib cooperated with Ixazomib (Fig. 2) or with Tazemetostat treatment (Fig. 3) in reducing cell growth in agar and xenografts. The smaller colony size in agar (Figs. 2a, b and 3a, b) and lower xenograft tumor weight (Figs. 2c and 3c) were dependent on treatment with Ixazomib or Tazemetostat in combination with either MK-2206 or Saracatinib, with the three inhibitors together producing the greatest reduction. By contrast, single-agent treatment or combined treatment with MK-2206 and Saracatinib had only limited growth-inhibitory effects. Reductions in RhoA-GTP in the residual xenografts paralleled the reductions in tumor weight (Figs. 2d, e and 3d, e). As expected, tissue sections from treated tumors had higher DLC1 protein after Ixazomib or Tazemetostat treatment (Supplementary Fig. 2A–D). Markers of senescence and apoptosis, β-galactosidase and Annexin V, respectively, were also seen (Supplementary Fig. 3 and 4). Their frequency paralleled the decreases in tumor weight, with the three-drug combination resulting in the highest proportion of cells expressing either marker in tumors.

Although the three-drug xenograft treatment with Tazemetostat was not associated with obvious side effects, such as weight loss (Supplementary Fig. 5A, B), greater toxicity might be seen in people with this regimen compared with a two-drug combination. Therefore, we explored an alternative sequential 3-week treatment approach, administering only two of the three drugs in any given week. When tumors reached approximately 0.5 cm, mice were treated with Tazemetostat during week 1 followed by treatment with Tazemetostat together with MK-2206 or Saracatinib during week two. For week 3, they continued to be treated with Tazemetostat but were switched to Saracatinib if they had received MK-2206 or to MK-2206 if they had received Saracatinib. Compared with the untreated controls, the tumor weight was ~50% lower with the two-drug sequential regimen regardless of the order in which MK-2206 and Saracatinib were given, while the three-drug regimen resulted in a 70% decrease (Fig. 3f).

### DLC1 is a critical target of a three-drug regimen

**Fig. 4:**
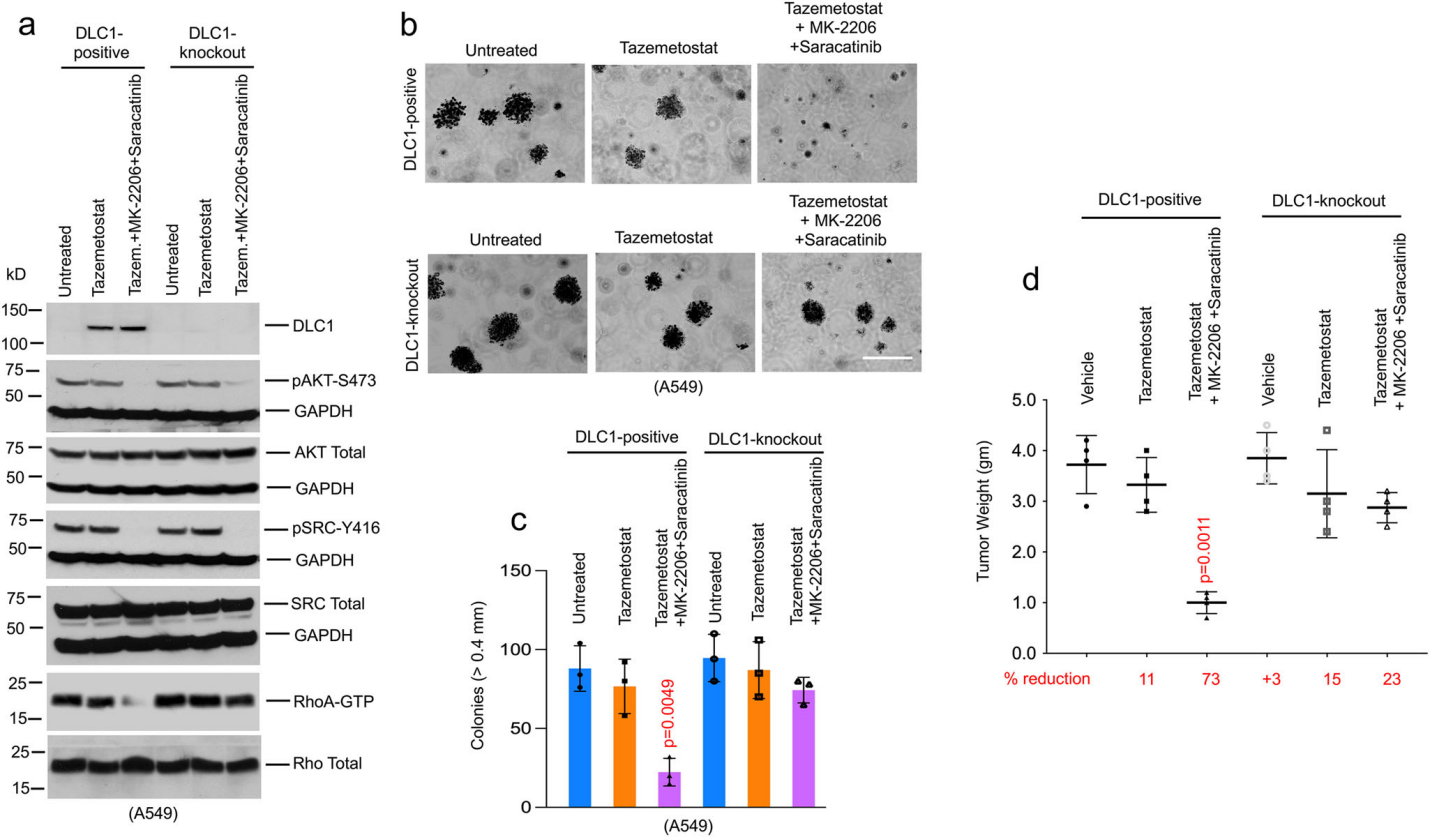
DLC1 is a critical target of a three-drug combination. **a** Steady-state DLC1 protein levels in A549 LUAD cells after Tazemetostat treatment, which increases DLC1 protein in DLC1-positive A549 cells, but not in the isogenic DLC1-knockout cells. Combined treatment of MK-2206 and Saracatinib decreases RhoA-GTP in Tazemetostat treated DLC1-positive cells, but not in the isogenic DLC1-knockout cells, although MK-2206 and Saracatinib inhibited AKT activity (measured by pAKT-S473) and SRC activity (measured by pSRC-Y416) in all treated samples. Two independent experiments were performed with similar results. **b**, **c** Knockout of DLC1 expression abrogates most of the anchorage-independent growth inhibition of A549 cells treated with Tazemetostat, MK-2206, and/or Saracatinib for 3 weeks. **b** Colony growth with DLC1-positive or DLC1-knockout A549 cells. Bar, 2 mm. **c** Quantification of agar colonies in **b** (>0.4 mm). *N* = 3 independent experiments. Error bars represent SD. For the statistical analysis, a parametric unpaired *t*-test with Welch’s correction was performed for **c** and **d** using Prism software. The statistical test was two-sided, and no adjustments were made for multiple comparisons. *p* = 0.0049 for untreated versus Tazemetostat + MK-2206 + Saracatinib treatment in DLC1-positive A549 cells. **d** Response of mouse A549 xenograft tumors to treatment with Tazemetostat, MK-2206, and Saracatinib. *N* = 4 mice per group. For each treatment group, individual tumor weights, mean, and SD are shown. Error bars represent SD. The numbers below the graph represent the percent reduction in tumor weight for each treatment group compared with the untreated vehicle control. Combined treatment with Tazemetostat, MK-2206, and Saracatinib efficiently reduced xenografts growth in DLC1-positive tumors, but not in the isogenic DLC1-knockout tumors. *p* = 0.0011 for vehicle versus Tazemetostat + MK-2206 + Saracatinib treatment in DLC1-positive tumors. Source data are provided as a Source Data file.

To determine if DLC1 protein is a mechanistically important target of the inhibitors, we evaluated this question in A549 cells, preliminarily by siRNA DLC1 knockdown and then by CRISPR-Cas9-mediated *DLC1* gene editing (DLC1-knockout). When the siRNA knockdown cells were analyzed for their growth in agar, most of the growth inhibition by the three-drug combination of Tazemetostat, MK-2206, and Saracatinib was abolished, suggesting that DLC1 was a key target of the combination (Supplementary Fig. 5C–E). As expected, the DLC1-knockout cells remained negative for DLC1 protein when treated with Tazemetostat, and the three-drug combination had almost no impact on RhoA-GTP level (Fig. 4a). Remarkably, most of the reductions in anchorage-independent growth (Fig. 4b, c) and xenograft tumor growth (Fig. 4d) induced by the combination were found to require DLC1 expression, strongly implying that DLC1 protein makes a critical contribution to the growth inhibition observed with the three-drug combination. The drug combination was not associated with obvious side effects, such as changes in mouse fur or weight loss (Supplementary Fig. 6).

To assess the role of endogenous DLC1 protein on the three-drug combination in a line that expresses detectable levels under regular growth conditions, we used CRISPR-Cas9 technology to disrupt the DLC1 gene in NSCLC H157 cells. As with the A549 cells, the regulation of RhoA-GTP and most of the anchorage-independent growth inhibition induced by the drug combination depended on DLC1 expression (Supplementary Fig. 7).

### Inhibition of EZH2 and NAE reduces RhoA-GTP in a DLC1-dependent manner

**Fig. 5:**
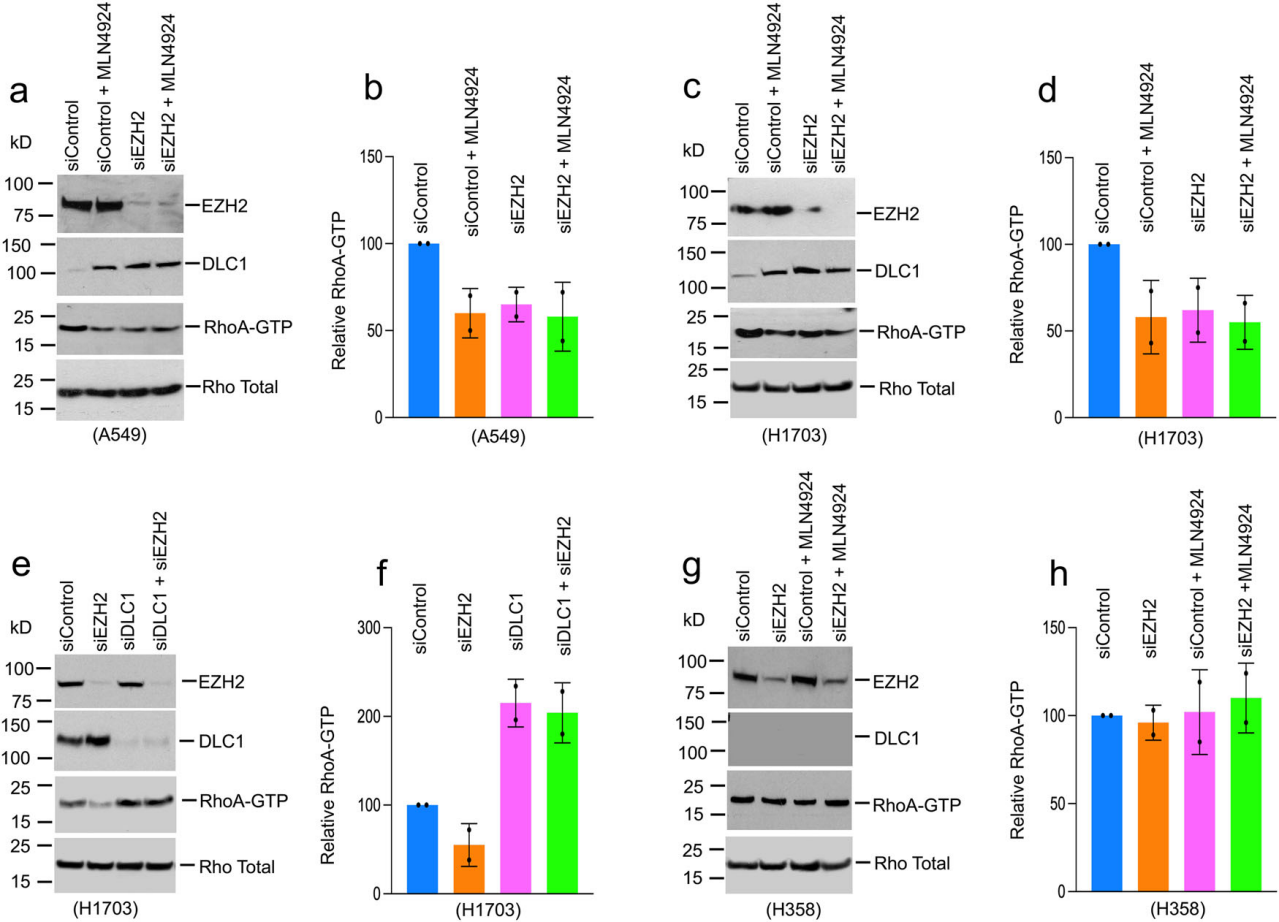
Reduction of RhoA-GTP by the inhibition of EZH2 and NAE is DLC1-dependent. **a**–**d** Treatment of A549 cells (**a**, **b**) and H1703 cells (**c**, **d**) with NAE inhibitor MLN4924 or EZH2 siRNAs increased DLC1 protein and reduced RhoA-GTP. Combined treatment with MLN4924 and EZH2 siRNAs did not further increase the response. **b** and **d** Quantification of mean RhoA-GTP ± SD from three experiments, as shown in **a** and **c**. Error bars represent SD. **e**, **f** siRNAs knockdown of DLC1 expression abolished the ability of the EZH2 siRNAs to regulate RhoA-GTP. **f** Quantification of mean RhoA-GTP ± SD from three experiments, as shown in **e**. Error bars represent SD. **g**, **h** EZH2 siRNAs and MLN4924 treatment of H358 cells, a *DLC1* mRNA-negative line, did not lead to DLC1 protein detection or a change in RhoA-GTP. **h** Quantification of mean RhoA-GTP ± SD from three experiments, as shown in **g**. Error bars represent SD. Source data are provided as a Source Data file.

The above findings provided a strong rationale for exploring the mechanism by which Tazemetostat exerts its effects. To rule out the possibility of an off-target effect for Tazemetostat, we used EZH2-specific siRNAs to knockdown EZH2 expression in A549 cells. As with Tazemetostat, reduced EZH2 expression increased the steady-state level of DLC1 protein and produced a modest reduction in RhoA-GTP (Fig. 5a, b). Furthermore, inhibition of NEDD8, a major activator of CUL-4A^25^, stabilized the DLC1 protein. Cells were treated with MLN4924, an inhibitor of NAE, the NEDD8-activating enzyme, which increased DLC1 protein and reduced RhoA-GTP (Fig. 5a, b), confirming CUL-4A is the main ubiquitin E3 ligase whose activation leads to undetectable DLC1 protein^14^.

To assess whether inhibition of both NAE and EZH2 might have additive effects on DLC1 protein, cells were treated simultaneously with MLN4924 and EZH2 siRNA knockdown. However, the combined treatment did not increase DLC1 protein beyond that of single-agent treatment (Fig. 5a, b), suggesting NAE and EZH2 lie in the same DLC1 stabilization pathway. We also explored the effects of these two inhibitors in H1703 LUAD cells, which express detectable levels of DLC1 protein. The results were qualitatively similar to those of A549 cells: NAE inhibition or EZH2 siRNAs knockdown increased DLC1 protein and modestly decreased RhoA-GTP, but the combined treatment did not lead to a further change in RhoA-GTP (Fig. 5c, d). The ability to regulate RhoA-GTP levels in H1703 cells depended on their expression of DLC1, as siRNAs knockdown of DLC1 expression abolished the ability of the EZH2 siRNAs to affect RhoA-GTP (Fig. 5e, f). By contrast, EZH2 siRNAs and MLN4924 treatment of H358 cells, which do not express *DLC1* mRNA, did not lead to DLC1 protein detection or a change in RhoA-GTP (Fig. 5g, h).

### DLC1 forms a complex with EZH2 in the cytoplasm

DLC1 is mainly a cytoplasmic protein^7^, while the impact of EZH2 on cancer is widely attributed to its canonical epigenetic regulation of gene expression resulting from its methylation of H3K27 in the nucleus^17,26^. It was therefore unclear whether the effect of EZH2 on DLC1 protein levels might be attributable to a change in expression of a putative mRNA resulting from its H3K27 methylation or a more direct effect on DLC1 protein attributable to cytoplasmic EZH2. As cytoplasmic EZH2 protein has been identified in some cancer types^27–29^, we tested the latter hypothesis. By confocal microscopy, there was substantial cytoplasmic EZH2 protein in A549 cells, ~32% of the total EZH2 signal, while it was ~15% in non-transformed H1634 cells (Supplementary Fig. 8A, B). Perhaps more important, A549 cells contained more than six times as much cytoplasmic EZH2 protein as H1634 cells, as the total EZH2 signal in A549 cells was more than three times that in H1634 cells. To determine whether cytoplasmic EZH2 is frequent in lung cancer lines, we evaluated six additional lines that express *DLC1* mRNA, three of which resemble A549 in not having readily detectable levels of endogenous DLC1 protein. All six lines were found to express more total EZH2 and contain substantially more cytoplasmic EZH2 than H1634 cells (Supplementary Fig. 8A, B).

**Fig. 6:**
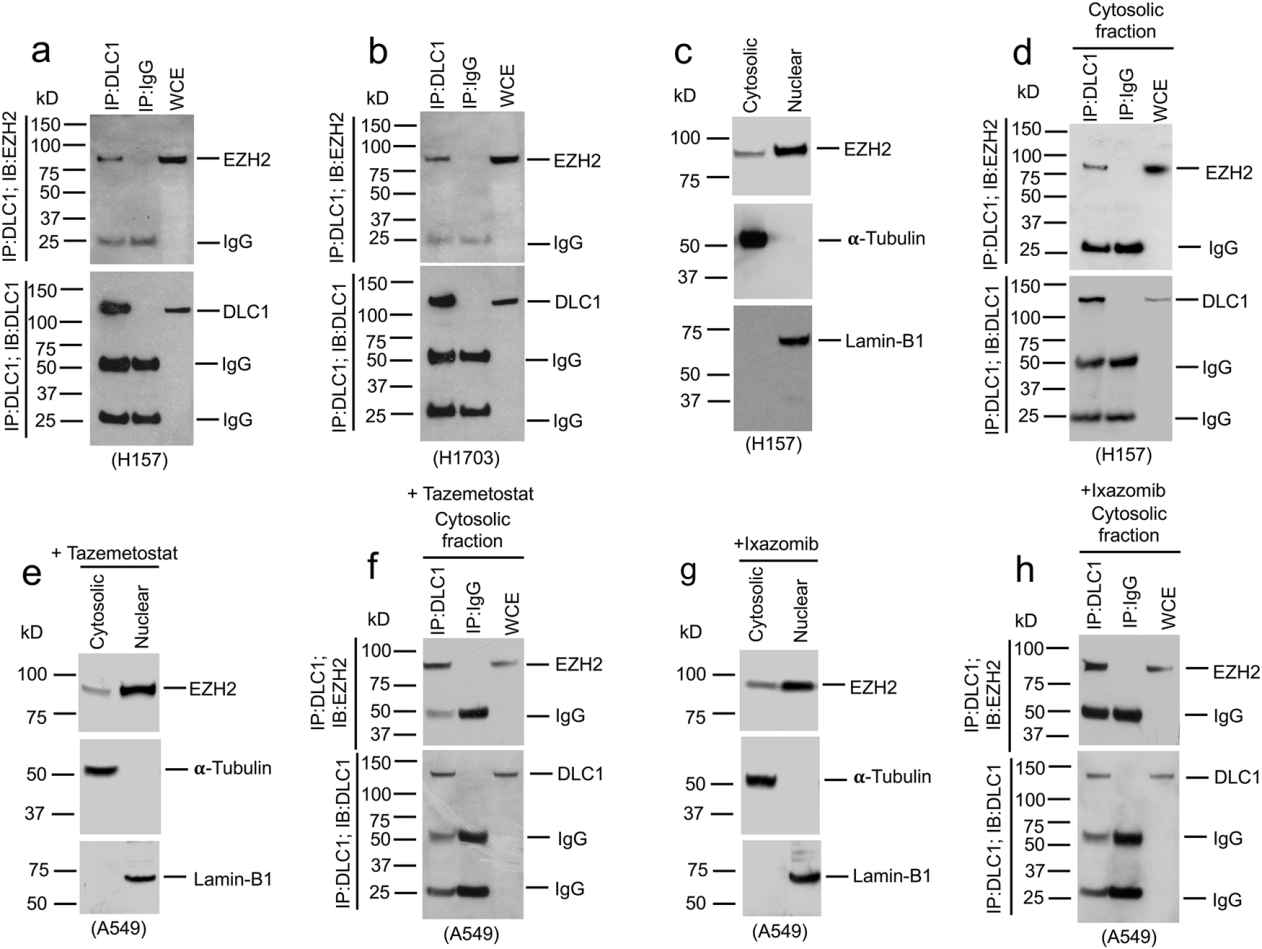
Cytoplasmic EZH2 binds with DLC1. **a**, **b** Complex formation between DLC1 and EZH2. Lysates from H157 (**a**) and H1703 (**b**) cells were IP with DLC1, or mock IgG antibodies followed by IB with EZH2 (top) or DLC1 (bottom) antibodies. WCE, whole cell extract. **c**, **d** Complex between DLC1 and EZH2 occurred in the cytoplasm of H157 cells. **c** Nuclear (Lamin-B1) and cytosolic (α-Tubulin) marker proteins confirmed the fractionation of H157 cells. **d** Extracts from cytosolic fraction of H157 were IP with DLC1 or mock IgG antibodies followed by IB with EZH2 antibodies (top) or DLC1 (bottom) antibodies. **e**–**h** Treatment of A549 cells with Tazemetostat (**e**, **f**) or Ixazomib (**g**, **h**) induced a cytoplasmic EZH2-DLC1 protein complex. A549 cells were treated with Tazemetostat or Ixazomib. Nuclear (Lamin-B1) and cytosolic (α-Tubulin) marker proteins confirmed the fractionation of A549 cells after treatment with Tazemetostat (**e**) or Ixazomib (**g**). **f**, **h** Extracts from cytosolic fraction of A549 treated with Tazemetostat (**f**) or Ixazomib (**h**) were IP with DLC1, or mock IgG antibodies followed by IB with EZH2 antibodies (top) or DLC1 (bottom) antibodies. Two independent experiments were performed for **a**–**h** with similar results. Source data are provided as a Source Data file.

To establish whether EZH2 and DLC1 form an endogenous protein complex in vivo, we performed co-immunoprecipitation (co-IP) experiments from two LUAD lines, H157 and H1703 which express both proteins. Co-IP experiments showed EZH2 formed a complex with DLC1 in both lines (Fig. 6a, b) that was confirmed to be cytoplasmic in H157 cells (Fig. 6c, d). Treatment of A549 cells with Tazemetostat (Fig. 6e, f) or Ixazomib (Fig. 6g, h) also induced a cytoplasmic EZH2-DLC1 protein complex.

**Fig. 7:**
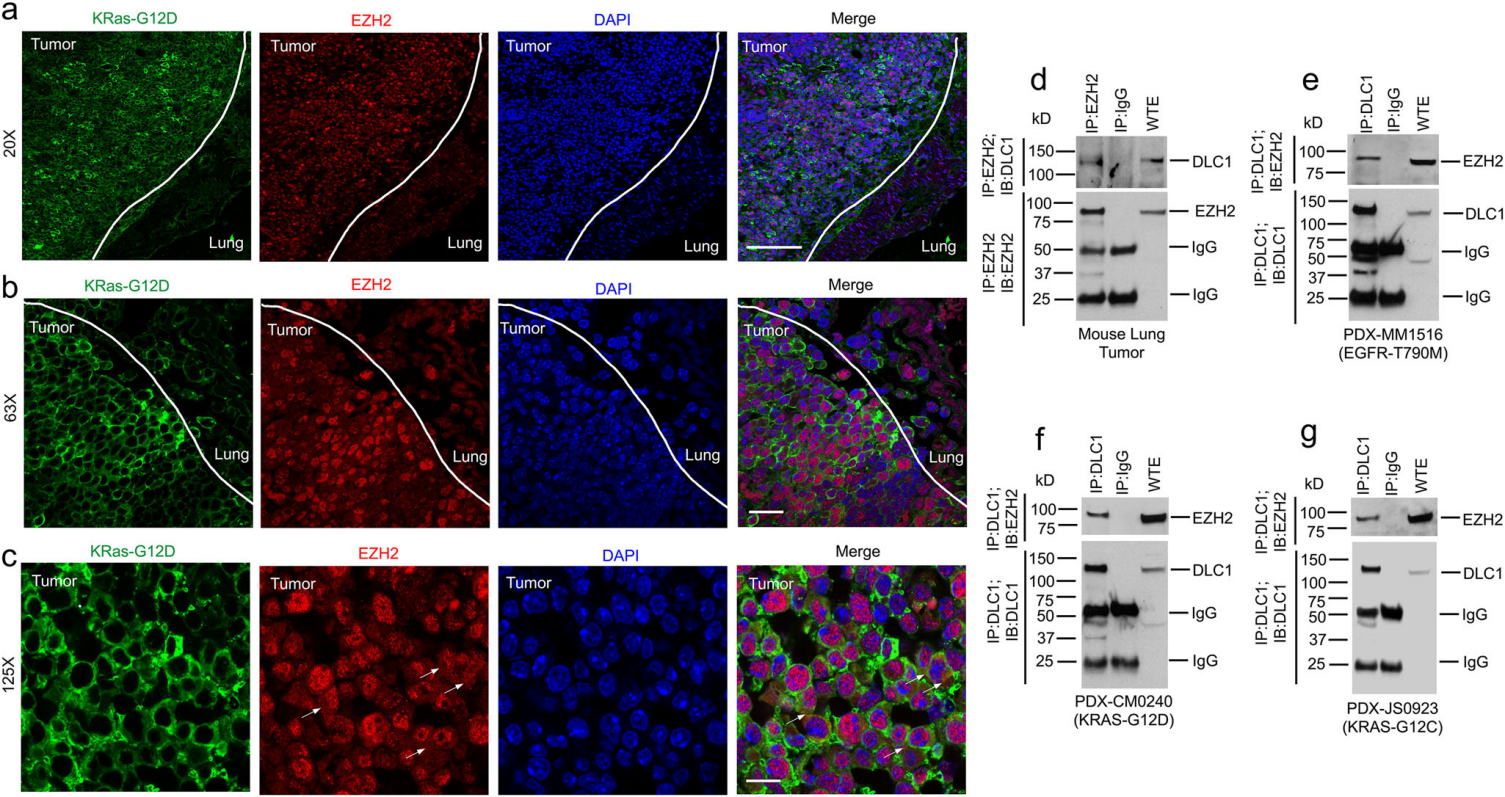
NSCLC models have cytoplasmic EZH2 and form a complex with DLC1. **a**–**c** Sections from conditional *KRAS*-G12D mouse lung tumors were immunostained with *KRAS*-G12D (green), EZH2 (red) antibodies, and DAPI (blue) for nuclei. Tumors in the conditional *KRAS*-G12D mouse lung expressed higher levels of EZH2 protein than the adjacent non-tumor lung tissue, and EZH2 (red) is present in the cytoplasm as indicated by arrows in **c**. Scale bar, 100 µm for **a**, 20 µm for **b**, 10 µm for **c**. **d** Complex formation between DLC1 and EZH2 in mouse lung tumors. Tumor extracts from conditional *KRAS*-G12D were IP with EZH2 or mock IgG antibodies followed by IB with DLC1 (top) or EZH2 (bottom) antibodies. WTE, whole tumor extract. **e**–**g** Complex formation between DLC1 and EZH2 in PDXs with mutant *EGFR* or mutant *KRAS*. Tumor extracts from PDXs with mutant *EGFR-T790M* (**e**), mutant *KRAS-G12D* (**f**), mutant *KRAS-G12C* (**g**) were IP with DLC1, or mock IgG antibodies followed by IB with EZH2 (top) or DLC1 (bottom) antibodies. WTE, whole tumor extract. Two independent experiments were performed for **a**–**g** with similar results. Source data are provided as a Source Data file.

To explore whether other NSCLC models contain cytoplasmic EZH2 and form a complex with DLC1, we evaluated these parameters in a *KRAS* mouse model and patient-derived xenografts (PDXs). Consistent with the cell line results, lung tumors in the conditional *KRAS*-G12D mouse^30^ expressed higher levels of EZH2 protein than the non-tumor lung tissue; there was cytoplasmic EZH2 (Fig. 7a–c), DLC1 protein levels were lower in the tumors than the adjacent non-tumor tissue (Supplementary Fig. 8C), and EZH2 and DLC1 formed a complex in lung tumor extracts (Fig. 7d). Similar results were seen in 3/3 PDX tumors with mutant *KRAS* (G12C and G12D) or mutant EGFR (T790M), including cytoplasmic EZH2 (Supplementary Fig. 8E, F) and EZH2-DLC1 complex formation (Fig. 7e–g). In addition, human lung adenocarcinomas and lung squamous cell carcinomas in the NCI Clinical Proteomic Tumor Analysis Consortium (CPTAC) database displayed a significant inverse correlation (*p* < 0.0001) between the overall levels of EZH2 and DLC1 protein (Supplementary Fig. 9A, B).

In breast cancer cells, p38 MAPK induced phosphorylation of EZH2-T367 (pT367) has been reported to promote its cytoplasmic localization^28^. To explore whether p38 MAPK induces the cytoplasmic localization of EZH2 in LUAD, A549 cells were treated with two p38 MAPK inhibitors, SB202190 and LY2228820. Although they reduced pT367, the inhibitors did not reduce cytoplasmic EZH2 or increase DLC1 protein (Supplementary Fig. 9C, D). Thus, cytoplasmic localization of EZH2 in A549 cells is not dependent on pT367.

### EZH2 methylates DLC1-K678

**Fig. 8:**
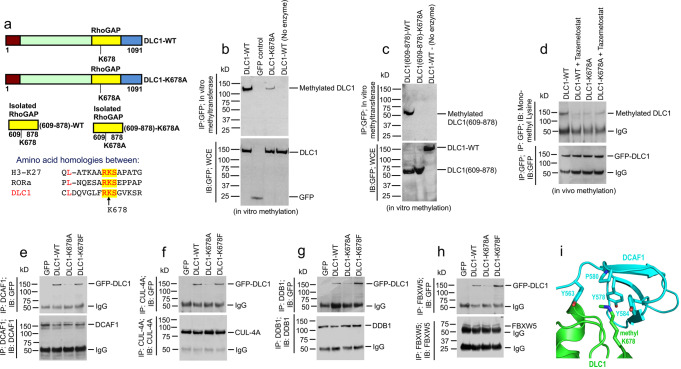
EZH2 monomethylates DLC1-K678 and monomethylated DLC1-K678 form a complex with the components of Cullin4A-RING E3 ubiquitin ligase. **a** Schematic representation of full-length DLC1 domains, isolated Rho-GAP domain, and DLC1-K678A mutant; DLC1 methylated mimetic K678F mutant is not shown. Constructs were GFP-tagged. The bottom panel emphasizes homologies between DLC1 amino acids and two known EZH2 substrates, Histone H3 and ROR-α. **b**, **c** Immunoprecipitated DLC1-WT (**b**) and isolated DLC1 Rho-GAP domain (amino acids 609-878) (**c**) were specifically methylated in vitro by recombinant EZH2. DLC1-K678A mutant was weakly methylated. GFP control and DLC1(609-878)-K678A mutant gave no signal. **d** In vivo monomethylation of DLC1-K678 was EZH2-dependent. A549 cells were transiently transfected with DLC1-WT or DLC1-K678A mutant and treated with Tazemetostat. Lysates were IP with GFP antibodies followed by IB with mono-methyl lysine (top) or GFP (bottom) antibodies. Tazemetostat treatment reduced monomethylation of DLC1-WT to that of mutant DLC1-WT and did not reduce the monomethylation signal of DLC1-K678A mutant. **e**–**h** Monomethylated DLC1-K678 forms a complex with the components of Cullin4A-RING E3 ubiquitin ligase. Monomethylated DLC1-K678 forms a complex with components of the Cull-4A ubiquitin ligase: DCAF1 (**e**), CUL-4A (**f**), DDB1 (**g**), and FBXW5 (**h**), as DLC1-WT and methylated mimetic DLC1-K678F mutant bound strongly to each subunit, while binding was much weaker with the methylation-deficient DLC1-K678A mutant. **i** Schrodinger software was used to develop a schematic model of the interaction between DLC1 and DCAF1. Based on the crystallographic structure of DLC1 and a modeling structure of DCAF1, this model suggests monomethylated K678—or the methylated mimetic K678F mutant—binds well to DCAF1 because of their favorable interaction with Y563 and Y584 of DCAF1. Two independent experiments were performed for **b**–**h** with similar results. Source data are provided as a Source Data file.

The cytoplasmic EZH2/DLC1 complex suggested EZH2 might directly methylate DLC1, which led us to compare the DLC1 amino acid sequence with two known nuclear EZH2 substrates, H3K27 and ROR-α (Fig. 8a, bottom)^31^. The identified homology suggested DLC1-K678, which is in the Rho-GAP domain, might be a substrate for EZH2. To evaluate this possibility, we mutated K678A in a cDNA encoding the full-length DLC1 protein and another encoding the Rho-GAP domain (Fig. 8a, top). Using recombinant EZH2 methyltransferase in vitro, partially purified immunoprecipitated DLC1 was strongly methylated (Fig. 8b, lanes 1 and 2); the signal in the K678A mutant was greatly reduced although not absent (Fig. 8b, lane 3). The wild-type Rho-GAP domain [DLC1(609-878)] was also strongly methylated in vitro, while the K678A mutant was not (Fig. 8c).

Methylation of DLC1 also occurs in vivo and depends on EZH2, as determined initially by transfecting A549 cells with a cDNA encoding wild-type full-length DLC1 or Rho-GAP domain, or the K678A mutant, immunoprecipitating DLC1 from cell extracts, and immunoblotting with a mono-methyl lysine or pan-methyl lysine antibody (Fig. 8d and Supplementary Fig. 9E). The methylation signal was EZH2-dependent, as Tazemetostat reduced the methylation signal of DLC1-WT to that of the DLC1-K678A mutant, which was not reduced further by Tazemetostat (Fig. 8d and Supplementary Fig. 9E). Monomethylation also occurs with endogenous DLC1, as Tazemetostat treatment of H157 cells decreased its DLC1 monomethylation signal, while MG-132 treatment increased it (Supplementary Fig. 9F). In addition, monomethylated DLC1-K678 forms a complex with the components of the CUL-4A ubiquitin ligase (DCAF1/CUL-4A/DDB1/FBXW5) and is associated with ubiquitination of K678, as DLC1-WT and the methylated mimetic K678F mutant bound strongly to each component and was strongly ubiquitinated in cells treated with the proteasome inhibitor MG-132, while the binding and ubiquitination were much weaker with the DLC1-K678A mutant (Fig. 8e–h, Supplementary Fig. 9G). Based on the crystallographic structure of DLC1 and a modeling structure of DCAF1, we have used Schrodinger software to develop a schematic model of the interaction between DLC1 and DCAF1 (Fig. 8i). Consistent with our experimental results, the model suggests monomethylated K678—or the methylated mimetic K678F mutant—binds well to DCAF1 because of their favorable interaction with Y563 and Y584 of DCAF1.

### Methylation of DLC1-K678 decreases the steady-state level of DLC1 protein

**Fig. 9:**
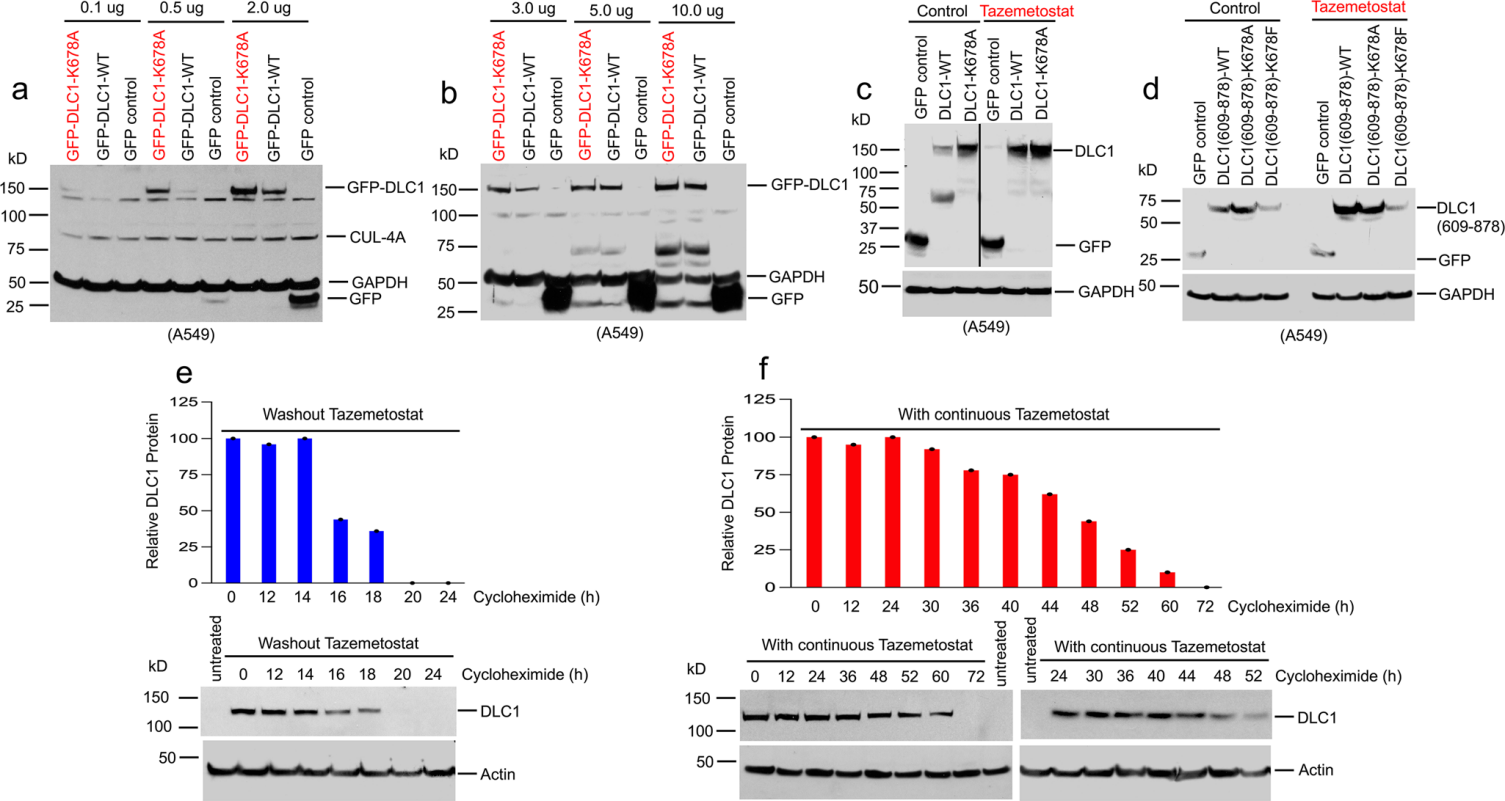
Methylation of DLC1-K678 decreases the steady-state level of DLC1 protein; EZH2 inhibition decreases DLC1 protein turnover. **a**, **b** A549 cells were transiently transfected with different amounts of cDNAs encoding GFP, GFP-tagged DLC1-WT, and GFP-tagged DLC1-K678A mutant. **a** At low amounts (0.5–2.0 μg) of cDNA, DLC1-K678A mutant protein levels were substantially higher than DLC1-WT levels. **b** At higher amounts (5.0–10 μg), the difference between DLC1-WT protein and DLC1-K678A was reduced. **c**, **d** Tazemetostat treatment increased the steady-state level of DLC1-WT and DLC1(609-878)-WT but had a marginal effect on the DLC1-K678A, DLC1(609-878)-K678A methylation-deficient mutants, or the DLC1(609-878)-K678F methylation mimetic mutant. Two independent experiments were performed for **a**–**d** with similar results. **e**, **f** Tazemetostat treatment increases DLC1 protein half-life. Cell lysates were analyzed for DLC1 and actin by western blotting, and the relative density of DLC1 was plotted against time after treatment with cycloheximide. In the absence of Tazemetostat, DLC1 protein half-life is <2 h (18 h vs. 20 h) (**e**). However, in the presence of Tazemetostat, DLC1 protein half-life is ~48 h (0 h vs. 48 h) (**f**). Graphs show the relative DLC1 protein level at the indicated time points after cycloheximide treatment. Source data are provided as a Source Data file.

If methylation of K678 leads to decreased stability of endogenous DLC1 protein, the steady-state level of transfected wild-type protein should be lower than the transfected K678A mutant, although it might be a saturable process. To evaluate these possibilities, A549 cells were transiently transfected with various amounts of cDNAs encoding GFP-tagged DLC1-WT or DLC1-K678A mutant. When low amounts of cDNA were transfected, mutant DLC1 protein levels were substantially higher than wild-type protein (Fig. 9a). However, the difference between mutant and wild-type protein became marginal with high cDNA amounts (Fig. 9a, b), implying the process responsible for decreased DLC1 protein levels is saturable. The effect was EZH2-dependent, as Tazemetostat treatment increased the steady-state level of transfected DLC1 wild-type DLC1 or Rho-GAP domain but had only a marginal effect on the methylation-deficient K678A in both constructs or the methylated mimetic K678F mutant in the Rho-GAP construct (Fig. 9c, d).

The earlier data indicated that Tazemetostat treatment increased *DLC1* mRNA expression 1.4-fold (Supplementary Fig. 1A), suggesting most of the increase in DLC1 protein was attributable to a decrease in its half-life. The lack of readily detectable DLC1 protein under standard growth conditions made it more complicated to determine the protein half-life in the absence of Tazemetostat, as cycloheximide treatment would be used to inhibit new DLC1 protein synthesis. To address this issue, we reasoned that first treating the cells with Tazemetostat and then washing it out would lead to an initial period of relatively stable DLC1 protein because of residual Tazemetostat activity, followed by a second period with a shorter half-life because of progressively lower residual Tazemetostat activity, and a third period that was essentially free of Tazemetostat activity. In the presence of Tazemetostat, cycloheximide treatment indicated the DLC1 half-life was ~48 h (Fig. 9e, f). By contrast, the half-life after the Tazemetostat washout and loss of its residual activity was no more than 2 h, about a 24-fold decrease in DLC1 protein turnover upon Tazemetostat treatment.

### An inverse relationship between *KRAS* and DLC1 that depends on EZH2

**Fig. 10:**
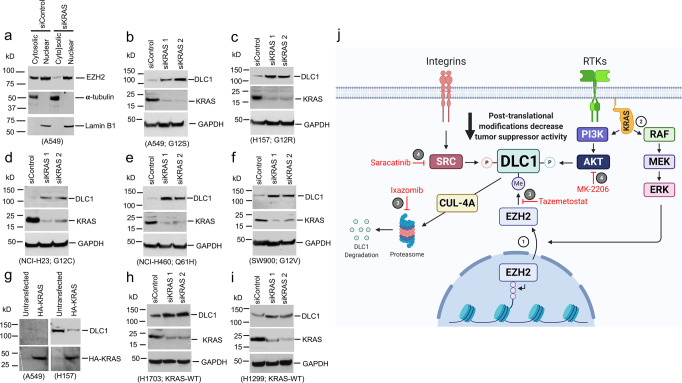
Cytoplasmic EZH2 mediates an inverse relationship between *KRAS* and DLC1 protein; and a model for regulation of DLC1 by cytoplasmic EZH2. **a** siRNA knockdown of *KRAS* drastically reduced cytoplasmic EZH2 protein in LUAD A549 cells. Cells were transfected with control siRNA or *KRAS* siRNA for 48 h, purity of nuclear and cytosolic fractions was confirmed by nuclear (Lamin-B1) and cytosolic (α-Tubulin) marker proteins. **b**–**f** siRNAs knockdown of *KRAS* increased DLC1 protein level in mutant *KRAS* lung cancer lines. Five different cell lines with mutant *KRAS* (mutant alleles indicated below the relevant panel) were transfected with two different *KRAS* siRNAs for 48 h, and the lysates were immunoblotted for KRAS and DLC1 protein. **g** Transfection of *KRAS-G12C* mutant reduced DLC1 protein in H157 cells. A549 and H157 cells were transiently transfected with HA-tagged *KRAS-G12C* mutant for 48 h, and the lysates were immunoblotted with DLC1 (top) and HA (bottom) antibodies. **h**, **i** siRNAs knockdown of *KRAS* increased DLC1 protein level in wild-type *KRAS* lung cancer lines. Two different cell lines with wild-type *KRAS* were transfected with two different *KRAS* siRNAs for 48 h, and the lysates were immunoblotted for KRAS and DLC1 protein. Two independent experiments were performed for **a**–**i** with similar results. **j** Model summarizing the main findings of this study. Mutant *KRAS* promotes the cytoplasmic localization of EZH2 protein, which is, in turn, methylates DLC1 protein, leading to CUL-4A ubiquitin-dependent proteasomal degradation of DLC1 protein. EZH2 and proteasomal inhibition prevent this degradation process, resulting in DLC1 protein stabilization. The phosphorylation of DLC1 protein by AKT and SRC kinases, which reduce the tumor suppressor activity of DLC1 protein, can be reversed by inhibitors of AKT and SRC kinases, thereby increasing the tumor suppressor activity of DLC1 protein. Source data are provided as a Source Data file.

Previous studies have indicated that mutant *KRAS* can increase EZH2^18,19^. Therefore, we evaluated the possibility of a link between wild-type or mutant *KRAS* and DLC1 protein levels. Consistent with this hypothesis, siRNAs knockdown of *KRAS* greatly reduced cytoplasmic EZH2 in A549 cells (Fig. 10a), which have mutant *KRAS*, and increased DLC1 protein in A549 and four other lung cancer lines with mutant *KRAS* (Fig. 10b–f). Conversely, transfection of mutant *KRAS* reduced DLC1 protein in LUAD H157 line, which expresses detectable levels of DLC1 protein (Fig. 10g). In addition, siRNA knockdown of *KRAS* in two lines with wild-type *KRAS* also displayed this phenotype (Fig. 10h, i).

## Discussion

The current study has identified new signaling mechanisms with pathogenetic and translational implications for cancer. The mechanisms include a key pro-oncogenic role for cytoplasmic EZH2 in the methylation and degradation of the cytoplasmic DLC1 tumor suppressor protein and an associated link between DLC1 protein levels and mutant *KRAS* signaling (Fig. 10j). The methylation of DLC1 protein induced by EZH2 is potentially reversible, which enables EZH2 inhibitors to increase the half-life of the DLC1 protein and, together with kinase inhibitors that can dephosphorylate and reactivate the tumor suppressor activity of DLC1 protein, to be used for treating tumors that express *DLC1* mRNA but lack detectable levels of DLC1 protein. This approach also extends to NSCLC lines that express readily detectable levels of steady-state DLC1 protein, as EZH2 inhibition further increases their DLC1 protein levels and regulates RhoA-GTP in a DLC1-dependent manner. In addition, treatment with a CUL-4A inhibitor or a proteasome inhibitor can substitute functionally for EZH2 inhibition both in increasing the steady-state level of DLC1 protein and in cooperating with the two kinase inhibitors for tumor treatment.

A subset of clinical LUAD biospecimens express *DLC1* mRNA but lack detectable steady-state DLC1 protein^14^. In A549 cells, which have the same phenotype, degradation of DLC1 has been attributed to the CUL-4A ubiquitin ligase and proteasome-dependent degradation^14^, a frequently activated system in LUAD and other tumor types^32^. Here, we determined that EZH2 lies upstream of DLC1 in A549 cells, DLC1 is a direct EZH2 substrate, and the process is relevant to NSCLC lines that express *DLC1* mRNA with or without readily detectable DLC1 protein. The identified relationship between EZH2 and DLC1 was unexpected, as it is attributable to a cytoplasmic EZH2 function that regulates a post-translational parameter, while the effects of EZH2 are usually attributed to its canonical nuclear methylation of H3K27 and its regulation of gene expression^16^. However, a limited number of non-canonical nuclear and cytoplasmic EZH2 methylation substrates have been previously identified^33^. They include the nuclear orphan receptor ROR-α^31^, which is degraded after its methylation, and the cytoplasmic protein talin, whose methylation can disrupt its binding to F-actin, increasing the turnover of adhesion structures^34^ and indirectly increasing VAV guanine nucleotide exchange factor activity^35^. We speculate that cytoplasmic EZH2 may methylate and regulate additional cytoplasmic targets. In breast cancer, p38 MAPK has been reported to induce phosphorylation of EZH2-T367, leading to its cytoplasmic localization^28^. However, that is a distinct phenomenon in breast cancer, as p38 inhibition neither reduced cytoplasmic EZH2 nor increased DLC1 protein in lung cancer cells.

The amino acids surrounding DLC1-K678 were found to have homology to those at H3K27 and the nuclear ROR-α site methylated by EZH2. Biochemical and genetic analyses enabled the conclusion that DLC1-K678 is a direct substrate for methylation by cytoplasmic EZH2, leading to DLC1 protein destabilization. This cytoplasmic mechanism is like that described for ROR-α, where nuclear EZH2 monomethylates ROR-α - in contrast to EZH2 trimethylation of H3K27 - leading to an interaction with DCAF1, which enables CUL-4A to ubiquitinate the methylated lysine and degrade ROR-α by the proteasome^31^. These activities are likely to be present to at least some degree in many NSCLC tumors and cell lines^17,32^, as a high proportion of lung cancers express high EZH2 levels and have active CUL-4A, and EZH2 or proteasomal inhibition increased the steady-state level of DLC1 protein in cells where DLC1 is detectable without inhibitor treatment. Beyond the cell lines, we have identified cytoplasmic EZH2 and complex formation between EZH2 and DLC1 in other lung cancer models, including NSCLC patient-derived xenografts and a mutant *KRas* mouse lung tumor model^30^.

We also determined that part of *KRAS* signaling lies upstream the EZH2-DLC1 pathway. Our observation, which is relevant to mutant and wild-type *KRAS*, builds on prior reports that mutant *KRAS* can increase EZH2 expression in a rat pancreatic cancer model^18^ and NSCLC cell lines^19^. siRNA knockdown of mutant *KRAS* was associated with a substantial reduction in cytoplasmic EZH2 in A549 cells, an increase in DLC1 protein in A549 and four other NSCLC lines with mutant *KRAS*, and two lines with wild-type *KRAS*.

These mechanistic insights were complemented by our evaluation of targeting DLC1 with rational drug combinations involving several molecular targeted agents. Our previous studies in LUAD lines that express readily detectable levels of DLC1 protein indicated AKT and SRC kinases directly phosphorylate and attenuate the Rho-GAP and tumor suppressor activities of DLC1. Inhibitors of these kinases reversed the post-translational modifications and had potent DLC1-dependent antitumor activity^7,8^. Consistent with this dependence on DLC1 protein, these kinase inhibitors had negligible effects on cellular RhoA-GTP and modest growth-inhibitory activity in cells lacking DLC1 protein. Although treatment with a proteasome inhibitor, CUL-4A activator inhibitor, or EZH2 inhibitor led to readily detectable steady-state levels of DLC1 protein, this single-agent treatment by itself had a limited impact on reducing RhoA-GTP levels or inhibiting anchorage-independent growth and tumor growth. However, combining it with AKT and/or SRC kinase inhibitors led to a cooperative decrease in RhoA-GTP, a potent reduction in anchorage-independent cell growth and the weight of xenograft tumors, and a substantial increase in markers for senescence and apoptosis. Thus, despite the considerable molecular heterogeneity between the lines, the two kinase inhibitors have potent preclinical antitumor activity in the lines, provided DLC1 protein is readily detectable in them. There was no obvious toxicity in mice, as reflected in their stable weight and normal coat appearance, even when treated with the three-drug combination of Tazemetostat, MK-2206, and Saracatinib. The degree to which side effects or toxicity may be limiting will require careful evaluation if the combination treatment advances to human clinical trials. In the mouse *KRAS* model and A549 cells, single-agent treatment with an EZH2 inhibitor was associated with inflammation^36^. It may be advantageous that our study has identified three different classes of inhibitors - EZH2, CUL-4A, and proteasome - whose antitumor activity appears to be similar when used with the kinase inhibitors, as the side effects of the inhibitors that stabilize DLC1 protein are likely to be distinct, given that their targets other than DLC1 are likely to be largely non-overlapping.

Remarkably, siRNA knockdown of DLC1 or its genetic ablation in A549 cells abolished most of the growth inhibitory activity of the three-drug combination, as determined by inhibition of anchorage-independent growth or tumor xenografts, although each inhibitor has multiple targets, strongly suggesting that DLC1 is the predominant antitumor target of the drug combination. Genetic ablation of DLC1 in H157 cells, which express readily detectable levels of DLC1 protein, showed a similar phenotype with respect to the inhibition of anchorage-independent growth by the drug combination. We speculate these observations reflect the biological importance of DLC1 tumor suppressor to the cancer process, which probably contributes to its frequent downregulation in cancer, especially lung cancer^13^, together with the ability of the inhibitors to reactivate DLC1 tumor suppressor protein by complementary, non-overlapping mechanisms.

To our knowledge, this is the first study to employ EZH2 inhibition with the goal of stabilizing a tumor suppressor protein, rather than inhibiting epigenetic changes resulting from H3K27 methylation^17,26^. EZH2 inhibition increased the half-life of DLC1 protein more than 20-fold in the A549 cells. Furthermore, DLC1 cDNA transfection confirmed the short half-life of wild-type DLC1 protein in the cells compared with the much greater stability of DLC1 carrying a point mutation of the identified methylation site. By contrast, EZH2 inhibition increased *DLC1* mRNA expression less than two-fold. This latter result is like what has been previously reported for the impact of inhibitory RNA knockdown of endogenous EZH2 on endogenous *DLC1* mRNA in liver cancer cell lines^37^; the DZNep inhibitor used in that report is a pan-histone methylase inhibitor^38^, which precludes an EZH2-specific interpretation of results obtained with it.

Our results may have implications for cancer treatment beyond the current findings. First, the observation that lung cancer cells with mutant or wild-type *KRAS* are sensitive to the three-drug treatment suggests this approach may have clinical utility against at least some tumors with mutant *KRAS*, an important need. The plausibility of this possibility is increased by the mechanistic relationship between mutant *KRAS* and low or undetectable DLC1 protein levels in several NSCLC lines that carry mutant or wild-type *KRAS*. Second, although much of this study has focused on cells that express *DLC1* mRNA but lack detectable steady-state DLC1 protein, the phenomenon of EZH2 or proteasome inhibitors increasing DLC1 protein levels was also found to occur in LUAD lines that have detectable DLC1 protein in the absence of inhibitor treatment. These results suggest that in tumors with this phenotype, the addition of an inhibitor that increases the level of DLC1 protein might augment the antitumor activity of the kinase inhibitors. Third, the approach described here, where non-genetic reactivation of a tumor suppressor is a key therapeutic target, may have relevance to other tumor types. Thus, our study provides a mechanistic rationale for combining molecular targeted agents against EZH2, CUL-4A, or the proteasome with AKT and/or SRC inhibition, which may have potential for the treatment of tumors that carry mutant *KRAS*, an important clinical need. Future preclinical studies will explore these possibilities in more detail and compare the new therapeutic approaches described here to a current standard of care therapy.

## Methods

### Plasmid constructs

GFP-tagged DLC1-WT (GFP-DLC1-WT); DLC1 GAP-dead mutant (GFP-DLC1-R718A or GFP-DLC1-R677E); GFP-tagged DLC1 Rho-GAP domain encoding residues 609-878 were constructed by PCR and subcloned into a modified pEGFP-C1 vector (Takara Bio Inc.) through Kpn1-NotI sites, as described^39^. HA and HA-tagged KRAS-G12C were obtained from Addgene. Lysine-to-Alanine (GFP-DLC1-K678A) and Lysine-to-Phenylalanine (GFP-DLC1-K678F) mutations were introduced into full-length DLC1-WT and into DLC1 fragments and DLC1 Rho-GAP domain, using a site-directed mutagenesis kit (Agilent Technologies). All PCR amplifications were confirmed by sequencing. The primer sequences were:

DLC1-K678A: GGGCTCTTCAGAGCATCGGGGGTCAAGTCC (FWD)

GGACTTGACCCCCGATGCTCTGAAGAGCCC (REV)

DLC1-K678F: GGGCTCTTCAGATTTTCGGGGGTCAAGTCC (FWD)

GGACTTGACCCCCGAAAATCTGAAGAGCCC (REV).

### Antibodies and fluorescent probes

The following antibodies were purchased from Cell Signaling Technology: AKT (4691), phospho-AKT-pS473 (4060), SRC (2108), phospho-SRC-pY416 (2101), EZH2 (5246), p38 MAPK (8690), Mono-Methyl Lysine (14679), CUL-4A (2699), DDB1 (5428), VPRBP (D5K5V; 14966), and GAPDH (2118). Two DLC1 antibodies were used: one, generated in our laboratory (DLC1 antibody; clone 428), as described previously^21^, and the other, DLC1 mouse mAb (612021, Clone 3/DLC-1 RUO), purchased from BD Biosciences. Annexin V (ab14196), β-Galactosidase (ab116), *KRAS* (ab180772), α-Tubulin (ab4074), Lamin B1 (ab65986), Pan-methyl Lysine (ab7315), NEDD8 (ab81264), DCAF1 (ab53616), GFP mouse (ab1218), and GFP rabbit (ab290), antibodies were purchased from Abcam, Inc. RhoA (ARH04) antibodies were obtained from Cytoskeleton, Inc. EZH2-Phospho-Thr367 (12868) and Phospho-EZH2 (Thr367) Polyclonal (PA5-106225) were purchased from Signalway Antibody and Thermo Fisher Scientific, respectively. Cullin 4A rabbit monoclonal antibody (JU07-33, MA5-34615) was purchased from Thermo Fisher Scientific. Ras (G12D) mouse monoclonal antibody (26036) was purchased from Vita Scientific. FBXW5 antibody (NBP3-04806) was purchased from Novus Biological, Inc. *KRAS* (WH0003845M1) and Actin (A4700) antibodies produced in mouse was purchased from Sigma Aldrich. Anti-rabbit (NA934V) and anti-mouse (NXA931V) IgG horseradish peroxidase-linked secondary antibodies were purchased from GE Healthcare. Alexa Fluor 568 anti-rabbit IgG (A10042), Alexa Fluor 488 anti-mouse IgG (A11001), and DAPI were purchased from Thermo Fisher Scientific.

### Cell lines, culture conditions, and DNA transfection

HEK 293 T and human fibroblastic H1634 cells were cultured in DMEM supplemented with 10% FBS. NSCLC lines H1703, H157, A549, and H358 were provided by Dr. C. Harris, National Cancer Institute, Bethesda, MD, which were originally obtained from ATCC. NCI-H23, NCI-H460, SW900 were procured from ATCC. All cancer cell lines were cultured in RPMI-1640 supplemented with 10% FBS. Transient transfections were performed with Lipofectamine 3000 (Life Technologies) and cultured for 48 h. Stable clones expressing GFP or DLC1 mutants were made by transfecting A549, H1703, or H358 cells with Lipofectamine 3000, followed by G418 selection (0.9 µg/ml).

### siRNA transfection, DLC1 gene knockout, and treatment of cells with inhibitors

To suppress expression of specific mRNAs, cells were transfected with 160 nM of siRNAs for *DLC1*, *EZH2*, *KRAS,* or with scrambled control siRNAs, and harvested 48 h later. Suppression of protein expression, at least with two different siRNAs, was confirmed by immunoblotting. Validated siRNAs for human DLC1 (Hs_DLC1 siRNA_5, SI03219909, and Hs_DLC1 siRNA_11, SI04952213) were from QIAGEN, as were negative control siRNAs (control siRNA 1, 1027280; and control siRNA 2, 1027310). The sequence for each DLC1 siRNA was as follows: Hs_DLC1 siRNA_5 sense sequence: 5′-CGAUGUCGUAAUUCCUAUATT-3′; Hs_DLC1_5 antisense sequence: 3′-CGGCUACAGCAUUAAGGAUAU-5′; Hs_DLC1_11 sense sequence: 5′-GGAGUGUAGGAAUUGACUATT-3′; Hs_DLC1_11 antisense sequence: 3′-gaCCUCACAUCCUUAACUGAU-5′. ON-TARGET plus Human KRAS (3845) siRNA, Smart pool KRAS siRNA, ON-TARGET plus Human EZH2 (2146) siRNA and Smart pool EZH2 siRNA were from Dharmacon, Inc. The sequences for siRNAs for *KRAS* and *EZH2* were:

Human KRAS-siRNA J-005069-08; GGAGGGCUUUCUUUGUGUA

Human KRAS-siRNA J-005069-09; UCAAAGACAAAGUGUGUAA

Human KRAS-siRNA J-005069-10; GAAGUUAUGGAAUUCCUUU

Human KRAS-siRNA J-005069-11; GAGAUAACACGAUGCGUAU

Human EZH2-siRNA J-004218-06; GAGGACGGCUUCCCAAUAA

Human EZH2-siRNA J-004218-07; GCUGAAGCCUCAAUGUUUA

Human EZH2-siRNA J-004218-08; UAACGGUGAUCACAGGAUA

Human EZH2-siRNA; J-004218-09; GCAAAUUCUCGGUGUCAAA

For CRISPR/Cas9 mediated knockout of the DLC1 gene in A549 and H157 LUAD lines, cells were transfected with two different constructs (pAG0266 and pAG0267) with single-guide RNA (sgRNA) for DLC1 gene. Lenti-SpCas9-2A-GFP_DLC1-IVT was used to deliver individual sgRNA. The sequences for both guide RNAs are targeting DLC1 exon 5 of variant 2. Sequences of gRNA primers for DLC1 and non-targeted control gRNA were:

DLC1-gRNA1: AGTGCGTGCAACAAGCGGGT (pAG0266)

DLC1-gRNA2: TGATGACGGAGTTAGTCCGG (pAG0267)

Non-targeted control-gRNA: GTGTCGTGATGCGTAGACGG (pAG0224)

Lipofectamine 3000 (Life Technologies) was used to transfect plasmid DNA according to the manufacturer’s instructions. GFP-positive single cell was sorted by FACS Aria UV into a sterile 96-well culture plate, which helped us to derive single-cell clones of DLC1 gene knockout.

AKT inhibitors (MK-2206 and Perifosine) and SRC inhibitors (Saracatinib and Bosutinib), p38 MAPK inhibitor (SB202190; FHPI) (used at 10 µM each) were purchased from Selleck Chemicals. We procured MAPK inhibitor Ralimetinib dimesylate (LY2228820 dimesylate; HY-13241) from Med Chem Express, NAE Inhibitor MLN4924 (CAS 951950-33-7) from Calbiochem, and MG-132 (M7449) and Cycloheximide (C4859) from Sigma Aldrich. Inhibitor for EZH2 (Tazemetostat) and proteasome (Ixazomib) and all other pharmacological compounds (used at 10 µM each) were provided by Dr. James H. Doroshow, National Cancer Institute, Division of Cancer Treatment and Diagnosis (DCTD), Frederick, MD.

### PDX models

The PDX models (LG0807-F1297 [Lot# MM1516; *EGFR*-T790M], K00052-001-T [Lot# CM0240; *KRAS*-G12D], LG0567-F567 [Lot# CK2648; *KRAS*-G12C], K12877-001-R [*KRAS*-wild-type]) used in this study were originally developed by The Jackson Laboratory, Bar Harbor, ME (Jackson IDs TM00204, TM00302, TM00192, and J000093018, respectively). The PDX model 941728-121-R (Lot# JS0923; *KRAS*-G12C) was developed by the NCI Patient-Derived Models Repository (NCI PDMR; Frederick, MD; https://pdmr.cancer.gov).

### RT- PCR for detecting endogenous DLC1 expression

RNA was isolated from the cells using RNeasy Mini Kit (Qiagen), according to the manufacturer’s protocol. RNA was reverse transcribed into cDNA using random primers and other reagents from the High-Capacity cDNA Reverse Transcription Kit (Life Technologies). The human DLC1 transcript was detected with forward (5'-CACAGGACAACCGTTGCCTCAG) and reverse (5'-CTCTTCAGGGTGTTGAGATGGA) primers that amplify a 465 bp product (nt 2256-2720 of NM_006094). PCR was performed using Taq DNA polymerase (New England Biolabs), with an initial denaturation of 94 °C for 3 m, followed by 35 cycles of 40 sec at 94 °C, 40 sec at 55 °C, 1 min at 72 °C, and a final extension of 4 min at 72 °C. As a control, amplifications were performed with human GAPDH forward (5'-GACATCAAGAAGGTGGTGAAGC) and reverse (5'-GATGGTACATGACAAGGTGCGG) primers, which yield a 417 bp product (nt 845-1261 of NM_002046). PCR products were analyzed by agarose gel electrophoresis and ethidium bromide staining.

### RNA extraction and Quantitative RT-PCR

RNA was purified using RNeasy Mini kit (Qiagen), according to the manufacturer’s instructions. RNA extraction from cells treated without or with the indicated inhibitors or siRNAs were purified. Precipitated RNA was eluted in nuclease-free water and quantified using a Nanodrop. Complementary DNA was synthesized from 500 ng RNA using random primers (QuantiTect II Reverse Transcription Kit; Qiagen) and High-Capacity Reverse Transcription Kit (Life Technologies) and qRT-PCR was performed with Power SYBR Green PCR Master Mix (Life Technologies) using specific oligonucleotide primers in an ABI 7900HT thermocycler (Applied Biosystems, Foster City, CA). Primers directed at human DLC1 and GAPDH sequences were designed using Primer3 or from existing literature and synthesized by Integrated DNA technologies (Skokie, IL).

Primers used in quantitative PCR were as follows:

DLC1 forward: CGTTGTTTTAAGAACCTGGAGGA

DLC1 reverse: ATTAACCCTCACACCCACCA

GAPDH forward: ACAGTCAGCCGCATCTTCTT

GAPDH reverse: ACGACCAAATCCGTTGACTC

Fold changes were calculated using the 2^-ddCt method. The calculated threshold values were determined by the maximum curvature and Ct was calculated as Ct^Control^ − Ct^sample^. All qRT-PCR values were normalized with GAPDH.

### Nuclear and cytosolic fractionation

Nuclear and cytosolic fractions of cells were purified using a nuclear/cytosolic fractionation kit (Cell Biolabs, Inc.), as per the manufacturer’s instructions. Briefly, cells were collected by centrifugation for 5 m at 4 °C (600 × g), washed with ice cold PBS, and gently resuspended the cell pellets with 500 μL of ice cold, 1X cytosol extraction buffer containing DTT, and protease inhibitors. Next, we transfered the suspension into a prechilled microcentrifuge tube and incubated on ice for 10 m, and then 25 μL of cell lysis reagent was added, vortex for 10 s and centrifuge for 10 m at 4 °C (800 × g). Resulting supernatant (cytoplasmic fraction) was transfered to a clean, chilled microcentrifuge tube and stored at −80 °C until use. For nuclear protein extraction, the pellet was gently resuspend in 100 μL of ice cold, 1X nuclear extraction buffer containing DTT and protease inhibitors by pipetting up and down, maintained on ice for 30 m, vortexing for 10 s, and then centrifuged for 30 m at 4 °C (14000 × g). The supernatant (nuclear protein extract) was stored at −80 °C until use. All buffers were supplemented with protease cocktail and phosphatase inhibitors.

### Co-immunoprecipitation (co-IP) and immunoblotting (IB)

Co-IP and IB were performed according to the protocol as described^40^. For co-IP experiments, equal amounts of protein from each cell lysate were precleared with protein G slurry (Millipore Corp.) and then incubated with the indicated antibodies or control IgG for 1 h at RT. After incubation, 30 µl of protein G slurry was added to each immune reaction and rotated at 4 °C overnight. The immunopellets were washed three times as above. Co-IP proteins were eluted by boiling for 5 min in 30 µl Laemmli sample buffer containing 5% (vol/vol) 2-mercaptoethanol. Eluted proteins were resolved on a NuPAGE 4–12% BisTris gel and detected by IB using specific antibodies (1:1000 dilution). Immunoreactive bands were detected by enhanced chemiluminescence (ECL-Plus; GE Healthcare) using horseradish peroxidase-linked anti-rabbit or anti-mouse secondary antibodies (1:5000 dilution).

### EZH2 methyltransferase assays

Lysates from transfected cells were IP with GFP antibody, and immunopellets were sequentially washed once with high-salt HNTG buffer (20 mM HEPES, 500 mM NaCl, 0.1% Triton X-100, and 10% glycerol), twice with low-salt HNTG buffer (20 mM HEPES, 150 mM NaCl, 0.1% Triton X-100, and 10% glycerol), and once with methylation reaction buffer (50 mM Tris, pH 8.0, 10 mM MgCl_2_, 1 mM EGTA, and 0.5 mM DTT). The methylation reaction was performed in 30 µl of reaction buffer containing 15 μci of S-adenosyl-I-[methyl-3H] methionine ([3H]-SAM) or cold SAM as the methyl donor, and 100 ng of recombinant active EZH2-PRC2 complex (Active Motif, Cat. # 31387) at 30 °C for 1 h. The reaction was stopped by adding 10 µl of 4 × Laemmli sample buffer and heating at 95 °C for 5 min. Proteins were separated by SDS-PAGE electrophoresis, autoradiographed or immunoblotted with mono-methyl Lysine or Pan-methyl Lysine antibodies.

### Protein half-life measurement

A549 cells were pre-treated with or without Tazemetostat for 24 h to stabilize the DLC1 protein. Subsequently, 10 μM cycloheximide was added, as described^41^ to inhibit protein synthesis in the absence (washout with PBS) or in the continuous presence of fresh Tazemetostat. Cells were collected at the indicated time points following treatment with cycloheximide and then subjected these cell lysates to western blotting for DLC1 and actin antibodies. DLC1 protein half-life was calculated from decay rates in DLC1 protein levels, which were determined by the relative DLC1 band intensity, then by normalization with the actin band intensity.

### Immunofluorescent staining

Transiently or stably transfected cells were seeded onto glass chambers, incubated for 24 h, and fixed with 4% paraformaldehyde for 20 min. Fixed cells or deparaffinized tissues sections were permeabilized with 0.25% Triton X-100 in PBS and then blocked with 3% BSA in PBS for 2 h. The cells or tissues sections were incubated with a 1:200 dilution of the indicated primary antibodies at 4 °C overnight. After being thoroughly washed in PBS, cells were incubated with the appropriate 1:250 Alexa Fluor-conjugated secondary antibodies for 1 h. To visualize nuclei, cells were incubated with DAPI (1:2,500) for 1 h. After staining, cells were thoroughly washed with PBS and mounted with gel mounting solution (Biomeda).

### Fluorescent confocal microscopy

Confocal microscopy of fluorescent-labeled cells was performed using a microscope (LSM 780; Carl Zeiss) with an excitation wavelength of 488 nm to detect transfected GFP fusion proteins. Alexa Fluor probes were viewed with excitation wavelengths of 488 nm (Alexa Fluor 488) and 568 nm (Alexa Fluor 568). Images were made at RT using photomultiplier tubes with a Plan-Apochromat 63×/1.4 NA oil differential interference contrast objective lens with a 2× magnifier to produce a 125× magnification. The colocalization of two proteins was analyzed by confocal software (ZEN 2012; Carl Zeiss). For quantification of representative morphology in each group, ∼30 cells per condition randomly selected from several fields were analyzed. The images were minimally processed for levels/contrast adjustment in DAPI panels, and the adjustment was done for all images using Adobe Photoshop CC software. The adjustments do not enhance, erase, or misrepresent any information present in the original images.

### RhoA-GTP (Rhotekin-RBD pull-down) assay

A Rho activation assay kit (EMD Millipore) was used to measure GTP-bound RhoA, as described^40^. In brief, equal amounts (1000 µg) of each cell lysate were incubated with 30 µg GST-Rhotekin Rho-binding domain coupled to glutathione-agarose beads for 45 min. Beads were washed three times with washing buffer. Washed samples were subjected to 4–12% SDS-PAGE, transferred onto nitrocellulose membranes, and detected by IB, using RhoA antibody (ARH04 from Cytoskeleton; and 05-778, clone 55, from EMD Millipore).

### Anchorage-independent growth assay

For soft agar assays, a 0.6% agar (BD) base in RPMI-1640 medium was placed in 60-mm dishes for 1 h at RT. 1.0 × 10^5^ cells were mixed with a complete medium containing 0.4% agar and placed over 0.6% basal agar in 60 mm dishes. Cells were grown for 3 weeks and were continuously treated without or with Tazemetostat, MK-2206, and Saracatinib, and colonies were photographed microscopically and quantified with a colony counter.

### In vivo tumorigenesis and treatment of mice with inhibitors

The mouse studies were approved by the National Cancer Institute Animal Care and Use Committee and conducted in compliance with the approved protocols. NOD.SCID/NCR mice were obtained from Charles River Laboratory, National Cancer Institute centralized animal order system. We used 6–8 weeks old mice for this study. The animals were housed under standard laboratory conditions in 12 h dark/light cycle (6 am–6 pm) at ambient temperature 68–76 F with 30–70% humidity and were provided continuous food and water supply. For the treatment of mice with xenograft tumors, A549 cells were trypsinized, washed with cold PBS, diluted to 10^7^ cells/ml with serum-free medium/Matrigel basement membrane matrix (BD Biosciences) at a ratio of 3:1, and injected subcutaneously into NOD-SCID mice (10^6^ cells/injection). When tumors were ~0.5 cm in diameter, mice were randomly divided into groups and were treated daily with EZH2 inhibitor Tazemetostat (50 mg/kg) or proteasomal inhibitor Ixazomib (15 mg/kg) for 1 week followed by the treatment with a combination of Tazemetostat (50 mg/kg) or Ixazomib (15 mg/kg) together with SRC inhibitor Saracatinib (50 mg/kg), AKT inhibitor MK-2206 (50 mg/kg), all three-drug in combination or vehicle control for two more weeks, and the remaining tumor tissues were then excised, weighed, and processed for biochemical assays after treatment.

### Bioinformatics analyses of CPTAC proteomics data for EZH2 and DLC1 protein

For EZH2 and DLC1 correlation analysis, the protein abundance values (log 2) of lung adenocarcinoma and lung squamous cell carcinoma were downloaded from Clinical Proteomic Tumor Analysis Consortium (CPTAC) data portal (https://proteomics.cancer.gov/data-portal). The correlation was plotted, and the Pearson correlation coefficient (r) was measured using statistical computing and graphic software (version 4.1.0). The *p*-value < 0.05 was considered statistically significant.

### Data analysis, statistics, and reproducibility

The Nuclear and cytoplasmic distribution of proteins were analyzed by Imaris cell imaging software, version 9.7.2. At least two independent experiments were performed for all in vitro experiments. Immunoblots were quantified by densitometric scanning using ImageJ software 1.53a. Results in bar graphs are displayed as mean ± standard deviation (SD) from two or three experiments. All experiments were designed with matched control conditions within each experiment. Data distribution was assumed to be normal, but this was not formally tested. For the statistical analysis, a parametric unpaired *t*-test with Welch’s correction was performed using Prism software (version 9.1.2 (225); GraphPad). The statistical test was two-sided, and no adjustments were made for multiple comparisons. *p* < 0.05 was considered statistically significant.

### Reporting summary

Further information on research design is available in the Nature Research Reporting Summary linked to this article.

## Supplementary information

Supplementary Information

Reporting Summary

## Data Availability

Clinical Proteomic Tumor Analysis Consortium (CPTAC) data portal is downloaded from (https://proteomics.cancer.gov/data-portal). All the other data are available within the article and its Supplementary Information. Source data are provided with this paper.

## Source data

Source Data
