# Supplementary material for: Inhibition of cytoplasmic EZH2 induces antitumor activity through stabilization of the DLC1 tumor suppressor protein
Source: Nat Commun. 2021 Dec 3;12:6941. doi: 10.1038/s41467-021-26993-3 (PMC8642553; doi:10.1038/s41467-021-26993-3)
Supplement: Supplementary file 1 — Supplementary Information [file 41467_2021_26993_MOESM1_ESM.pdf]

SUPPLEMENTARY FIGURES AND LEGENDS

Inhibition of cytoplasmic EZH2 induces antitumor activity through stabilization of the DLC1 tumor suppressor protein

Tripathi et al., Nature Communications 2021

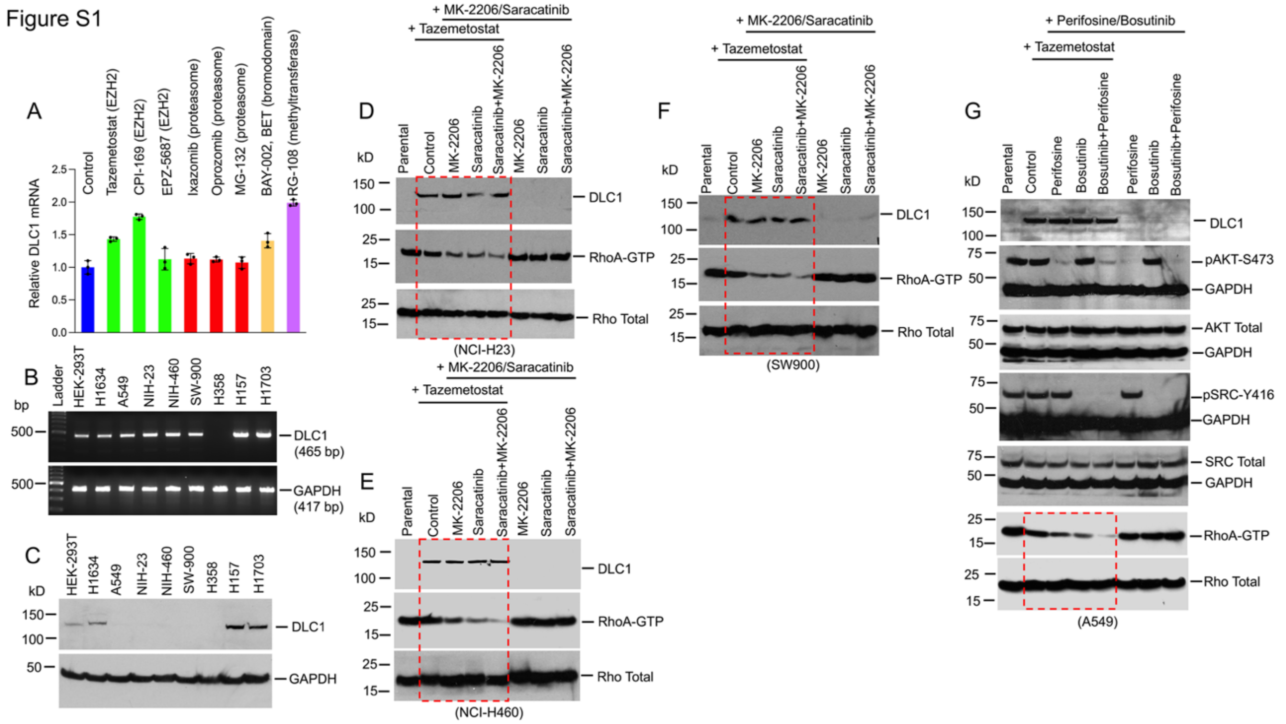

Supplementary Fig. 1. Quantification of *DLC1* mRNA expression in the absence or presence of the indicated inhibitors; Tazemetostat increases the steady-state level of DLC1 protein, whose Rho-GAP activity is increased by AKT and SRC inhibitors. (A) Relative levels of *DLC1 mRNA* in A549 LUAD cells in response to treatment with the indicated inhibitors for 48 hours. Graph shows mean ± SD within each group. Error bars represent standard deviation (SD). N=3 independent replicates. (B-C) Expression of *DLC1 mRNA* (B) and DLC1 protein (C) in the indicated lines used in the study. GAPDH was used as a loading control. (D-F) Tazemetostat treatment stabilized the DLC1 protein in *KRAS* mutant LUAD lines NCI-H23 (D), NCI-H460 (E), and lung squamous cell carcinoma SW900 (F). MK-2206 or Saracatinib decreased RhoA-GTP in Tazemetostat treated cells, which have readily detectable DLC1 protein, but not in parental DLC1 protein-negative cells. Combined treatment of MK-2206 and Saracatinib with Tazemetostat enabled further reduction in RhoA-GTP in all lines examined. (G) Tazemetostat treatment increased DLC1 protein in A549 cells. AKT inhibitor Perifosine or SRC inhibitor Bosutinib decreased RhoA-GTP in Tazemetostat treated cells, but not in parental DLC1 protein-negative cells. Combined treatment of Perifosine and Bosutinib with Tazemetostat enabled further reduction in RhoA-GTP, similar what was observed in Fig. 1 with MK-2206 and Saracatinib, two other AKT and SRC kinase inhibitors, respectively. Two independent experiments were performed for each B-G with similar results. Source data are provided as a Source Data file.

Figure S2

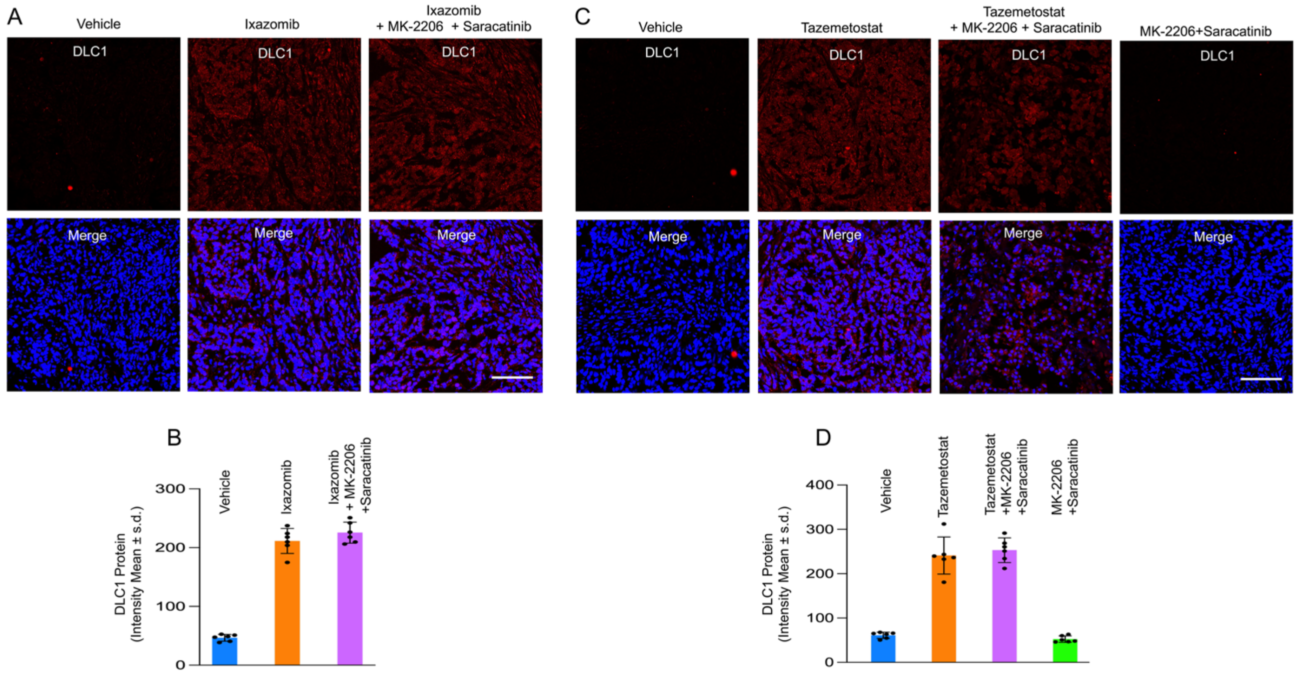

Supplementary Fig. 2. Ixazomib and Tazemetostat treatment induce DLC1 protein expression in xenograft tumors. (A-B) Sections from Ixazomib treated tumors shown in Fig. 2c induced DLC1 protein expression. Tumor tissues sections were immunostained with DLC1 (red) antibodies and DAPI (blue) for nuclei. Tumor treated with Ixazomib alone or in combination of MK-2206, and Saracatinib expressed higher levels of DLC1 protein than the vehicle control tumors. Scale bars, 100 µm. (B) Quantification, in arbitrary units, of DLC1 mean intensity ± SD, as shown in A. Error bars represent SD. N=6 tumor sections per group. (C-D) Sections from Tazemetostat treated tumors shown in Fig. 3c induced DLC1 protein expression. Tumors treated with Tazemetostat alone or in combination of MK-2206 and Saracatinib expressed higher levels of DLC1 protein than the vehicle control tumors, while the combination of MK-2206 and Saracatinib did not show a DLC1 protein signal. Scale bars, 100 µm. (D) Quantification of the DLC1 mean intensity ± SD, as shown in C. Error bars represent SD. N=6 tumor sections per group. Source data are provided as a Source Data file.

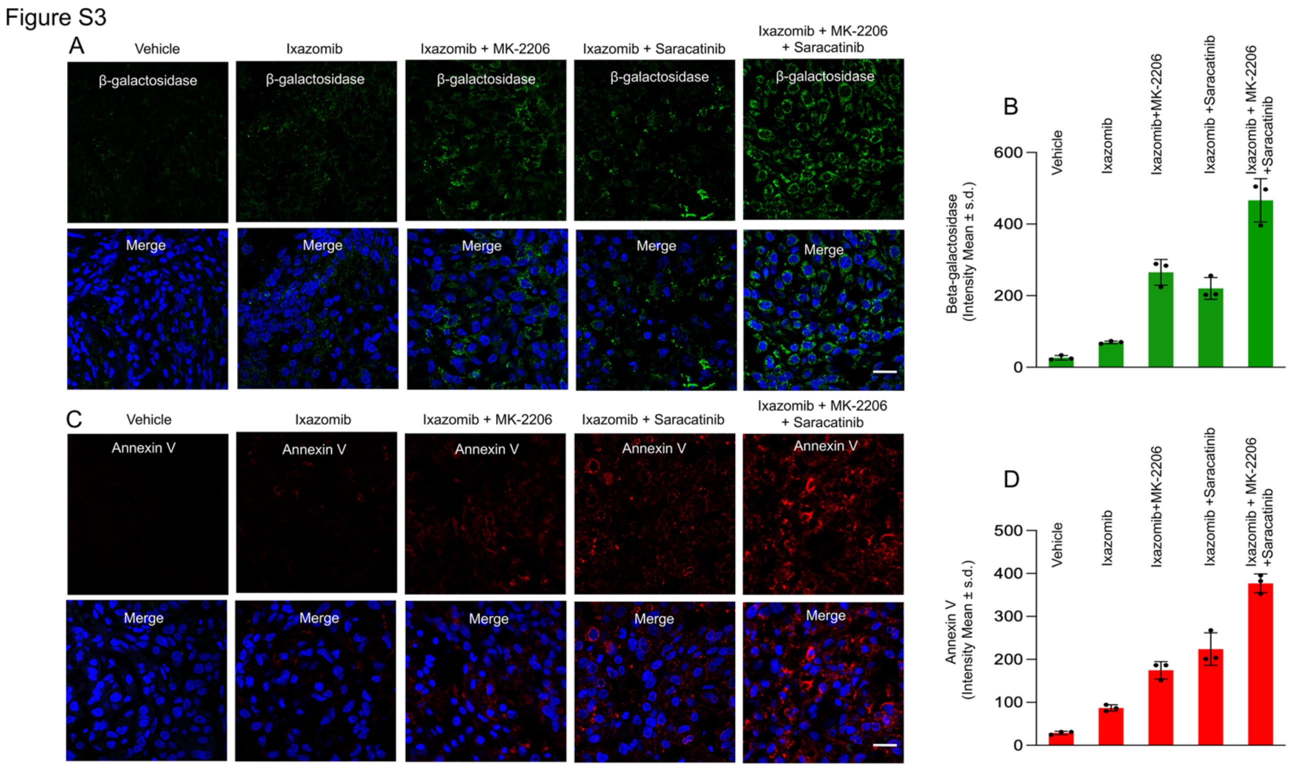

Supplementary Fig. 3. Saracatinib and MK-2206 treatment induce markers of cellular senescence and apoptosis in xenograft tumors treated with Ixazomib together with MK-2206 and/or Saracatinib. (A-B) Ixazomib treated tumors shown in Fig. 2c, which were also treated with MK-2206 and/or Saracatinib, induced cellular senescence, as measured by β-galactosidase, and (C-D) apoptosis, as measured by annexin V. Scale bars, 20 µm. The combined treatment with all three inhibitors, Ixazomib, MK-2206, and Saracatinib, led to the highest signals for both β-galactosidase and annexin V. (B) Quantification, in arbitrary units, of the β-galactosidase mean intensity ± SD, as shown in A. Error bars represent SD. N=3 tumor sections per group. (D) Quantification of the annexin V mean intensity ± SD, as shown in C. Error bars represent SD. N=3 tumor sections per group. Source data are provided as a Source Data file.

Figure S4

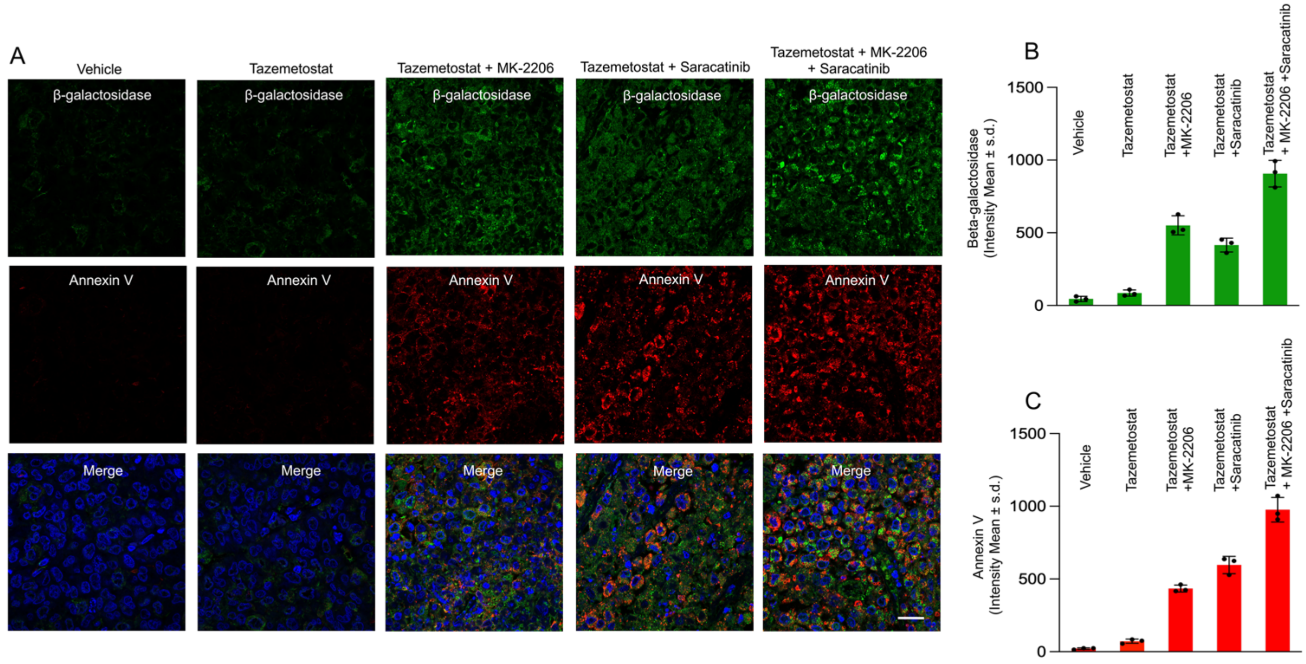

Supplementary Fig. 4. Saracatinib and MK-2206 treatment induce markers of cellular senescence and apoptosis in tumor xenografts treated with Tazemetostat together with MK-2206 and/or Saracatinib. (A) Tazemetostat treated tumors shown in Fig. 3c, which were also treated with MK-2206 and/or Saracatinib, induced cellular senescence, as measured by β-galactosidase (green), and apoptosis, as measured by annexin V (red). Scale bars, 20 µm. The combined treatment with all three inhibitors Tazemetostat, Saracatinib and MK-2206, led to the highest signals for both β-galactosidase and annexin V. (B-C) Quantification of the β-galactosidase mean intensity ± SD is shown in B, and annexin V mean intensity ± SD is shown in C. Error bars represent SD. N=3 tumor sections per group for both B and C. Source data are provided as a Source Data file.

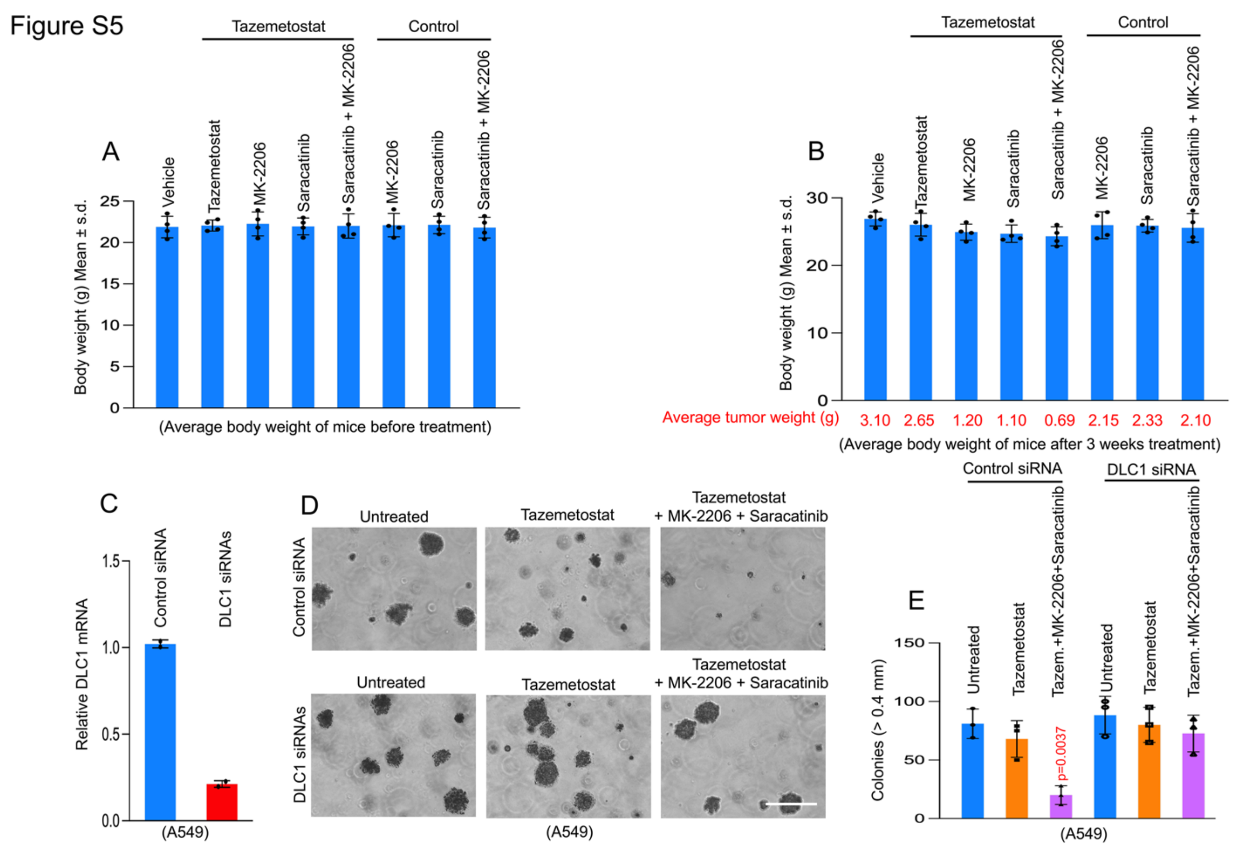

Supplementary Fig. 5. Treatment with a three-drug combination is not associated with mouse weight loss; DLC1 is a critical target of a three-drug combination. (A-B) Graphs show average body weight (g) of mice before treatment (A) and after three weeks treatment (B) in all groups. For each group, mean and are shown. Error bars represent SD. The numbers at the bottom of Graph B represent the average tumor weight (g). Much of the change in weight is attributable to the weight of the tumor. N=4 mice per group for both A and B. (C-E) Decreased DLC1 expression abrogates most of the anchorage-independent growth inhibition of A549 cells treated with Tazemetostat, MK-2206 and Saracatinib for 3 weeks. (C) DLC1 siRNA efficiently suppressed the *DLC1 mRNA* expression in A549 LUAD cells. N=2 independent replicates. For each group, mean and SD are shown. Error bars represent SD. (D) Colony growth with DLC1-expressed or DLC1-knockdown A549 cells. Bar, 2 mm. (E) Quantification of agar colonies in D. N=3 independent experiments. Error bars represent SD. For the statistical analysis, parametric unpaired t test with Welch's correction was performed using Prism software. The statistical test was two-sided, and no adjustments were made for multiple comparisons. p=0.0037 for untreated versus Tazemetostat + MK-2206 + Saracatinib treatment in DLC1-positive A549 cells. Source data are provided as a Source Data file.

Figure S6

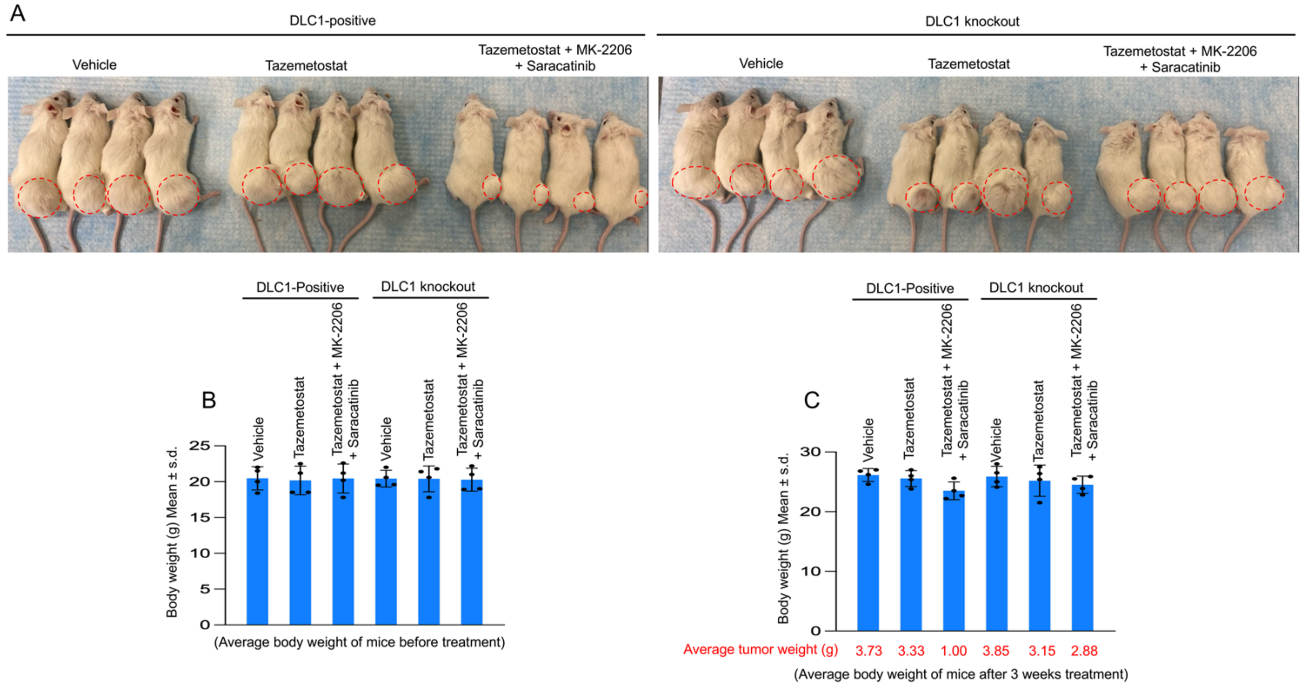

Supplementary Fig. 6. Treatment with the combination of three inhibitors is not associated with changes in mouse coat appearance or weight loss. (A) Treatment of tumor bearing mice with three-drug combination did not show visible side effects, such as change in coat appearance or weight loss unrelated to a decrease in tumor weight. N=4 mice per group. (B-C) Graphs show average body weight (g) of mice before treatment (B) and after three weeks treatment (C) in all groups. For each group, average mouse weight (g) and SD are shown. Error bars represent SD. The numbers at the bottom of panel C represents the average tumor weight (g). N=4 mice per group for both B and C. Source data are provided as a Source Data file.

Figure S7

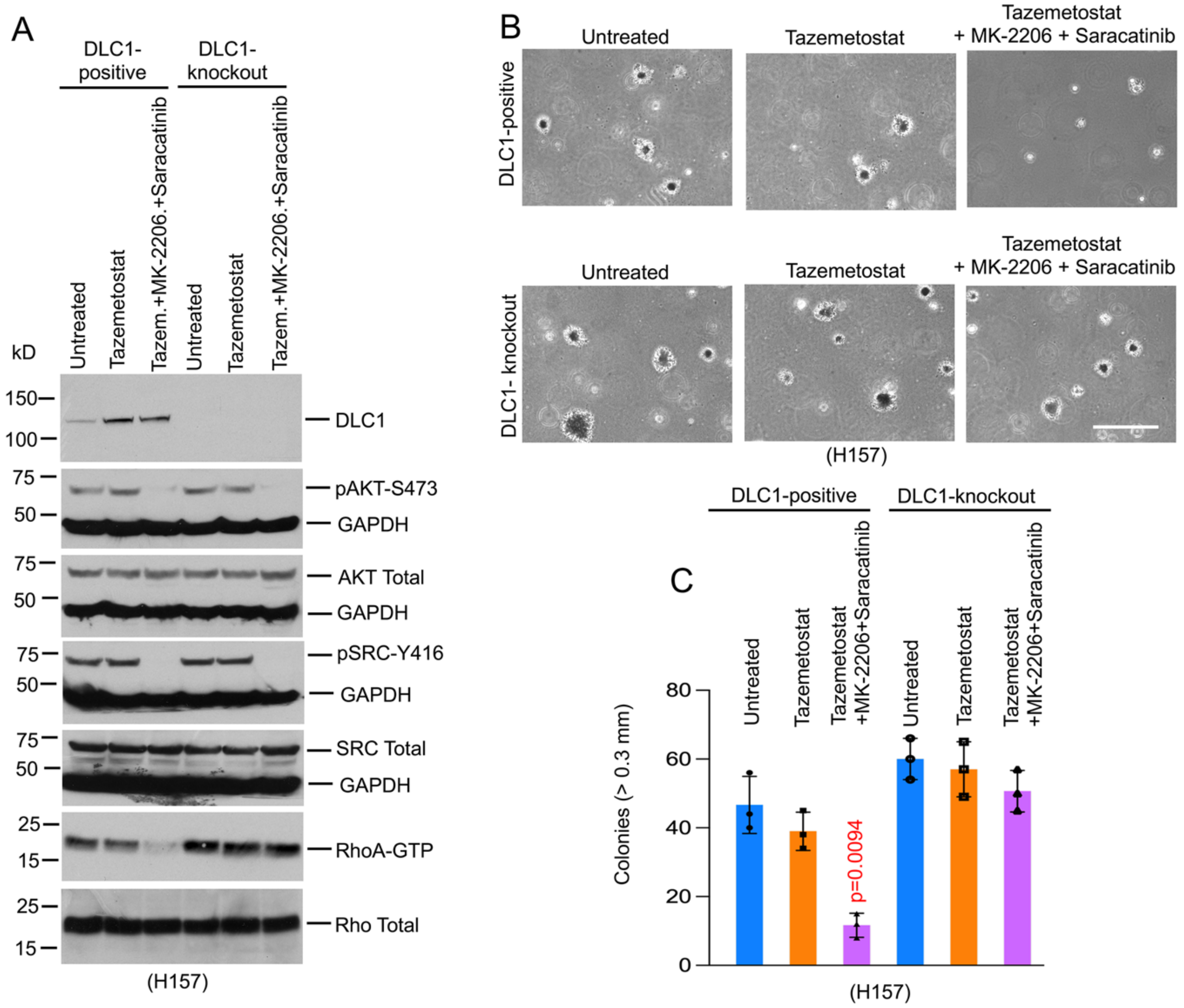

Supplementary Fig. 7. Endogenous DLC1 is an important target of inhibitors combination. (A) Steady-state DLC1 protein levels in H157 LUAD cells after Tazemetostat treatment, which increases DLC1 protein in DLC1-positive H157 cells, but not in the isogenic DLC1-knockout cells. Combined treatment of MK-2206 and Saracatinib decreases RhoA-GTP in DLC1-positive cells, but not in the isogenic DLC1-knockout cells, although MK-2206 and Saracatinib inhibit AKT activity (measured by pAKT-S473) and SRC activity (measured by pSRC-Y416) in all treated samples. Two independent experiments were performed with similar results. (B-C) Knockout of DLC1 abrogates most of the anchorage-independent growth inhibition of H157 cells treated with Tazemetostat, MK-2206, and Saracatinib for 3 weeks. (B) Colony growth with DLC1-positive or DLC1-knockout H157 cells. Bar, 2 mm. (C) Quantification of agar colonies in B. For each group, mean and SD are shown. Error bars represent SD. N=3 independent experiments. For the statistical analysis, parametric unpaired t test with Welch's correction was performed using Prism software. The statistical test was two-sided, and no adjustments were made for multiple comparisons. p=0.0094 for untreated versus Tazemetostat + MK-2206 + Saracatinib treatment in DLC1-positive H157 cells. Source data are provided as a Source Data file.

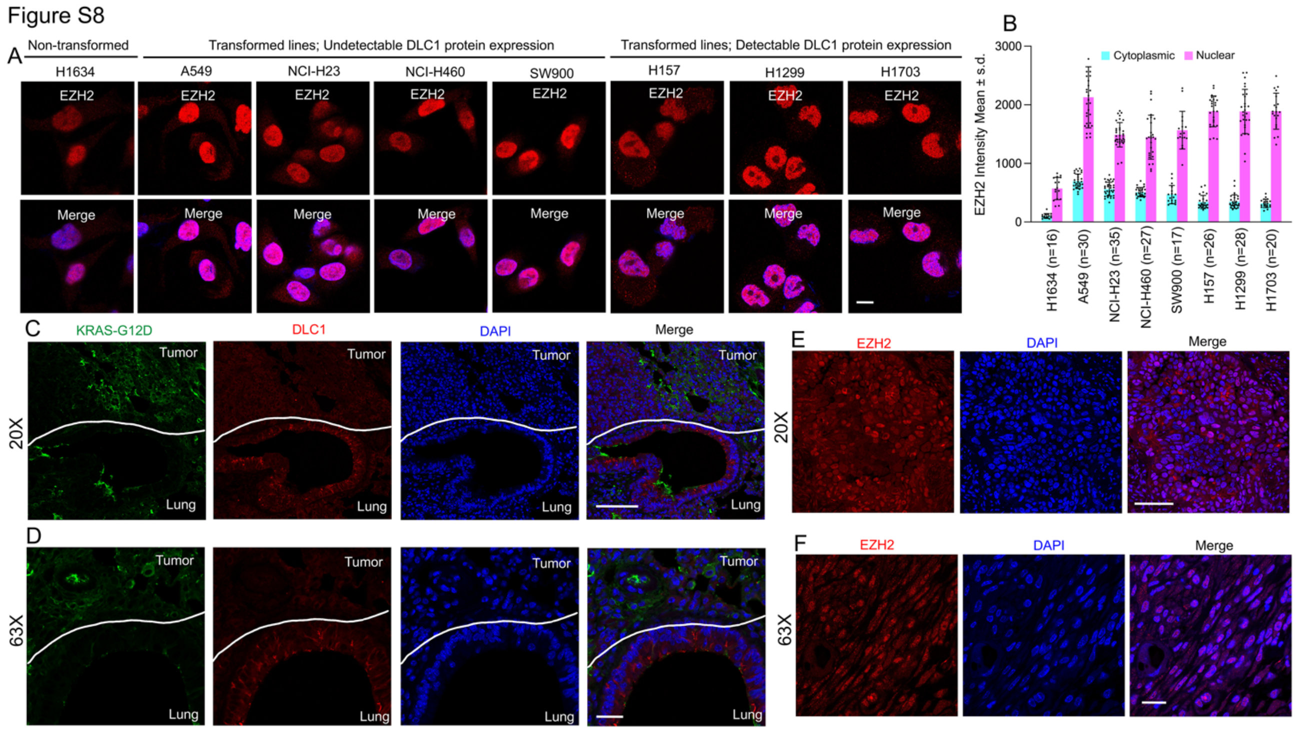

Supplementary Fig. 8. NSCLC PDXs and several cancer lines contain cytoplasmic EZH2. (A-B) A549 LUAD and several other lung cancer lines have more nuclear and cytoplasmic EZH2 protein than non-transformed H1634 cells. Cells were stained with EZH2 antibodies (red) and DAPI (blue) for nuclei. Scale bar, 10 µm. (B) Mean cytoplasmic and nuclear EZH2 level ± SD was calculated from at least 16 cells randomly selected from several fields of two independent experiments. Error bars represent SD. (C-D) Sections from conditional KRas-G12D lung tumors were immunostained with KRas-G12D (green) and DLC1 (red) antibodies, and DAPI (blue) for nuclei. Lung tumors in the conditional KRas-G12D mouse lung expressed lower levels of DLC1 protein than the adjacent non-tumor lung tissue. Scale bar, 100 µm for C and 20 µm for D. (E-F) Sections from a G12C mutant *KRAS* PDX tumor (LG0567-F567) were immunostained with EZH2 (red) antibodies and DAPI (blue) for nuclei. PDX tumor harboring mutant *KRAS* G12C expressed substantial amount of EZH2 protein and some EZH2 (red) is present in the cytoplasm. Scale bar, 100 µm for E and 20 µm for F. Two independent experiments were performed for C-F with similar results. Source data are provided as a Source Data file.

Figure S9

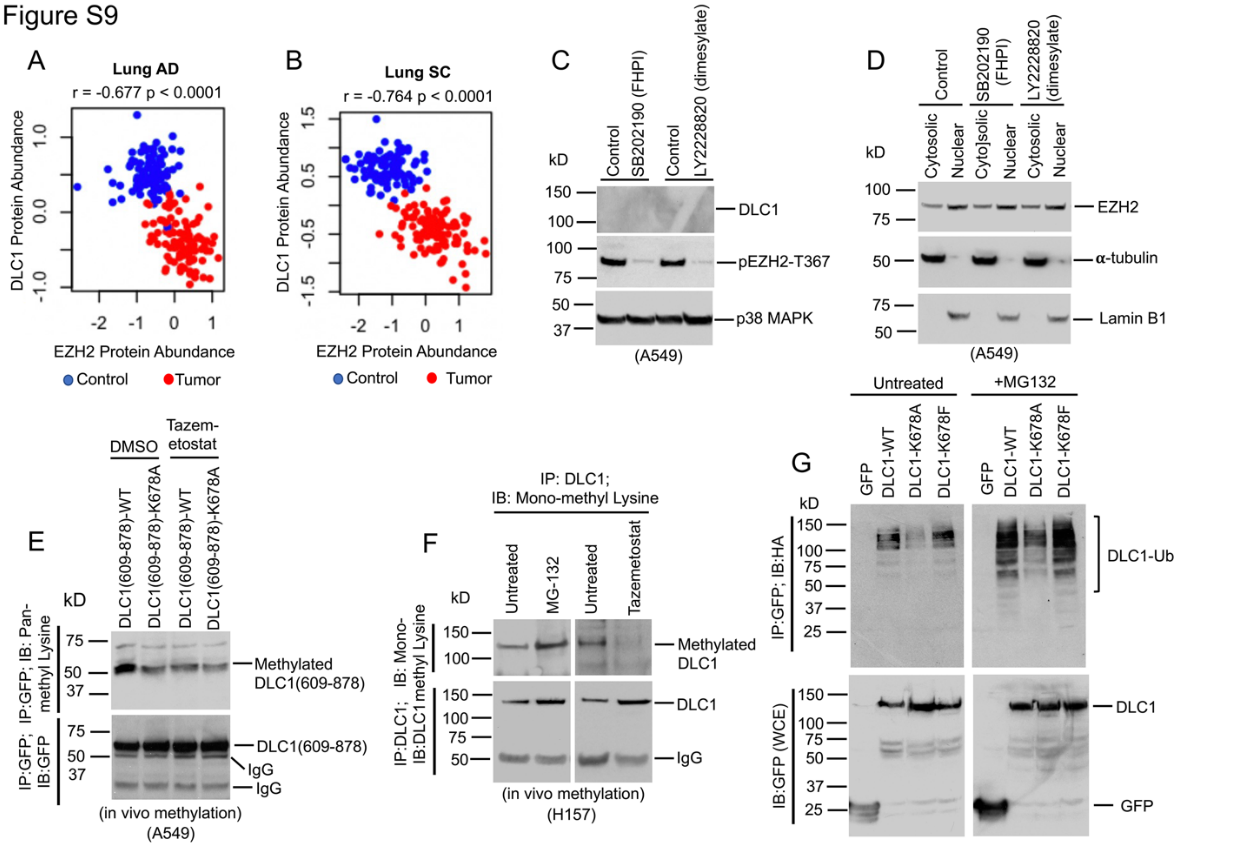

Supplementary Fig. 9. Inverse correlation between EZH2 protein and DLC1 protein in CPTAC lung cancer datasets; Monomethylation of DLC1 depends on EZH2 and leads to ubiquitination. (A-B) Inverse correlation between EZH2 protein abundance and DLC1 protein abundance in CPTAC datasets with (A) Lung adenocarcinoma (LUAD) and (B) Lung squamous cell carcinoma (Lung SC). • Tumor • Control. The correlation was plotted, and for the statistical analysis, the Pearson correlation coefficient (r) was measured using statistical computing and graphic software R (version 4.1.0). $p<0.0001$ for both A and B. (C-D) The cytoplasmic localization of EZH2 in A549 cells is not dependent on EZH2-pT367 phosphorylation. A549 cells were treated with two p38 MAPK inhibitors, SB202190 and LY2228820, which reduced EZH2-pT367. However, SB202190 and LY2228820 treatment did not lead to detectable DLC1 protein (C) or reduce the level of cytoplasmic EZH2 (D). Purity of nuclear (Lamin-B1) and cytosolic (α-Tubulin) fractionation of A549 cells was confirmed by the marker proteins. (E) In vivo methylation of DLC1-K678 is EZH2-dependent. A549 cells were transiently transfected with DLC1(609-878)-WT or DLC1(609-878)-K678A mutant and treated with Tazemetostat. Lysates were IP with GFP antibodies followed by IB with Pan-methyl lysine (top) or GFP (bottom) antibodies. Tazemetostat treatment reduced methylation of DLC1(609-878)-WT to that of DLC1(609-878)-K678A mutant and did not reduce the methylation signal of DLC1-K678A mutant. (F) Monomethylation occurs in endogenous DLC1 protein and depends on EZH2. Lysates from H157 cells treated with MG-132 or Tazemetostat were IP with DLC1 antibodies followed by IB with monomethyl lysine (top) or DLC1 (bottom) antibodies. MG-132 treatment of H157 cells increased their DLC1 monomethylation signal (top left), while Tazemetostat treatment decreased it (top right). (G) Monomethylation of DLC1-K678 is associated with

ubiquitination of K678, as DLC1-WT and methylated mimetic K678F mutant was strongly ubiquitinated in the presence of proteasome inhibitor MG-132, while the ubiquitinated signals in the methylated-deficient DLC1-K678A mutant were much weaker. Two independent experiments were performed for each C-G with similar results. Source data are provided as a Source Data file.
