# Supplementary material for: Inhibition of cytoplasmic EZH2 induces antitumor activity through stabilization of the DLC1 tumor suppressor protein
Source: Nat Commun. 2021 Dec 3;12:6941. doi: 10.1038/s41467-021-26993-3 (PMC8642553; doi:10.1038/s41467-021-26993-3)
Supplement: Supplementary file 2 — Reporting Summary [file 41467_2021_26993_MOESM2_ESM.pdf]

nature research

Corresponding author(s): Dr. Douglas R. Lowy; Dr. Brajendra K. Tripathi

Last updated by author(s): October 18, 2021

Reporting Summary

Nature Research wishes to improve the reproducibility of the work that we publish. This form provides structure for consistency and transparency in reporting. For further information on Nature Research policies, see our Editorial Policies and the Editorial Policy Checklist.

Statistics

For all statistical analyses, confirm that the following items are present in the figure legend, table legend, main text, or Methods section.

n/a	Confirmed	
☐	☒	The exact sample size (*n*) for each experimental group/condition, given as a discrete number and unit of measurement
☐	☒	A statement on whether measurements were taken from distinct samples or whether the same sample was measured repeatedly
☐	☒	The statistical test(s) used AND whether they are one- or two-sided<br>*Only common tests should be described solely by name; describe more complex techniques in the Methods section.*
☒	☐	A description of all covariates tested
☒	☐	A description of any assumptions or corrections, such as tests of normality and adjustment for multiple comparisons
☐	☒	A full description of the statistical parameters including central tendency (e.g. means) or other basic estimates (e.g. regression coefficient) AND variation (e.g. standard deviation) or associated estimates of uncertainty (e.g. confidence intervals)
☐	☒	For null hypothesis testing, the test statistic (e.g. *F*, *t*, *r*) with confidence intervals, effect sizes, degrees of freedom and *P* value noted<br>*Give P values as exact values whenever suitable.*
☒	☐	For Bayesian analysis, information on the choice of priors and Markov chain Monte Carlo settings
☒	☐	For hierarchical and complex designs, identification of the appropriate level for tests and full reporting of outcomes
☐	☒	Estimates of effect sizes (e.g. Cohen's *d*, Pearson's *r*), indicating how they were calculated

Our web collection on statistics for biologists contains articles on many of the points above.

Software and code

Policy information about availability of computer code

Data collection	Confocal microscopy of fluorescent-labeled cells was performed using a microscope (LSM 780; Carl Zeiss) with an excitation wavelength of 488 nm to detect transfected GFP fusion proteins. Alexa Fluor probes were viewed with excitation wavelengths of 488 nm (Alexa Fluor 488) and 568 nm (Alexa Fluor 568). Images were made at RT using photomultiplier tubes with a Plan-Apochromat 63×/1.4 NA oil differential interference contrast objective lens.
Data analysis	The Nuclear and cytoplasmic distribution of proteins were analyzed by Imaris cell imaging software, version 9.7.2. Immunoblots were quantified by densitometric scanning using ImageJ software, version 1.53a. For the statistical analysis, parametric unpaired t test with Welch's correction was performed using Prism software (version 9.1.2 (225); GraphPad).

For manuscripts utilizing custom algorithms or software that are central to the research but not yet described in published literature, software must be made available to editors and reviewers. We strongly encourage code deposition in a community repository (e.g. GitHub). See the Nature Research guidelines for submitting code & software for further information.

Data

Policy information about availability of data

All manuscripts must include a data availability statement. This statement should provide the following information, where applicable:

- Accession codes, unique identifiers, or web links for publicly available datasets
- A list of figures that have associated raw data
- A description of any restrictions on data availability

All data generated or analyzed during this study are included in this published article and its supplementary information files. Clinical Proteomic Tumor Analysis Consortium (CPTAC) data portal is downloaded from (https://proteomics.cancer.gov/data-portal). All the other data are available within the article and its supplementary information.

Field-specific reporting

Please select the one below that is the best fit for your research. If you are not sure, read the appropriate sections before making your selection.

☒ Life sciences ☐ Behavioural & social sciences ☐ Ecological, evolutionary & environmental sciences

For a reference copy of the document with all sections, see nature.com/documents/nr-reporting-summary-flat.pdf

Life sciences study design

All studies must disclose on these points even when the disclosure is negative.

Sample size	At least two independent experiments were performed for all in vitro experiments and at least four animals (n=4) were used per group for all in vivo experiments. Sample sizes were determined based on our experience with the specific type of experiment and commonly used sample sizes in ours and others previous publications within this field of research (Tripathi et al., 2017, Journal of Cell Biology; Tripathi et al., 2019, Journal of Cell Biology). The sample sizes and number of repeats are also defined in each figure legends. Immunoblots were quantified by densitometric scanning using ImageJ software 1.53a. Results are expressed as mean ± standard deviation (SD) from two or three experiments. All experiments were designed with matched control conditions within each experiment. For the statistical analysis, parametric unpaired t test with Welch's correction was performed using Prism software (version 9.1.2 (225); GraphPad).
Data exclusions	No data were excluded from the analysis.
Replication	At least two independent experiments were performed for all in vitro experiments. Experimental findings were reliably reproduced and all attempt at replication were successful. Results in bar graphs are displayed as mean ± standard deviation (SD) from two or three experiments. All experiments were designed with matched control conditions.
Randomization	All animal experiments were grouped randomly based on genetically related cohorts and tumor size. When tumors were approximately 0.5 cm in diameter, mice were randomly divided into groups and were treated with drugs.
Blinding	The investigators were blinded to group allocation during data collection and/or analysis.

Reporting for specific materials, systems and methods

We require information from authors about some types of materials, experimental systems and methods used in many studies. Here, indicate whether each material, system or method listed is relevant to your study. If you are not sure if a list item applies to your research, read the appropriate section before selecting a response.

Materials & experimental systems

n/a	Involved in the study	
☐	☒	Antibodies
☐	☒	Eukaryotic cell lines
☒	☐	Palaeontology and archaeology
☐	☒	Animals and other organisms
☒	☐	Human research participants
☒	☐	Clinical data
☒	☐	Dual use research of concern

Methods

n/a	Involved in the study	
☒	☐	ChIP-seq
☒	☐	Flow cytometry
☒	☐	MRI-based neuroimaging

Antibodies

Antibodies used	The following antibodies were purchased from Cell Signaling Technology. The catalogue number for each antibody is in the parentheses: AKT (4691), phospho-AKT-pS473 (4060), SRC (2108), phospho-SRC-pY416 (2101), EZH2 (5246), p38 MAPK (8690), Mono-Methyl Lysine (14679), CUL4A (2699), DDB-1 (5428), VPRBP (D5K5V; 14966) and GAPDH (2118). Two DLC1 antibodies were used: one, generated in our laboratory (DLC1 antibody; clone 428) and the other, DLC1 mouse mAb (612021, Clone 3/DLC-1 RUO), purchased from BD Biosciences. Annexin V (ab14196), ⍰-Galactosidase (ab116), KRAS (ab180772), alpha-Tubulin (ab4074), Lamin B1 (ab65986), Pan-methyl Lysine (ab7315), NEDD8 (ab81264), DCAF1 (ab53616), GFP mouse (ab1218), and GFP rabbit (ab290), antibodies were purchased from Abcam. RhoA (ARH04) antibodies were obtained from Cytoskeleton, Inc. EZH2-Phospho-Thr367 (12868) and Phospho-EZH2 (Thr367) Polyclonal (PA5-106225) were purchased from Signalway Antibody and Thermo Fisher Scientific, respectively. Cullin 4A rabbit monoclonal antibody (JU07-33, Cat. #MA5-34615) was purchased from Thermo Fisher Scientific. Ras (G12D) mouse monoclonal antibody (26036) was purchased from Vita Scientific. FBXW5 antibody (NBP3-04806) was purchased from Novus Biological, Inc. KRAS (WH0003845M1) and Actin (A4700) antibodies produced in mouse was purchased from Sigma Aldrich. Anti-rabbit (NA934V) and anti-mouse (NXA931V) IgG horseradish peroxidase-linked secondary antibodies were purchased from GE Healthcare. Alexa Fluor 568 anti-rabbit IgG (A10042), Alexa Fluor 488 anti-mouse IgG (A11001), and DAPI were purchased from Thermo Fisher Scientific.
Validation	We have used and validated most of the antibodies that are listed here in our previous studies (Tripathi et al., Journal of Cell Biology,

Validation	2014; Tripathi et al., Journal of Cell Biology, 2017; Tripathi et al., Journal of Cell Biology, 2019). For validation of new antibodies, we have used antibody profiles of online databases from the company site and the relevant citation for each primary antibody. The new antibodies were validated and used according to manufacturer's instructions.

Eukaryotic cell lines

Policy information about cell lines

Cell line source(s)	HEK 293T and human fibroblastic H1634 cells were cultured in DMEM supplemented with 10% FBS. NSCLC lines H1703, H157, A549, and H358, provided by Dr. C. Harris, Principal Investigator at the National Cancer Institute, Bethesda, MD, which were originally obtained from ATCC. NCI-H23, NCI-H460, SW900 were procured from ATCC. All cancer cell lines were cultured in RPMI-1640 supplemented with 10% FBS. All cells were cultured at 37°C in a humidified atmosphere of 95% air and 5% CO2.
Authentication	We have used the reliable sources for each cell lines, such as ATCC, but we did not authenticated.
Mycoplasma contamination	All cell lines tested negative for mycoplasma contamination.
Commonly misidentified lines (See ICLAC register)	None

Animals and other organisms

Policy information about studies involving animals; ARRIVE guidelines recommended for reporting animal research

Laboratory animals	The mouse studies were approved by the National Cancer Institute Animal Care and Use Committee and conducted in compliance with the approved protocols. NOD.SCID/NCR mice were obtained from Charles River Laboratory, National Cancer Institute centralized animal order system. We used 6-8 weeks old mice for this study. The animals were housed under standard laboratory conditions in 12 hours dark/light cycle (6 am to 6 pm) at ambient temperature 68-76F with 30%-70% humidity and were provided continuous food and water supply.
Wild animals	The study did not involve wild animals.
Field-collected samples	No field collected samples were used in the study
Ethics oversight	The mouse studies were approved by the National Cancer Institute Animal Care and Use Committee and conducted in compliance with the approved protocols. Animals were housed under standard laboratory conditions and water and food were continuously available.

Note that full information on the approval of the study protocol must also be provided in the manuscript.
