# Supplementary material for: Inhibition of cytoplasmic EZH2 induces antitumor activity through stabilization of the DLC1 tumor suppressor protein
Source: Nat Commun. 2021 Dec 3;12:6941. doi: 10.1038/s41467-021-26993-3 (PMC8642553; doi:10.1038/s41467-021-26993-3)

Fig. 1. Uncropped western blot data. Uncropped images of western blots in main Fig. 1a-c.

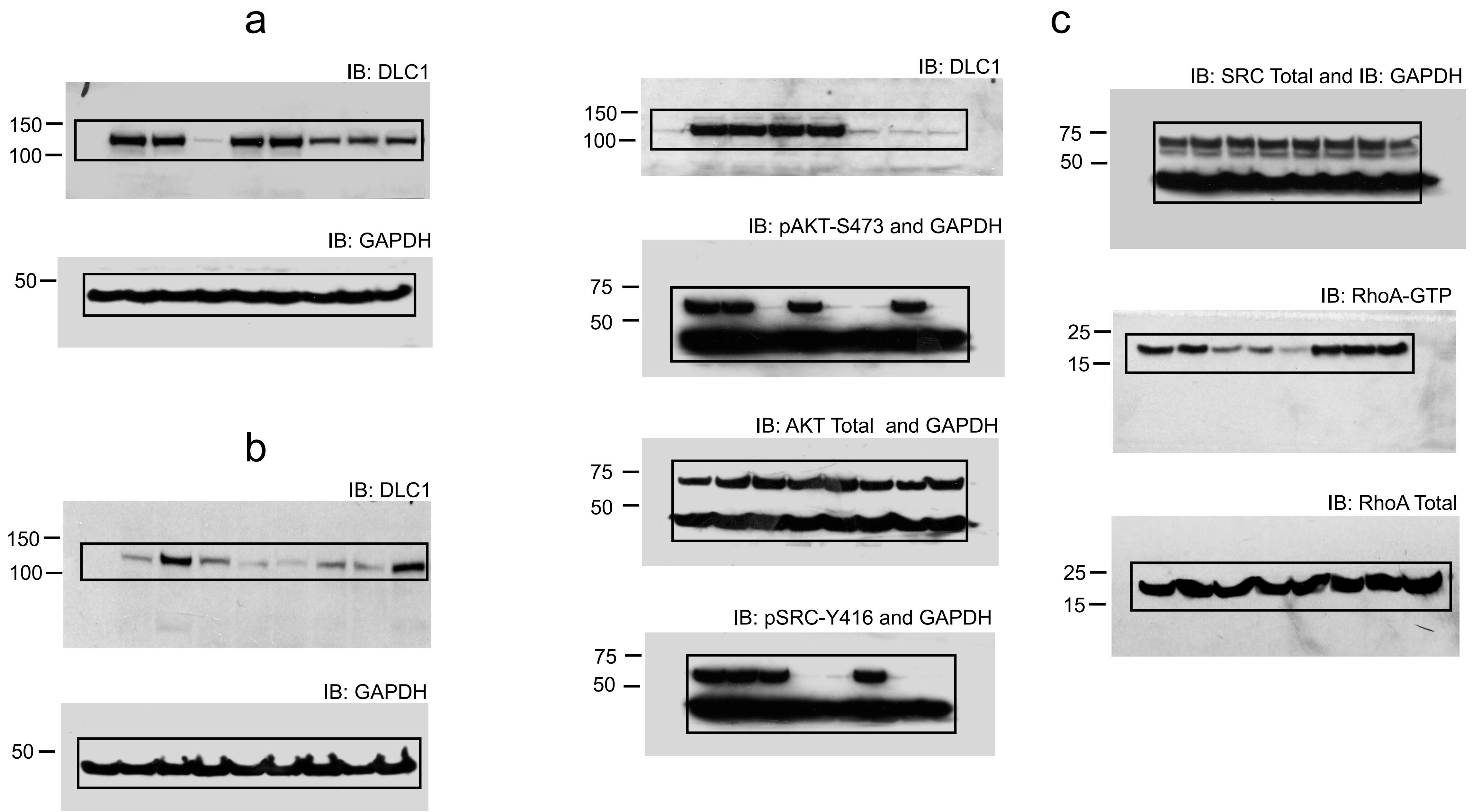

Fig. 1. Uncropped western blot data. Uncropped images of western blots in main Fig. 1d-e.

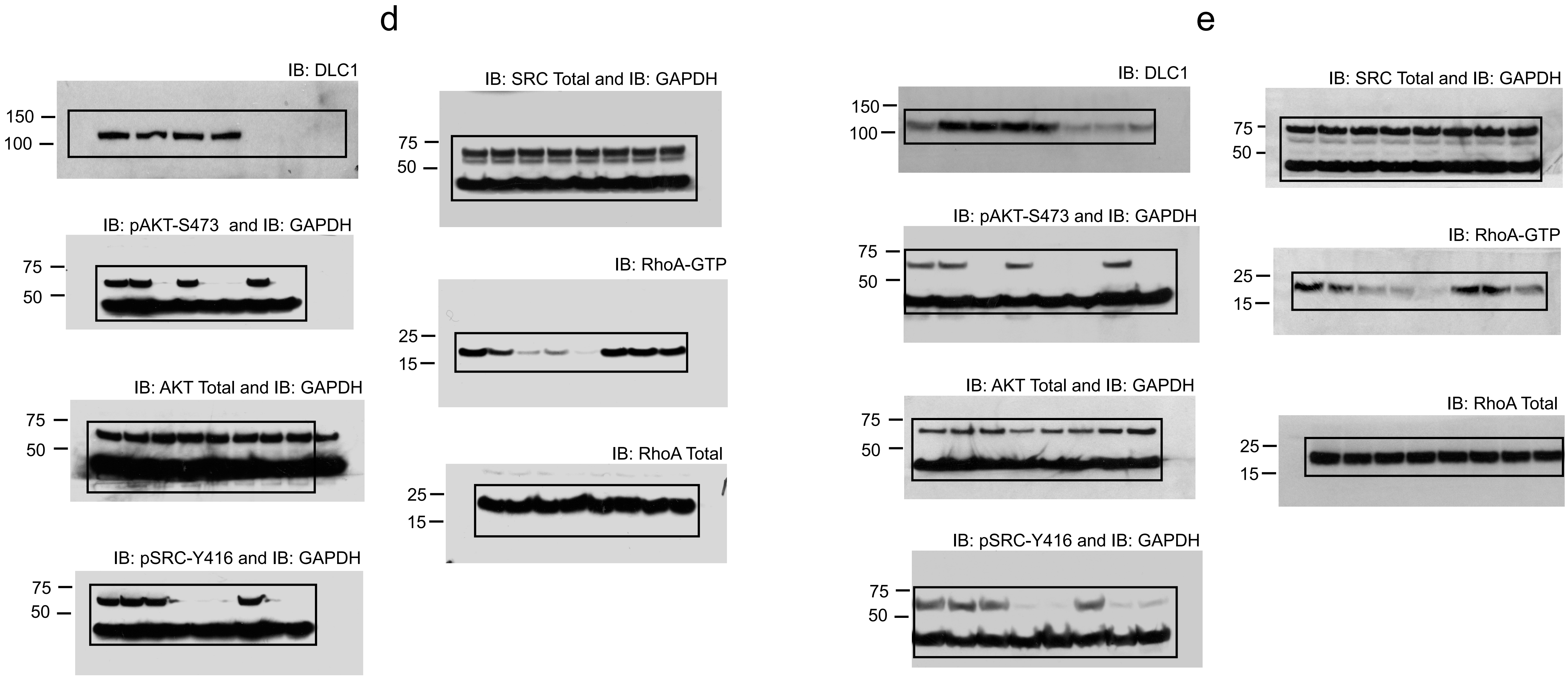

Fig. 2. Uncropped western blot data. Uncropped images of western blots in main Fig. 2d.

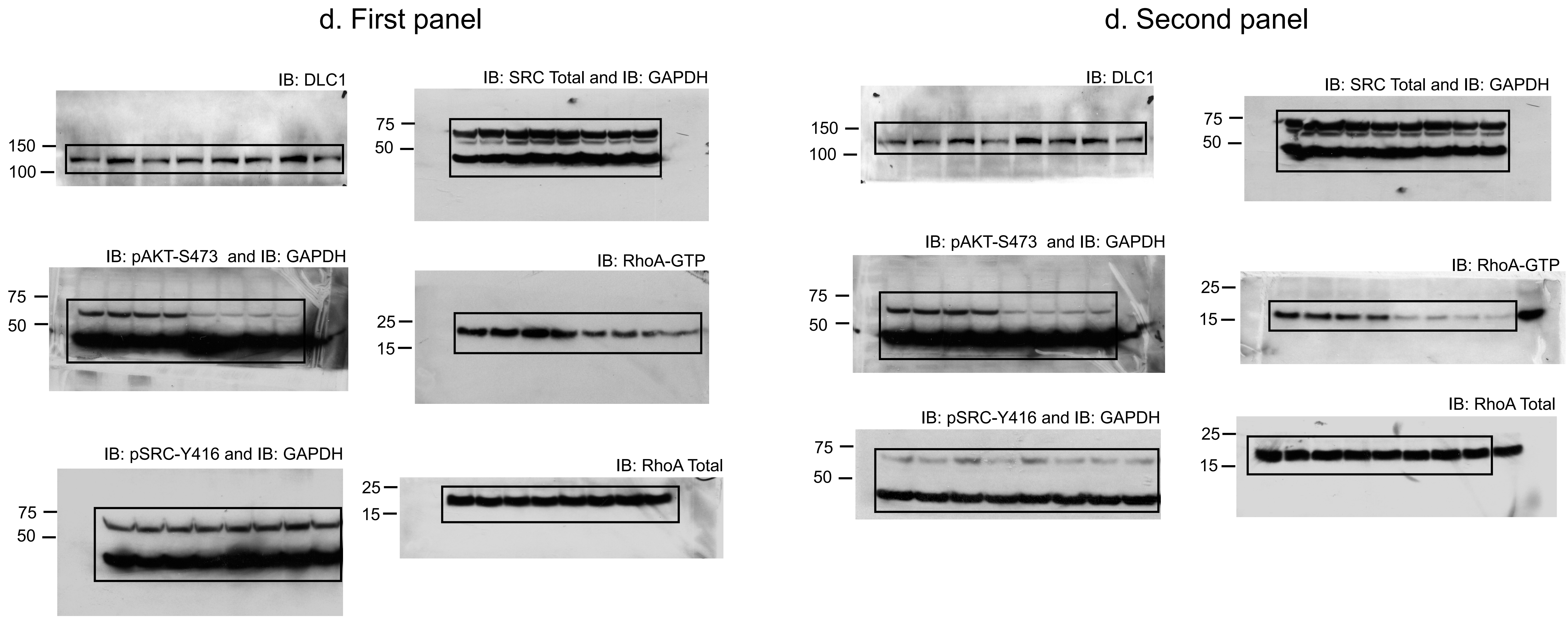

Fig. 2. Uncropped western blot data. Uncropped images of western blots in main Fig. 2d.

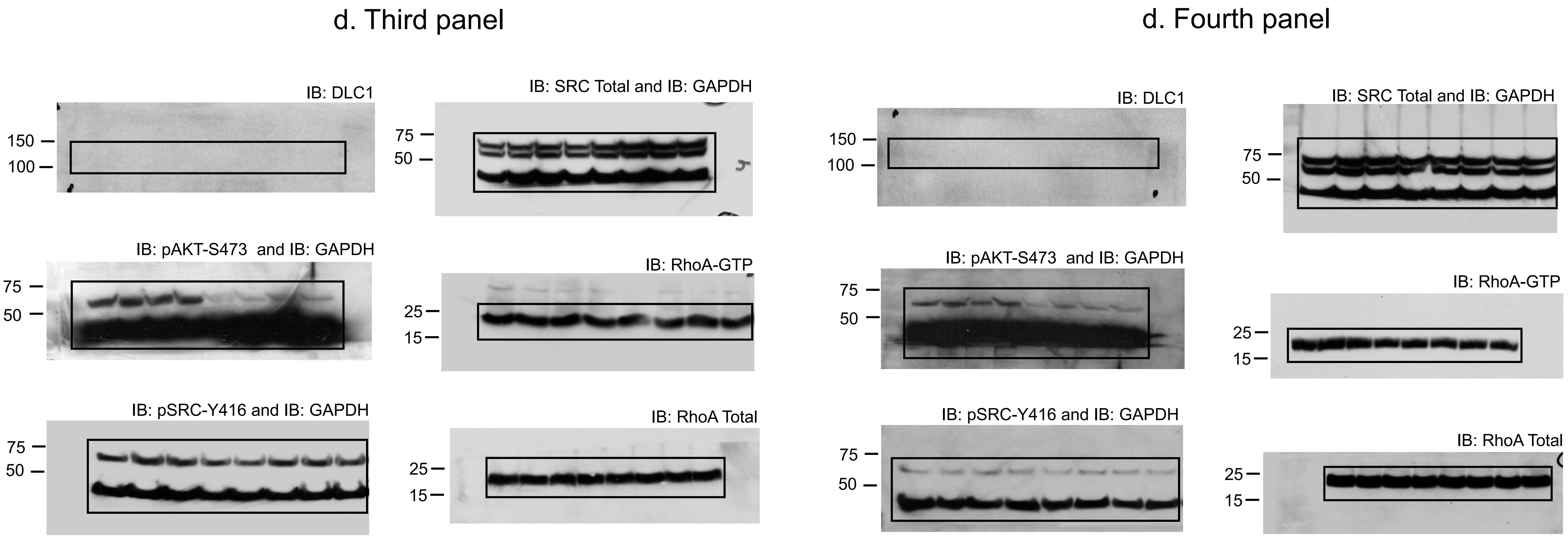

Fig. 3. Uncropped western blot data. Uncropped images of western blots in main Fig. 3d.

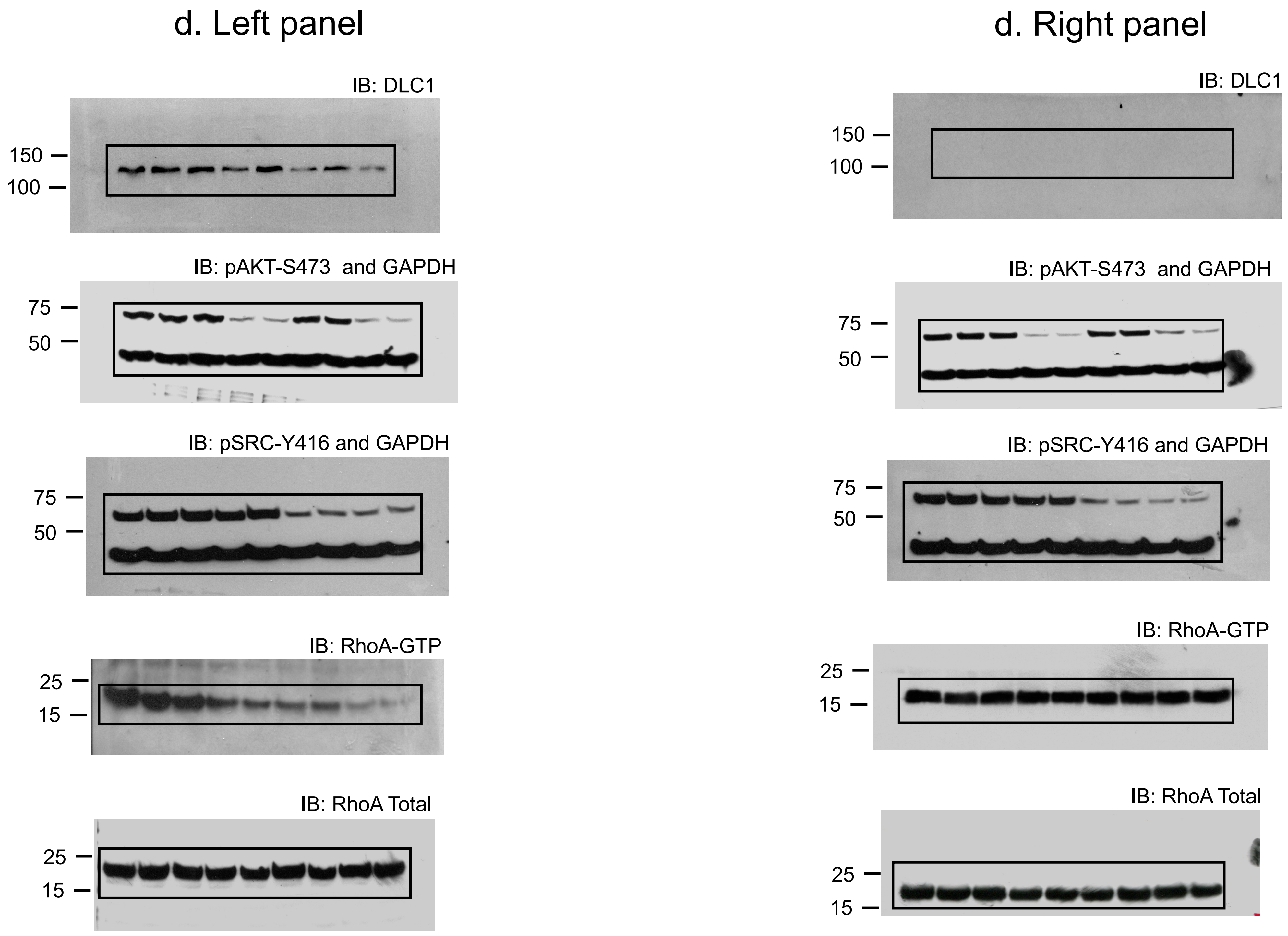

Fig. 4. Uncropped western blot data. Uncropped images of western blots in main Fig. 4a.

a

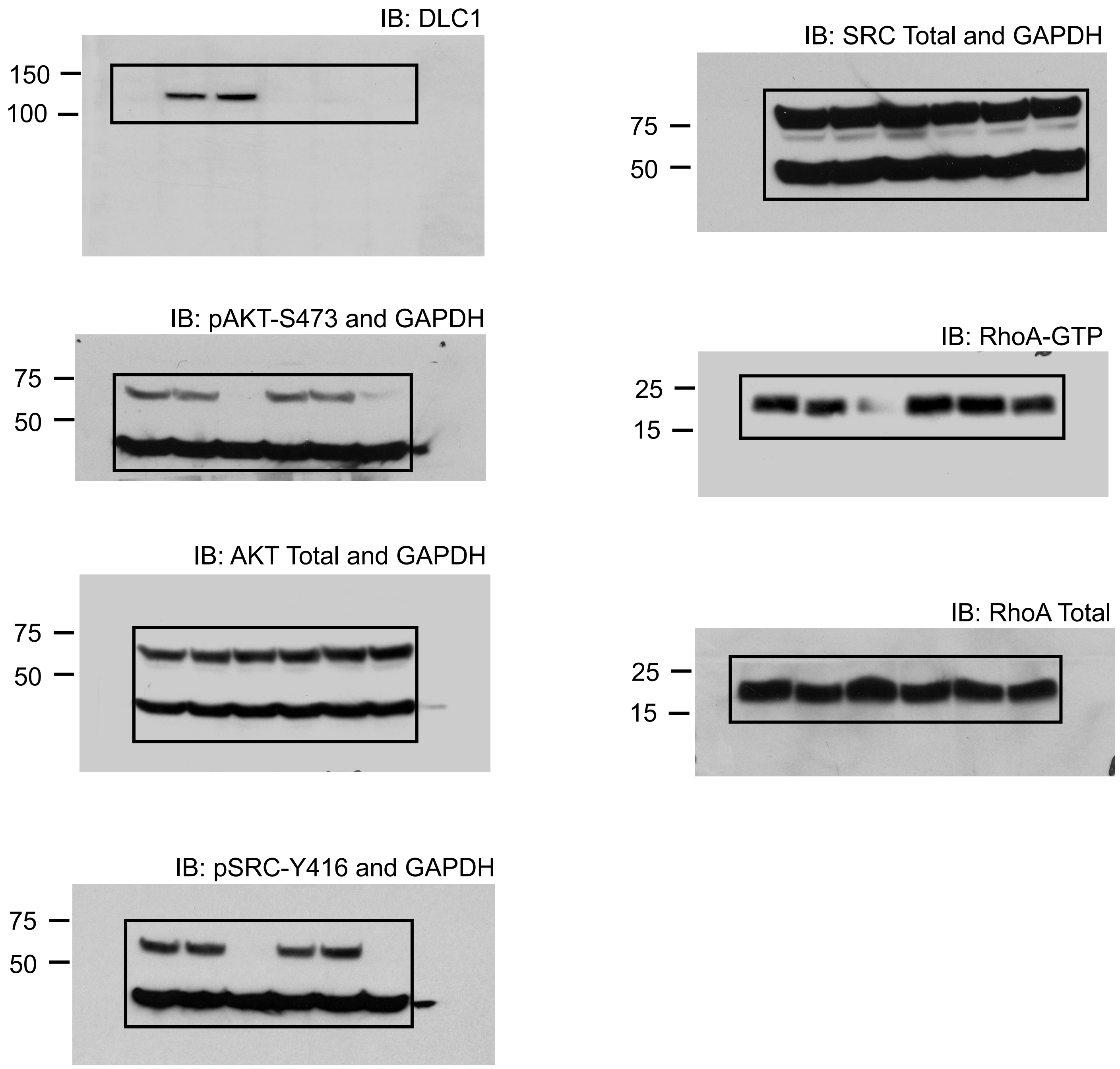

Fig. 5. Uncropped western blot data. Uncropped images of western blots in main Fig. 5a, c, e, g.

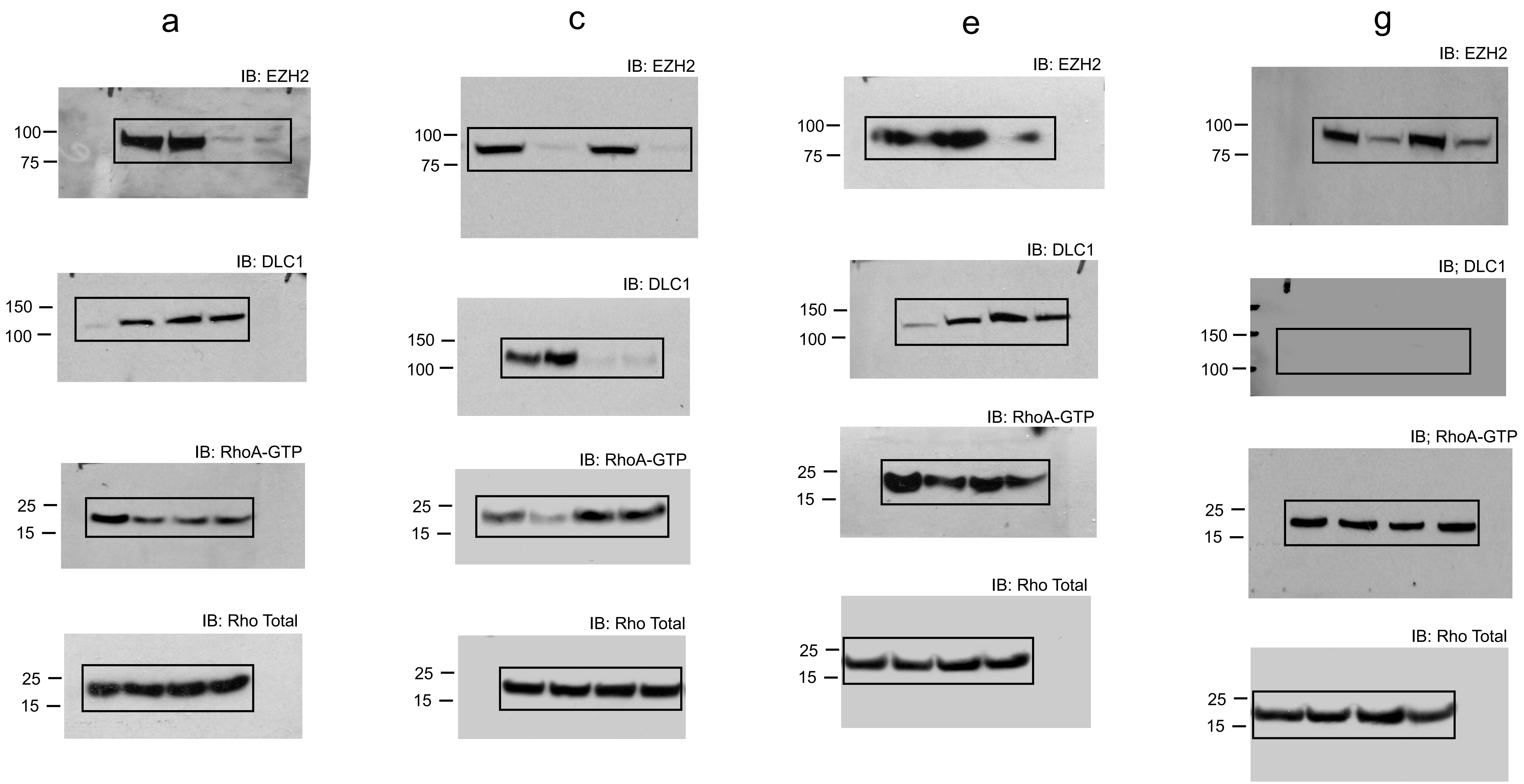

Fig. 6. Uncropped western blot data. Uncropped images of western blots in main Fig. 6a-h.

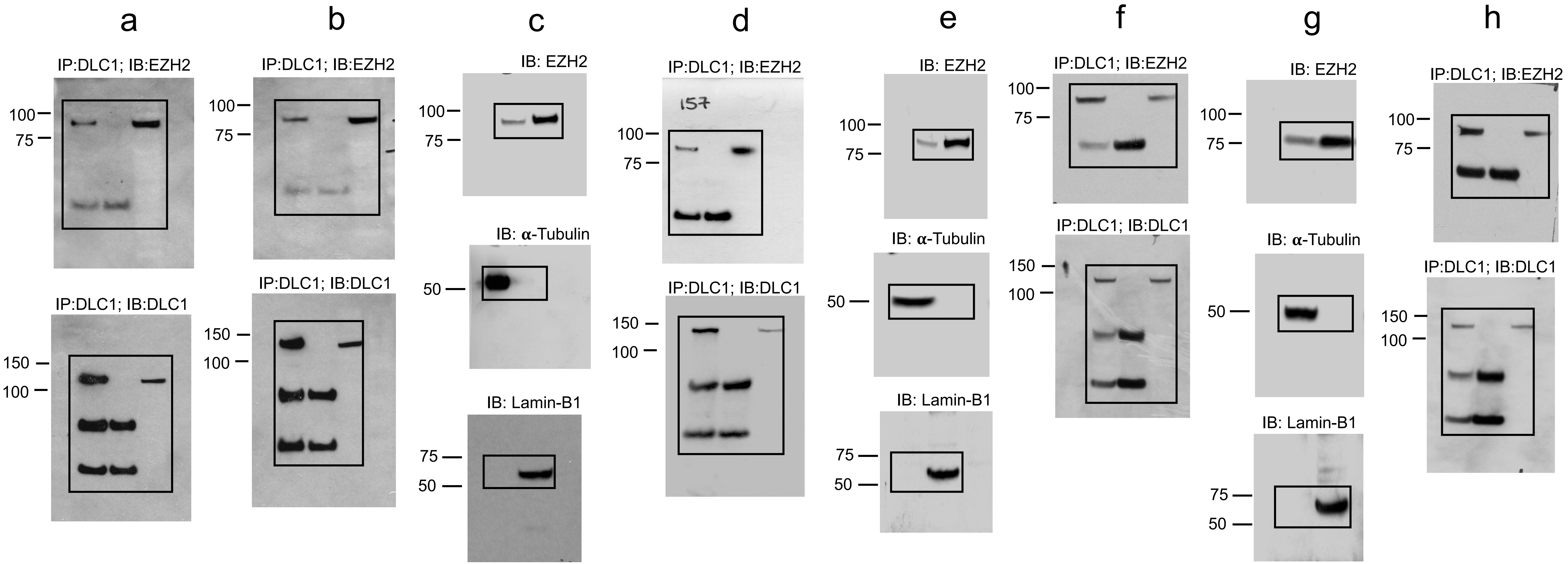

Fig. 7. Uncropped western blot data. Uncropped images of western blots in main Fig. 7d-g.

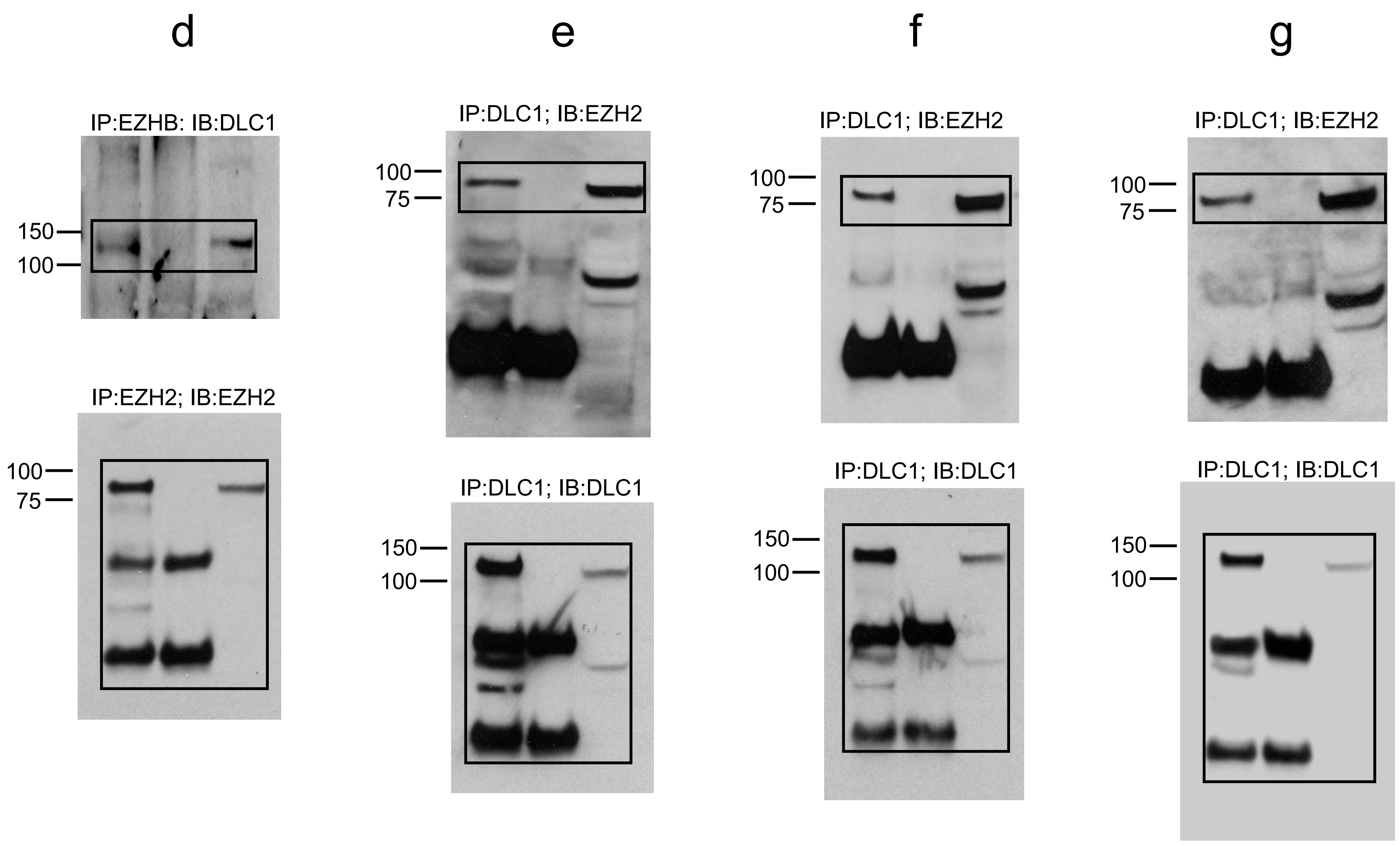

Fig. 8. Uncropped western blot data. Uncropped images of western blots in main Fig. 8b-h.

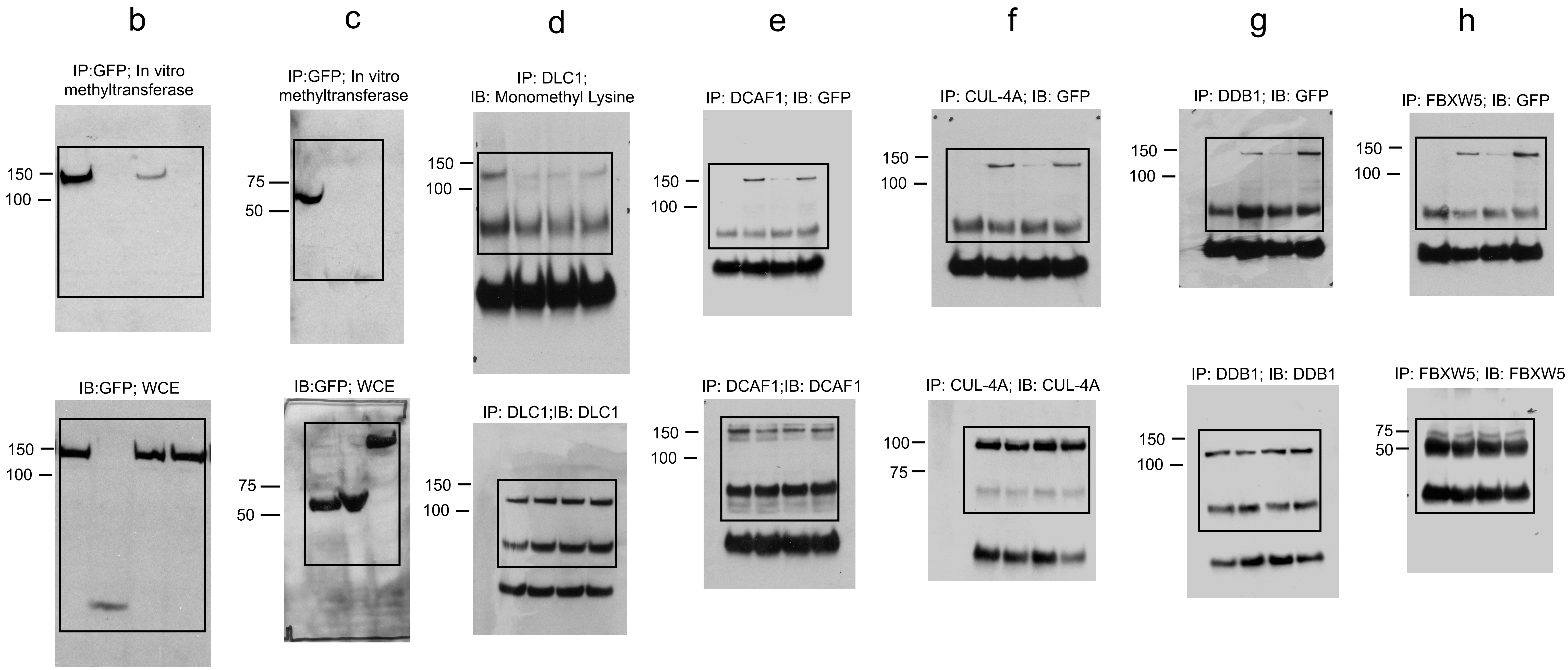

Fig. 9. Uncropped western blot data. Uncropped images of western blots in main Fig. 9a-f.

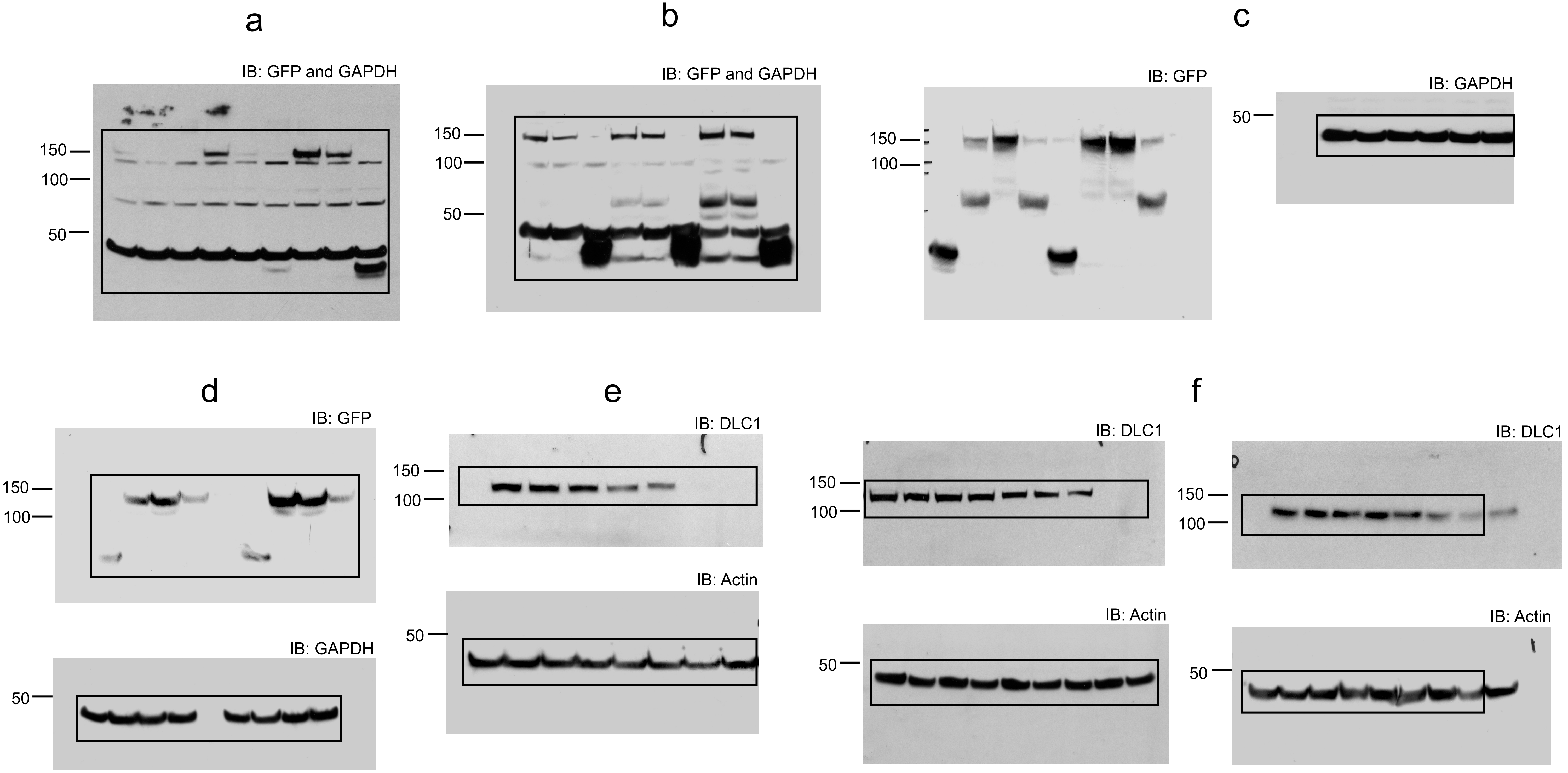

Fig. 10. Uncropped western blot data. Uncropped images of western blots in main Fig. 10a-f. 0

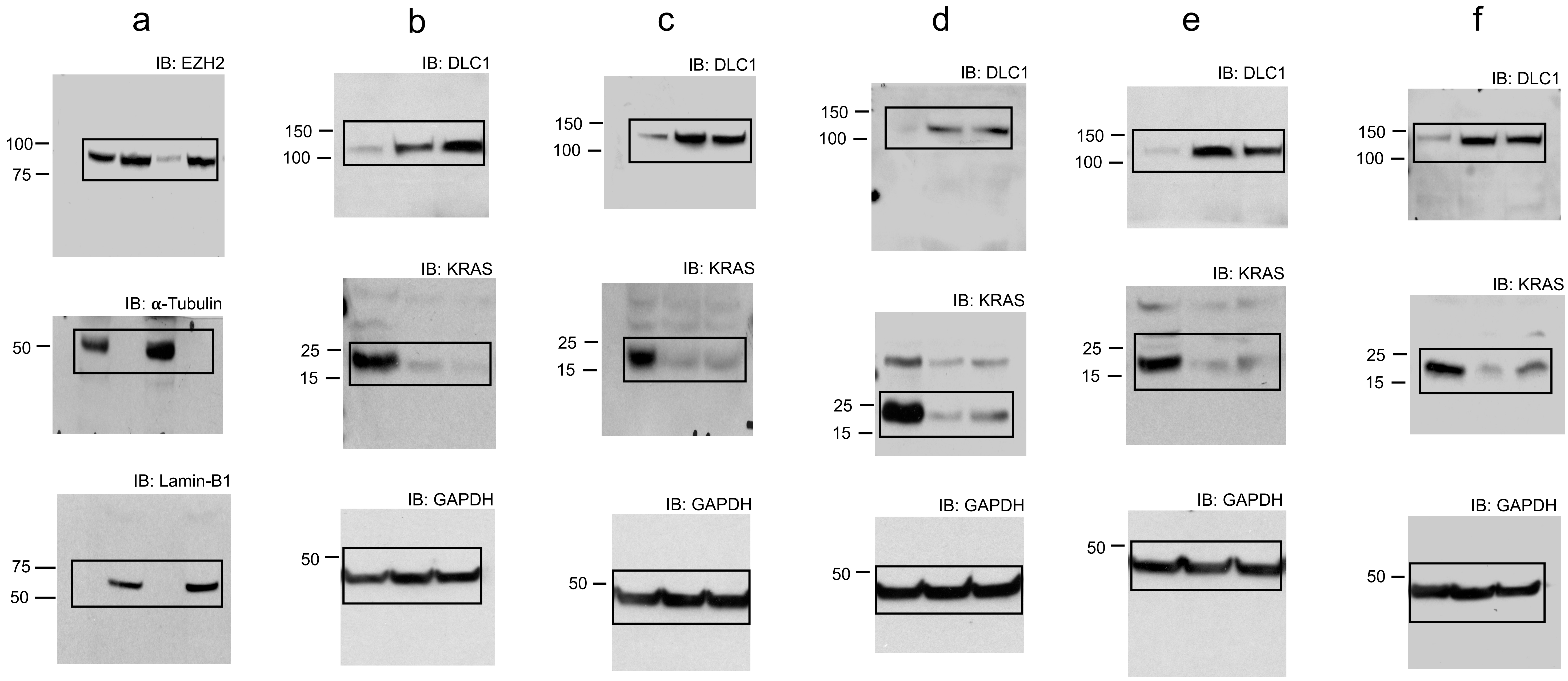

Fig. 10. Uncropped western blot data. Uncropped images of western blots in main Fig. 10g-i.

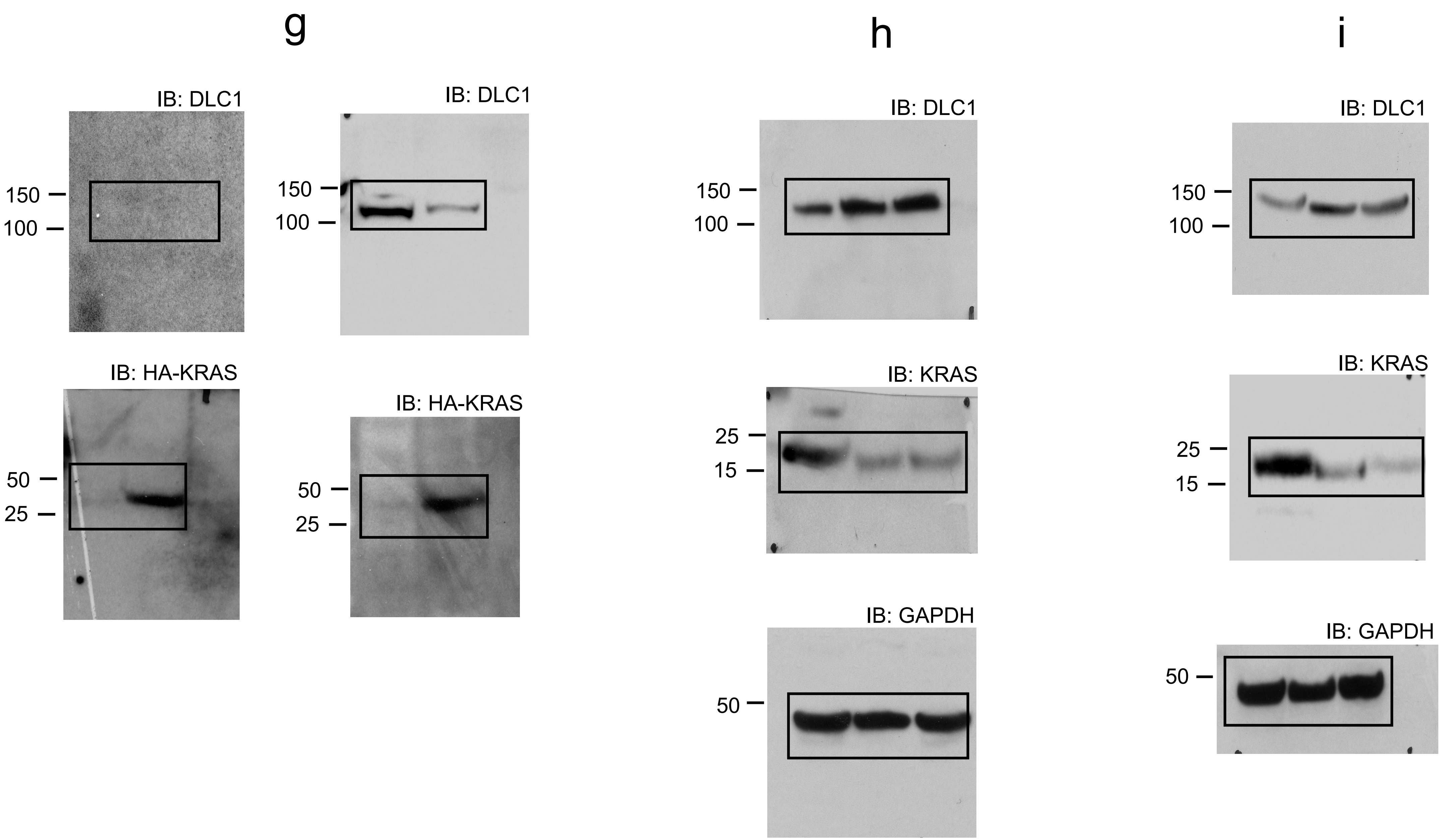

Fig. S1. Uncropped agarose gel and western blot data. Uncropped images of gel and blots in Supplementary Fig. S1B-E.

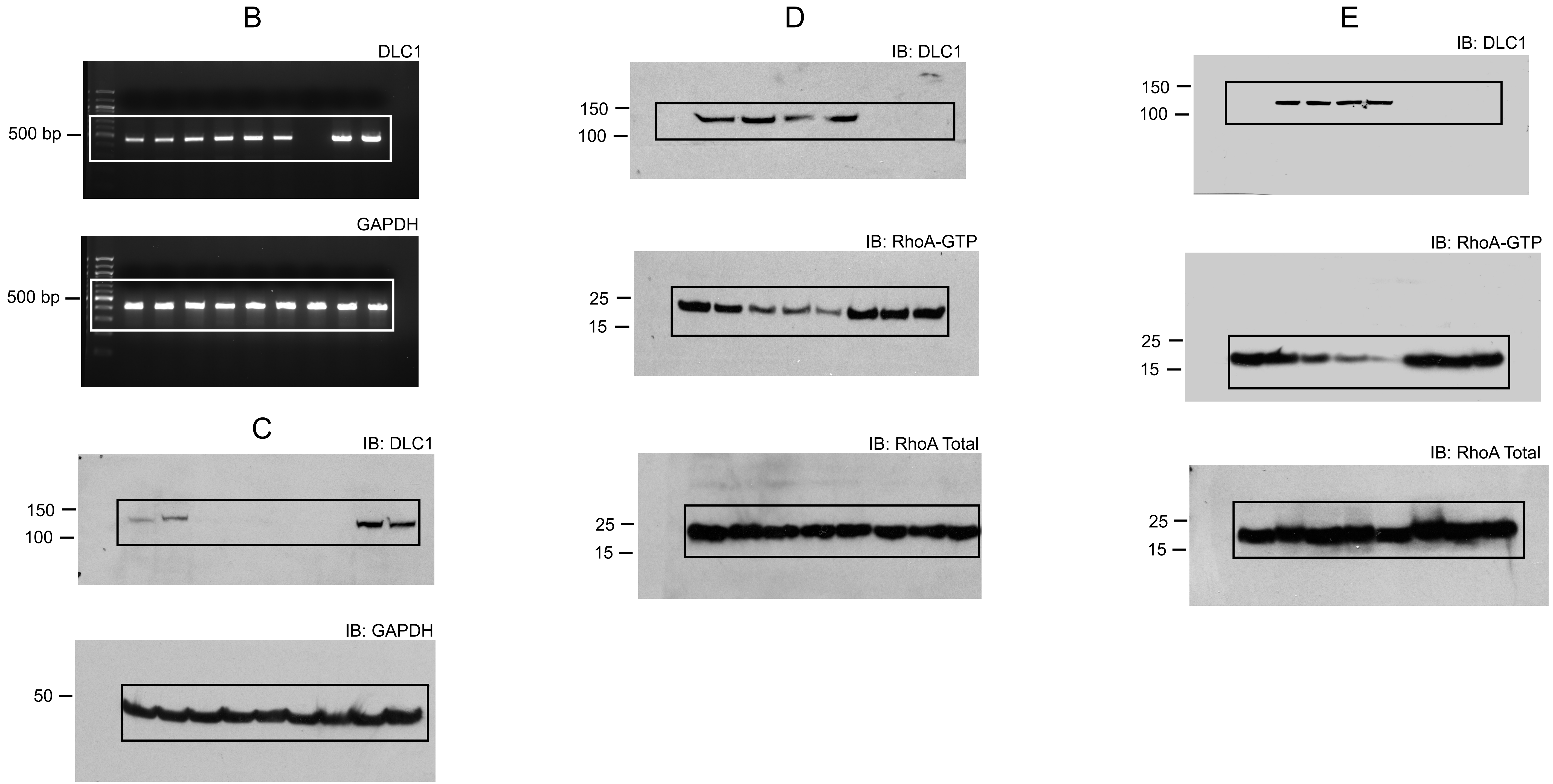

Fig. S1. Uncropped western blot data. Uncropped images of western blots in Supplementary Fig. S1F-G.

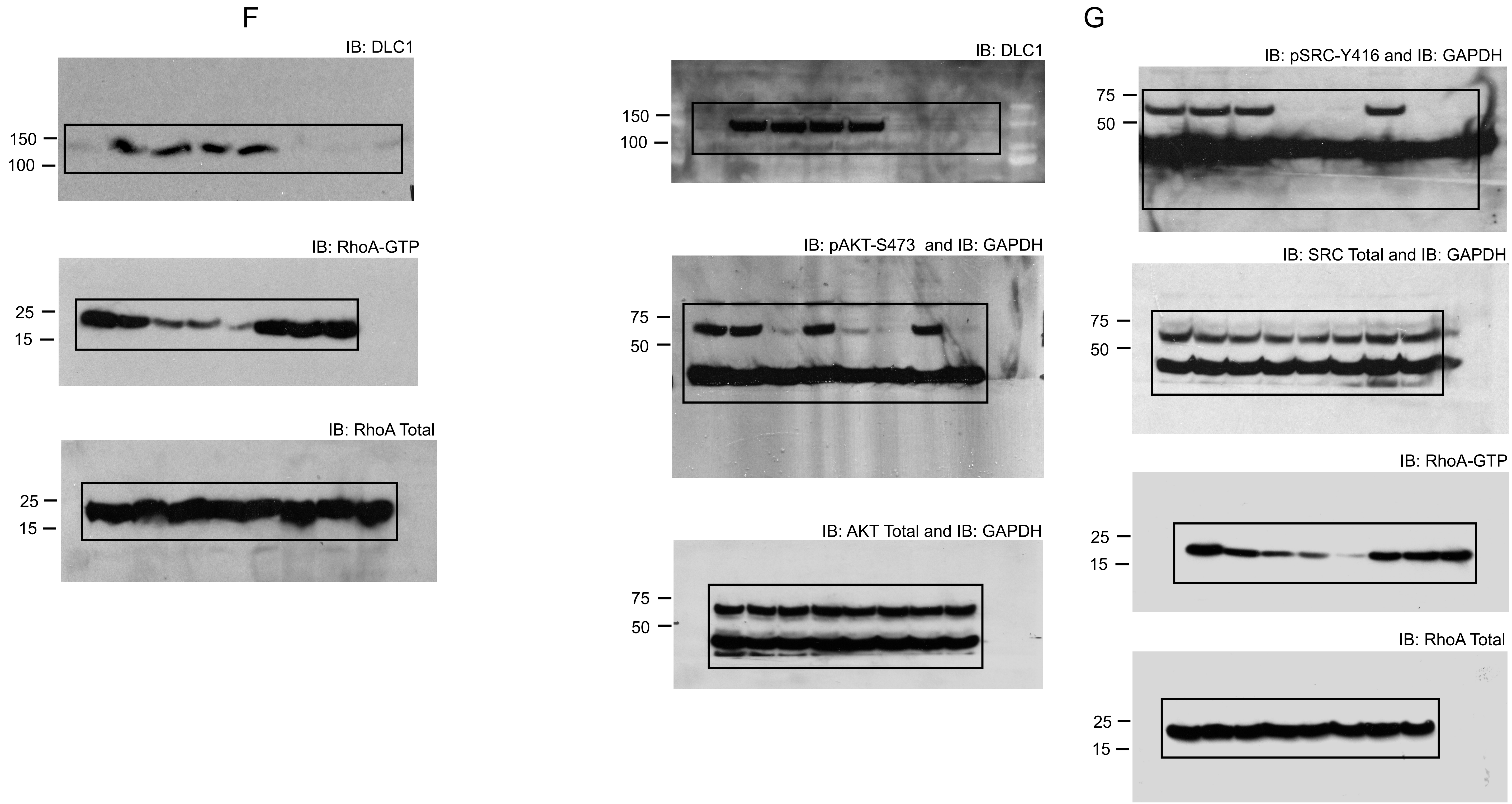

Fig. S7. Uncropped western blot data. Uncropped images of western blots in Supplementary Fig. S7A.

A

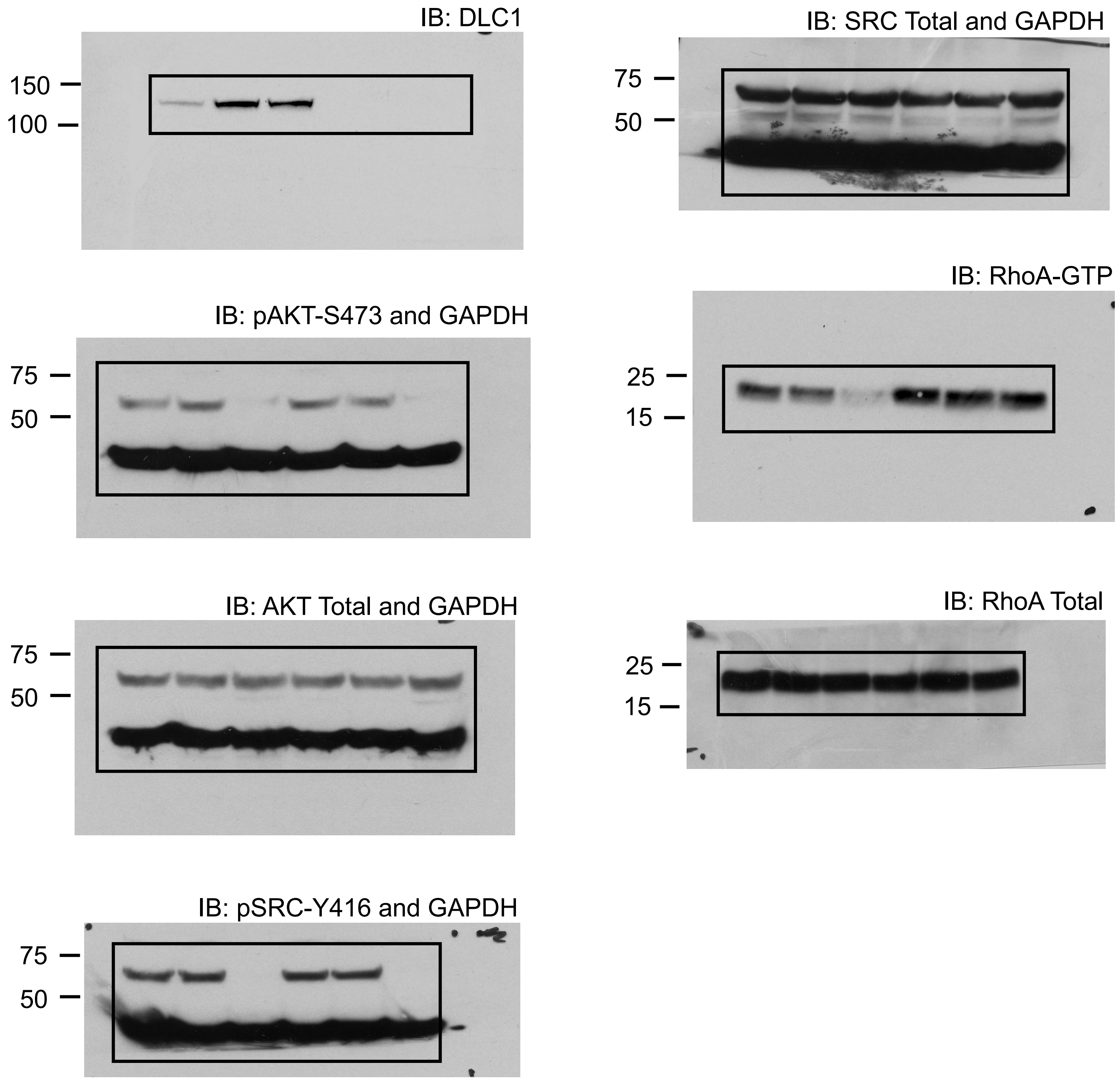

Fig. S9. Uncropped western blot data. Uncropped images of western blots in Supplementary Fig S9C-F.

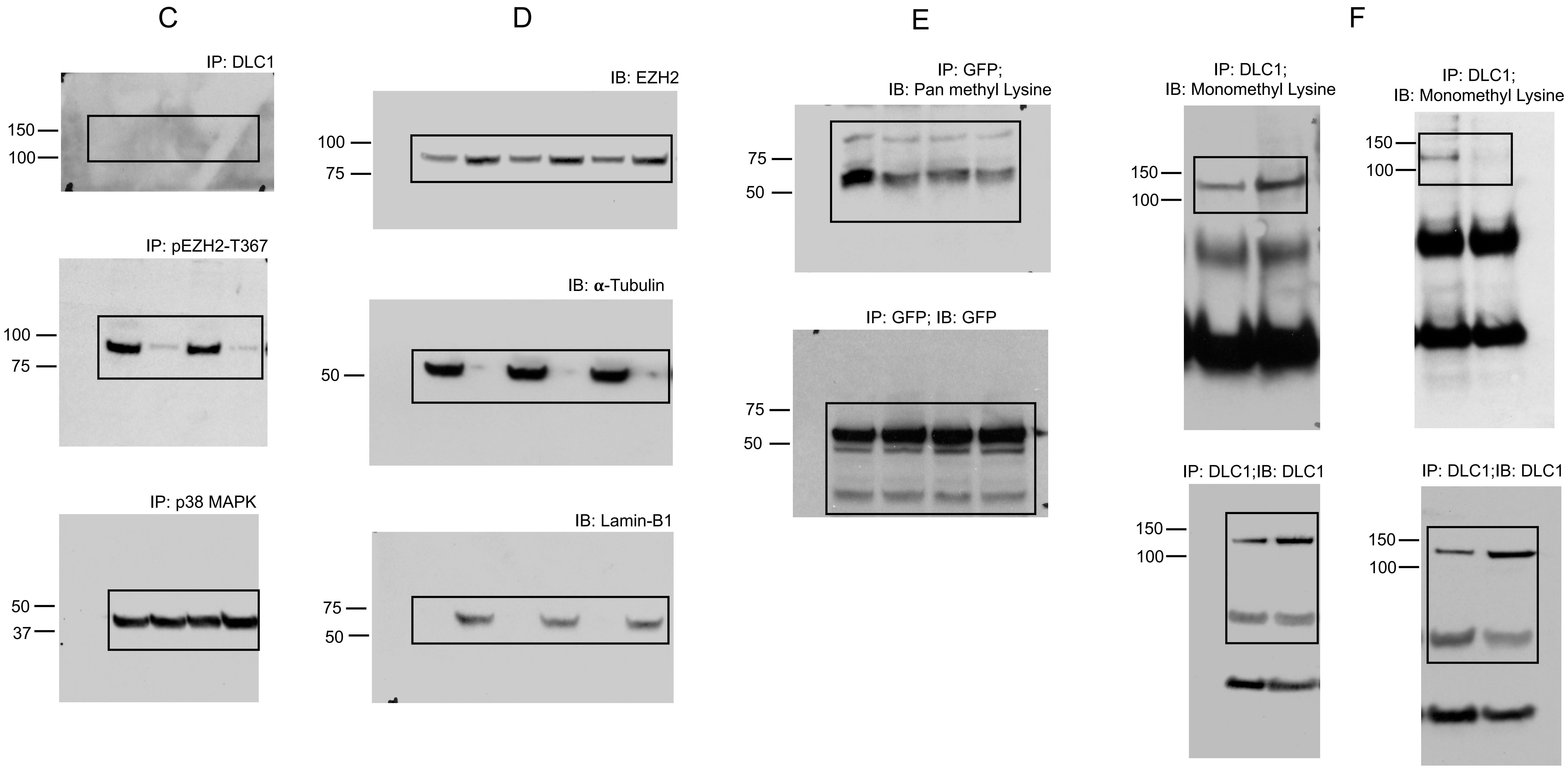

Fig. S9. Uncropped western blot data. Uncropped images of western blots in Supplementary Fig S9G.

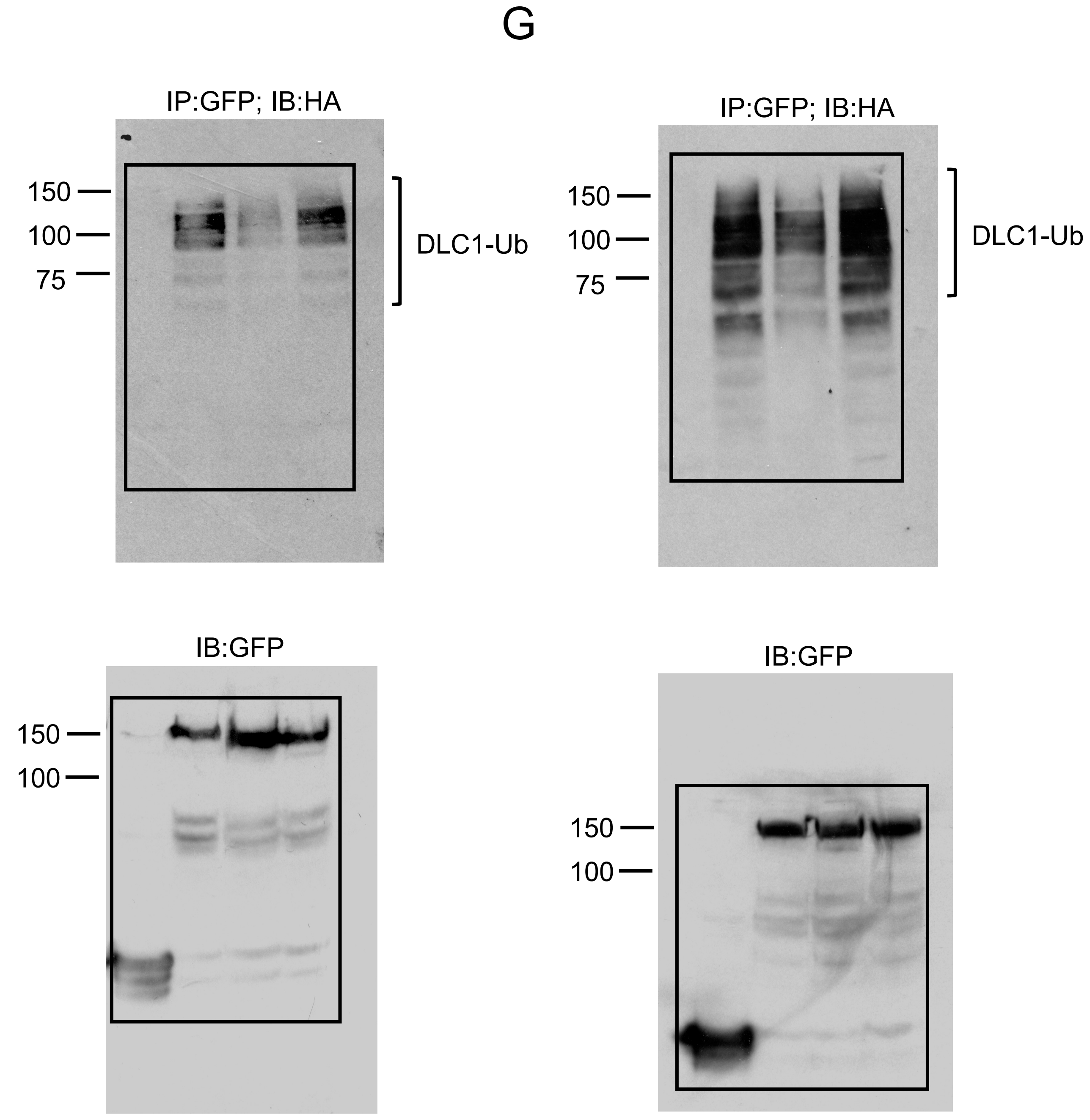

Supplement: Supplementary file 3 — Source Data [file 41467_2021_26993_MOESM3_ESM.zip › 297525_2_related_ms_5932096_r05m6z.pdf]
